# A Site-Specific Self-Association of a Protein Hub Drives Its Phase Separation

**DOI:** 10.1021/acschembio.5c00424

**Published:** 2025-12-24

**Authors:** Mohammad Ahmad, Yazheng Wang, Siddharth Krishnan, Ali Imran, Aleksei Aksimentiev, Liviu Movileanu

**Affiliations:** † Department of Physics, 2029 Syracuse University, 201 Physics Building, Syracuse, New York 13244, United States; ‡ Department of Biomedical and Chemical Engineering, Syracuse University, 329 Link Hall, Syracuse, New York 13244, United States; § Department of Physics, 14589 University of Illinois at Urbana−Champaign, Urbana, Illinois 61801, United States; ∥ The BioInspired Institute, Syracuse University, Syracuse, New York 13244, United States; ⊥ Department of Biology, Syracuse University, 114 Life Sciences Complex, Syracuse, New York 13244, United States

## Abstract

Liquid–liquid phase separation (LLPS) is pivotal in generating membraneless organelles and assembling cellular inclusions. Interactions mediated by RNA and intrinsically disordered regions of proteins are ubiquitous mechanisms that drive their LLPS. Here, we identify that a site-specific interaction stimulates the LLPS of WDR5, a chromatin-associated protein hub. Our study proves that WDR5 undergoes self-association between its N-terminal intrinsically disordered region and a multitasking binding site. This mechanism facilitates the formation of liquid droplets in a cell-free environment. Notably, WDR5 undergoes phase separation in mammalian cells, forming nuclear puncta (NP) in response to osmotic stress. Further, nuclear WDR5 condensates encompass a critical oncoprotein transcription factor, MYC, and WDR5-binding RNA under hyperosmotic conditions. Our findings suggest that RNA modulates WDR5 phase separation and influences nuclear puncta formation, potentially serving as a general stress response mechanism. These outcomes illuminate a distinctive mechanochemical signaling process, highlighting the functional interplay among WDR5, RNA, and MYC at the chromatin level, particularly during osmotically induced LLPS.

## Graphical abstract

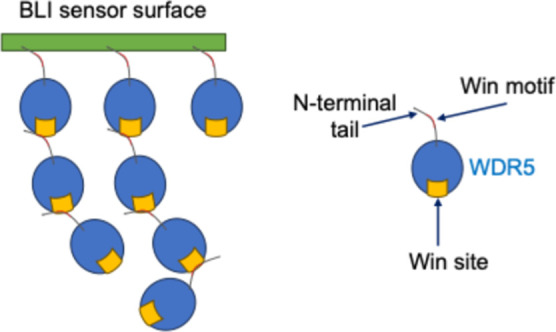

## Introduction

Liquid–liquid phase separation (LLPS) is essential for forming membraneless organelles.^1–4^ These subcellular entities are uniquely equipped with capacities to sequester and concentrate specific proteins and nucleic acids.^5–7^ LLPS has recently emerged as a crucial player in several critical functional processes in the nucleus under physiological^8,9^ and pathological^10,11^ conditions. For example, LLPS contributes to heterochromatin generation,^12,13^ augmentations of transcriptional activity,^14,15^ and the assembly of enhancer elements alongside their associated transcription-related proteins at specialized loci referred to as superenhancers.^16^ These superenhancers control the transcription of critical genes central to maintaining cell identity or influencing cancer aggressiveness. In eukaryotic cells, various stressors induce RNA-binding proteins (RBPs)^17–19^ to be assembled into stress granules^20,21^ and nuclear stress bodies.^22,23^

WD40 repeat protein 5 (WDR5) is an evolutionarily conserved multitasking protein hub ubiquitously expressed in all human tissues.^24–26^ WDR5 is localized into the nucleus to drive the transcription of genes crucial for proliferation by interacting with transcriptional regulators.^27,28^ It has been extensively characterized as a regulatory factor of the multisubunit mixed lineage leukemia (MLL/SET1) enzymatic complex involved in histone 3 lysine 4 (H3L4) methylation.^29,30^ Its expression is significantly amplified under oncogenic conditions,^31–33^ and dysregulation of its activity leads to accelerated cancer progression.^34–36^ As a nucleic acid-binding protein, WDR5 interacts with RNA^37,38^ and DNA,^24^ modulating gene expression. In addition, WDR5 forms a transient complex with the oncoprotein myelocytomatosis (MYC) transcription factor,^39,40^ facilitating its recruitment to the regulatory sequences of different targeted genes. Recent findings have also demonstrated the direct interaction of MYC with RNA.^41^ In different contexts,^42–44^ evidence supports the colocalization of WDR5 in phase-separated bodies. Yet, this protein extensively engages in reversible interactions with numerous regulatory proteins.^25,26,45^ Given its wide-ranging significance in diverse cellular processes,^46–48^ there is a pressing need for a quantitative understanding of the mechanisms leading to the phase separation of WDR5.

Here, we report that a site-specific interaction catalyzes the phase separation of WDR5. We show compelling evidence supporting that the underlying mechanism of the WDR5 condensate is the oligomerization and formation of a chain-like network of WDR5 molecules. This network results from the self-association between a motif within the N-terminal intrinsically disordered tail and one of the binding sites of WDR5. Such a mechanism significantly deviates from those involving biomolecular segregations brought about by multiple intrinsically disordered region (IDR)-mediated nonspecific interactions. This is because the LLPS of proteins and nucleic acids typically occurs in vitro and in cells due to weak and multivalent interactions among these molecules.^49–52^ Moreover, the self-association of WDR5 exhibits a low affinity, which is in accordance with the LLPS prerequisite and aligns with the behavior observed in other intrinsically disordered proteins undergoing phase separation. LLPS has been recognized as an adaptive cellular mechanism against challenging environmental conditions.^53^ This study shows that WDR5 forms liquid-like nuclear puncta (NP) of mammalian cells under hyperosmotic conditions. In addition, we show that WDR5 colocalizes with MYC condensates, enriching MYC in a cell-free environment and living cells. Therefore, this outcome highlights a distinctive mechanism of LLPS mediated by an RBP, revealing a supplementary signaling route that operates at the WDR5 and MYC levels under adverse conditions. We also demonstrate that these WDR5 condensates can accumulate a long non-coding RNA (lncRNA) fragment with a regulatory function in their assembly.

## Results and Discussion

### Direct Evidence for the WDR5 Self-Association

Using the AlphaFold 3 server,^54^ we obtained five all-atom structures of a WDR5 dimer. In the top-scored structure (Figure 1a), the N-terminal IDR tail of the first protein of the dimer was docked into the Win site^55–58^ of the second protein. Similarly, the IDR tail of the second protein was docked into the Win site of the first protein. Further, in the top-scored AlphaFold 3 structure of a WDR5 tetramer (Figure 1b), the IDR tails of individual WDR5 proteins form the same specific interactions with the Win pockets of the neighboring protein, producing a closed, chain-like arrangement. In both cases, the AR motif of the IDR tail was located within the Win pocket (Figure 1c). Interestingly, the top-scored AlphaFold 3 structure of the WDR5 dimer containing an Arg-to-Asp mutation at position 14 of the IDR tail (R14N-WDR5) did not exhibit such a specific tail-pocket interaction (Supplementary Figure S1a), suggesting that Arg-14 plays a key role in this specific interaction. The IDR tail-Win pocket binding was also predicted by docking calculations,^59^ which placed the Arg-14 residue within ∼3 Å from the center of the pocket in the highest-scored structure (Methods; Supplementary Figure S2).

**Figure 1:**
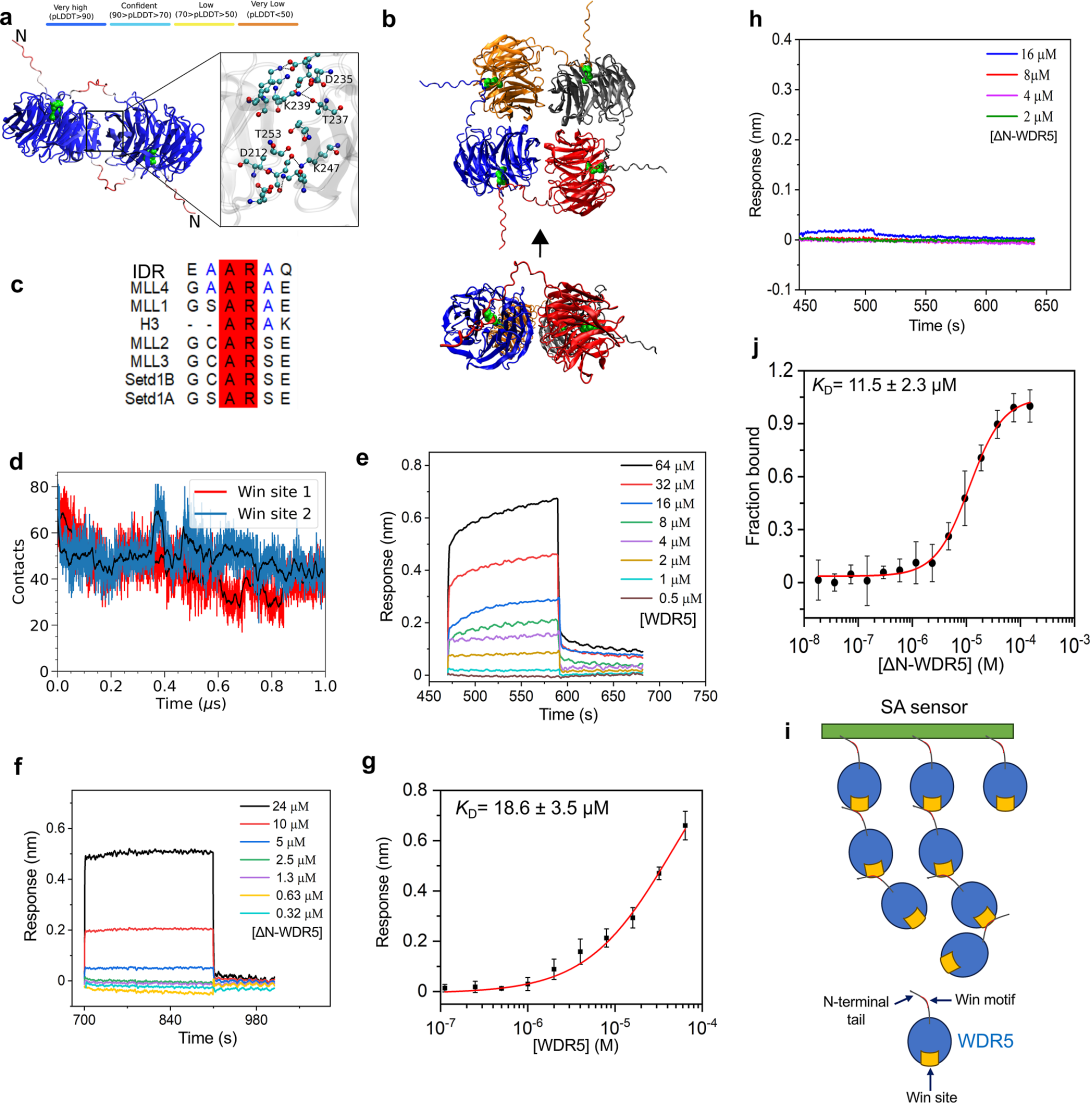
The self-association of WDR5. (a) The AlphaFold 3 model of a WDR5 dimer. The top-scored structure of the wild-type WDR5 is colored according to the average predicted local distance difference test (pLDDT) score of each residue. The atoms of Arg-14 are shown using green van der Waals spheres. The region between the two folded domains is zoomed in to show interprotein contacts using a ball-and-stick model with cyan, red, and blue spheres representing carbon, oxygen, and nitrogen atoms, respectively. A few interacting residues are annotated. (b) The top and side views of the AlphaFold 3 model of a WDR5 tetramer. The arrow indicates the side from which the view is taken. (c) The sequence alignment using ClustalW for the Win motif residues from the IDR of human WDR5, MLL/SET1 family members, and histone H3. The conserved residues are highlighted in red, while the conservative substitutions are depicted in blue. (d) The number of contacts formed by the AR motif of the IDR tail of one monomer with the Win site of the other monomer during an all-atom MD simulation of the dimer. The black lines show a 10 ns running average of the instantaneous data. (e) BLI sensorgrams for the WDR5-WDR5 interaction. (f) BLI sensorgrams for the interaction of the immobilized NT peptide with ΔN-WDR5. (g) The steady-state maximum BLI response for the WDR5-WDR5 interaction. (h) BLI sensorgrams for the ΔN-WDR5−ΔN-WDR5 interaction. In (e–h), each sensorgram was recorded in at least three independent experiments. (i) A cartoon illustrating the self-association of WDR5. (j) Steady-state fluorescence polarization (FP) anisotropy of the NT peptide−ΔN-WDR5 interaction. The NT peptide was labeled with rhodamine at the N terminus. The labeled peptide was titrated against various [ΔN-WDR5] values. Data indicate mean ± s.d. from *n* = 3 independent experiments.

The binding is primarily driven by Arg-14 of the disordered tail entering the Win site, which is about 34 Å Angstroms away from the tail’s base on the other side of the protein (Figure 1a). There are only 14 residues between the tail’s base and Arg-14, which would span approximately 49 Å if fully stretched. However, such an extended configuration is energetically costly and therefore unlikely. Additionally, the tail is rich in polar and charged residues that prefer a hydrated environment and would face a significant energy penalty if they directly contacted the protein surface. Therefore, self-binding would involve substantial structural strain and a high energy cost, making it unlikely.

Notably, the EAARAQ sequence of the IDR tail resembles the conserved Win motif^56^ of the MLL/SET1 family members and histone H3 (Figure 1c). We identified that this IDR fragment of one WDR5 interacts with the Win binding site^55,60^ of another WDR5 through various noncovalent interactions (Table S1). Specifically, Arg-14 of the Win-like motif of the IDR forms multiple hydrogen bonds, as well as ionic and cation–pi interactions with the Win site of another WDR5. Hence, WDR5 can potentially self-associate through the Win-like motif-Win site interaction.

In addition to such specific interactions, the folded domains of WDR5 in the dimer form numerous nonspecific contacts, including salt bridges between aspartic acid and lysine and polar interactions between threonine and lysine (Figure 1a; Supplementary Tables S2 and S3). Using a cutoff distance of 3.5 Å, 12 such contacts are present within the top-scored structure of the dimer. Similar contact interactions between the folded domains were observed in all five AlphaFold 3 models, with the contacts formed by polar and charged residues. Identical interactions are also present in the AlphaFold 3 structure of a WDR5 tetramer, although specific amino acids involved in the contacts differ from those in the dimer (Supplementary Figure S1b; Supplementary Tables S4 and S5). Thus, on average, each folded domain of a WDR5 protein in the tetramer forms 11 contacts with each of its two neighboring proteins. Such nonspecific contacts, in tandem with a specific interaction between the IDR tail and the Win pocket, create the required conditions for observing LLPS.^61,62^

Explicit-solvent molecular dynamics (MD) simulations were used to examine the stability of the top-scored AlphaFold 3 structure of the WDR5 dimer. The dimer was solvated in an aqueous solution containing 150 mM KCl and simulated unrestrained for 1 μs (Methods; Supplementary Figure S1c). During the simulation, the root mean squared deviation of the folded domain Cα atoms remained below 2 Å (Supplementary Figure S1d). Notably, the specific binding of the IDR tail to the Win pocket persisted through the simulations (Figure 1d). Interestingly, in the one-microsecond simulation of the R14N-WDR5 mutant, which started from the AlphaFold 3 structure of the wild-type WDR5, one of the two N-terminal IDR tails was seen to leave the pocket (Supplementary Figure S1e,f), reinforcing our conclusion regarding the critical role of Arg-14 in promoting the self-interaction of WDR5.

To test these findings further, we investigated the self-association of WDR5 using biolayer interferometry (BLI). Here, WDR5, ΔN-WDR5, a deletion variant of WDR5 lacking the N-terminal IDR, or a 23-residue N-terminal tail (NT) peptide (Methods; Table 1; Supplementary Figure S3), were each immobilized onto a BLI sensor surface. At the same time, WDR5 or ΔN-WDR5 was each kept free in the well. The association and dissociation phases of the WDR5-WDR5 interaction were noted by time-dependent enhancements and declines in the BLI response, respectively (Figure 1e). Notably, the association curves do not saturate at higher WDR5 concentrations, [WDR5], more than likely due to an altered bimolecular association process. At the same time, the dissociation phases were faster than the resolution limit of BLI. A similar response was observed when ΔN-WDR5 was immobilized on the sensor surface and WDR5 was kept free in the well (Supplementary Figure S4a). To confirm that this interaction occurred between the N-terminal IDR and the Win site of WDR5, the NT peptide was immobilized onto the BLI sensor surface, and ΔN-WDR5 was kept free in the well. The association and dissociation phases were also noted (Figure 1f). Remarkably, the maximum BLI signals were attained much faster than those with high [WDR5] values against the immobilized WDR5 on the sensor surface (Figure 1e). Using a steady-state BLI analysis, a low affinity of the self-association of WDR5 was determined with an equilibrium dissociation constant (s.e.m.; *K*_D_) of 18.6 ± 3.5 μM (Figure 1g).

**Table 1:**
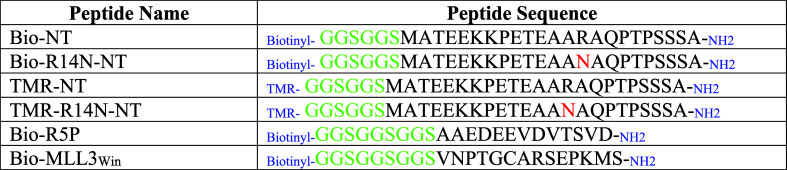
The Peptides Used in This Work^a^

a The C-terminus of all peptides was amidated. The N-termini of the NT, R14N-NT, and R5P peptides were biotinylated. The N-termini of the NT and R14N-NT peptides were also labeled with tetramethyl rhodamine (TMR). The relevant sequences and linkers are marked in black and green, respectively. N-terminal and C-terminal modifications are marked in blue, while the substitution is marked in red.

In addition, no BLI response was found for the ΔN-WDR5−ΔN-WDR5 interaction (Figure 1h), substantiating that the Win-like motif of the IDR is directly involved in the self-association of WDR5. While AlphaFold 3 predicted nonspecific surface-to-surface interactions between the structured regions of two WDR5 molecules (Supplementary Tables S2 and S3), our BLI experiments did not detect such interactions. The critical Arg-14 residue predominantly drives the Win-like motif-Win site interaction.^56^ Hence, we tested an R14N-NT peptide immobilized onto the BLI sensor and found no BLI response with ΔN-WDR5 in the well (Table 1; Supplementary Figure S4b). Further, this finding was confirmed by closely related experiments with R14N-WDR5, a full-length WDR5, whose Arg-14 was replaced by Asn. Hence, we immobilized R14N-WDR5 onto the BLI sensors and tested its binding with WDR5 and R14N-WDR5 (Supplementary Figure S5). The R14N-WDR5–WDR5 interaction showed a similar response to the WDR5–WDR5 interaction (Figure 1e). In contrast, the R14N-WDR5–R14N-WDR5 interaction exhibited a significantly reduced BLI response, confirming that Arg-14 is essential in mediating the WDR5 self-association. These experimental findings agree with the determinations of full-atomistic computational studies presented above. Again, these results validate the specificity of the WDR5-WDR5 interaction, likely leading to the accumulation of WDR5 chain-like networks on the BLI sensor surface (Figure 1i).

Then, fluorescence polarization (FP) anisotropy^63^ was used to study the interaction of the rhodamine-labeled NT peptide with ΔN-WDR5. The fraction bound increased at an elevated ΔN-WDR5 concentration, [ΔN-WDR5] (Figure 1j), with a *K*_D_ (s.e.m.) of 11.5 ± 2.3 μM. In contrast, no interaction was found between R14N-NT and ΔN-WDR5 (Supplementary Figure S6). Hence, we conclude that the Win-like motif of the IDR drives the WDR5 self-association with specificity and low affinity. Notably, employing independent determinations and different approaches (Figure 1g,j), the affinities of WDR5-WDR5 and NT peptide−ΔN-WDR5 interactions were closely similar, suggesting related binding mechanisms in both cases.

To consolidate this conclusion further, we examined the BLI binding curves of the immobilized RbBP5 peptide (R5P)-WDR5 interactions. R5P interacts with WDR5 through the WDR5 binding motif (WBM) site (Table 1).^64^ Again, this interaction showed a nonsaturating regime at increased [WDR5] values (Supplementary Figure S7a). We hypothesized that an additional Win-mediated interaction occurs while R5P binds to the WBM site of WDR5, allowing a chain-like oligomerization process (Supplementary Figure 7b). To test this hypothesis, a high-affinity Win-site peptidomimetic inhibitor (sequence ARTEVY) was employed to block the Win-mediated interaction.^65^ As expected, ARTEVY inhibited the nonsaturation regime of the R5P–WDR5 interaction in a [WDR5]-dependent manner (Supplementary Figure S7c). Similar results were obtained for the R5P−ΔN-WDR5 interaction (Supplementary Figure S7d and Table S6). In addition, ARTEVY only affected the BLI sensorgrams of WDR5 (Supplementary Figure S8a), not those of ΔN-WDR5 (Supplementary Figure 8b). Finally, a Win-like motif-based peptide ligand of mixed lineage leukemia 3 (MLL3_Win_)^66,67^ methyltransferase was utilized to explore its interaction with WDR5 or ΔN-WDR5, and closely similar results were acquired (Table 1; Supplementary Figure S9 and Table S7). These outcomes provide compelling evidence for the direct implication of the N-terminal IDR in the self-association of WDR5 when the Win site is exposed.

### Confirmatory Tests for the Chain-Like Oligomerization of WDR5

Next, we performed a dynamic light scattering (DLS) study to illuminate the distribution of various WDR5 assemblies in the solution. Initially, we tested the bovine serum albumin, confirming its hydrodynamic radius of 4.2 ± 0.6 nm (Supplementary Figure S10).^68^ At 25 μM, ΔN-WDR5 was monodisperse (Supplementary Figure S11a). The hydrodynamic radius was 1.8 ± 0.2 nm, indicating its monomer nature (Supplementary Figure S11b). In contrast, 25 μM WDR5 showed a polydisperse distribution with hydrodynamic radii between 1.9 and 60 nm, corresponding to the monomeric and oligomeric species (Supplementary Figure S11c,d).

### WDR5 Undergoes Phase Separation under Physiological Conditions

Given that WDR5 self-associates at low micromolar concentrations, we examined whether this hub drives phase separation in a cell-free environment. Here, mVenus,^69^ a monomeric variant of yellow fluorescent protein, was fused to the C-terminus of WDR5. This fusion protein was purified and spectroscopically characterized (Supplementary Figure S12). AlphaFold 2 ^70,71^ predictions for WDR5-mVenus indicated that the fusion to mVenus does not perturb the structure of WDR5, as evidenced by the pLDDT and PAE scores (Supplementary Figure S13a,b). The N-terminal IDR and the folded domain adopted typical conformations^57,58^ within the fusion protein complex (Supplementary Figure S13c).

WDR5-mVenus was diluted into the phase separation buffer containing 10% (w/v) 8 kDa-molecular weight poly­(ethylene glycol) (PEG-8k), a nonionic osmolyte (Methods). This hyperosmotic condition corresponded to an osmolarity of 377 ± 6 mOsmol/L (Supplementary Table S8), mimicking the densely packed milieu of the cell. Interestingly, we found that WDR5 forms homogeneous droplets in solution (Figure 2a). The number and size of these droplets increased at elevated [WDR5] values (Figure 2b). They also exhibited a condensed phase composed of a WDR5-compact domain separated from the dilute-surrounding solution. In contrast, ΔN-WDR5-mVenus and mVenus did not undergo phase separation at the tested concentrations (Supplementary Figure S14). Next, we evaluated the partition coefficient, *P*, of WDR5 within the droplet. *P* is the ratio of the average intensity within the condensate, *I*_in_, to the average intensity within the surrounding medium, *I*_out_. The intensity profile was extracted from a confocal cross-sectional analysis of the droplet (Figure 2c). In addition, the turbidities of the WDR5-mVenus solutions in the presence of various crowding agents were explored. Except for PEG-0.4k and glycerol, these osmolytes produced a turbid WDR5-mVenus solution in a concentration-dependent manner (Figure 2d), likely due to distinctions in the local osmotic pressure made by various crowding agents. The WDR5 droplets exhibited essential features of phase-separated condensates, including a high sphericity (Figure 2e). Moreover, they showed typical fusion behavior with an average fusion time of ∼3.1s (Figure 2f). To rule out that mVenus is not involved in droplet formation, we analyzed unlabeled WDR5 by diluting it into the phase separation buffer utilizing transmitted light microscopy. Remarkably, we also observed the formation of liquid droplets by unlabeled WDR5 (Supplementary Figure S15a). Based on steady-state FP anisotropy measurements (Figure 1j; Supplementary Figure S6), we were stimulated to test whether Arg-14 of the IDR is a central player in the droplet generation.

**Figure 2:**
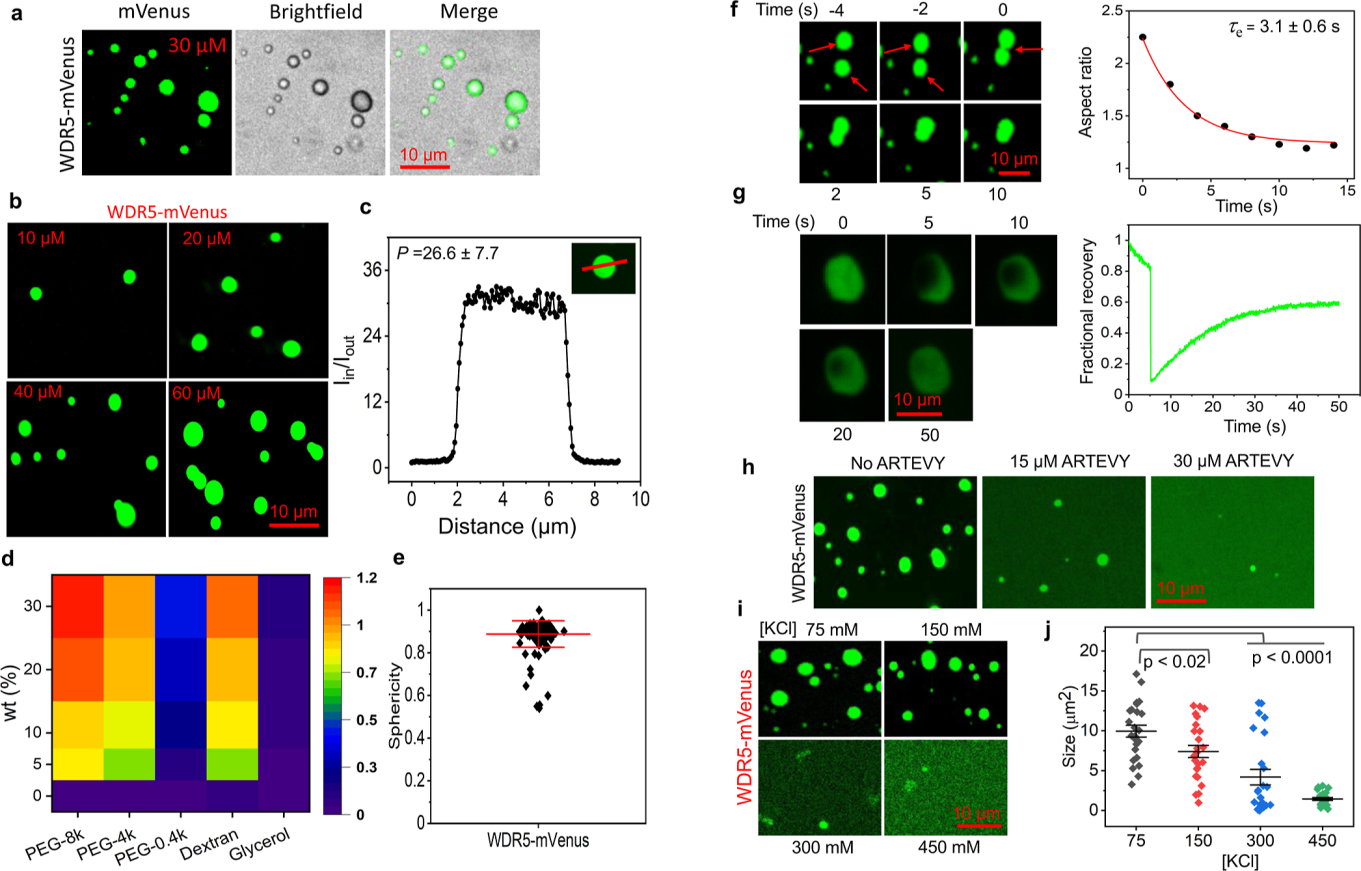
WDR5 forms liquid droplets in a cell-free environment. (a) Images of WDR5-induced phase separations were recorded at mVenus and brightfield channels. The concentration of WDR5-mVenus was 30 μM. The experiment was independently repeated *n* = 4 times with similar results. (b) Images of WDR5-induced phase separations were recorded at various concentrations of WDR5-mVenus. (c) The partition coefficient, *P*, is determined by analyzing the representative intensity profiles derived from a selected confocal cross-section that traverses the core of a droplet (inset) at 20 μM WDR5-mVenus. Intensity values were normalized to the background level (mean ± s.d., *n* = 29 droplets). (d) Representative heatmap of turbidity (OD_600_) at various concentrations of crowding agents. Thirty μM WDR5-mVenus was used. This experiment was independently repeated *n* = 3 times with similar results. (e) The sphericity of WDR5 droplets at 20 μM concentration using ImageJ (Methods; mean ± s.d., *n* = 179 droplets). (f) On the left side, the plot shows the time dependence of the image sequence of merging droplets. On the right, the time dependence of the aspect ratio for the displayed merging event shows exponential decay. The fusion time, τ_e_, is shown as mean ± s.e.m. This experiment has been independently repeated *n* = 3 times with similar results. (g) A sequence of FRAP images with a droplet before bleach (*t* = 0 s), at bleach (*t* = 5 s), and the recovery (*t* = 6–50 s) (left). Quantification of the FRAP signal normalized to the maximum intensity (right). (h) The effect of ARTEVY, a Win site inhibitor, on the droplet formation. (i) The effect of various [KCl] values on the droplet formation at a concentration of 30 μM WDR5. This experiment was independently repeated *n* = 3 times with similar results. (j) The droplet size was determined at various [KCl] values (mean ± s.d., *n* = 24 droplets). Here, the phase-separation buffer contained 10% (w/v) PEG-8k. In all imaging panels, the horizontal scale bar was 10 μm.

Thus, we performed the same experiment with the R14N-WDR5 mutant. In agreement with our expectation, no droplet was detected under similar conditions and within the same concentration range (Supplementary Figure S15b). R14N-WDR5 only showed the droplets at a high concentration regime, which suggests that R14N-WDR5 requires an elevated concentration to undergo phase separation, likely due to a lack of Arg-14-dependent weak interaction. In addition, we examined whether changes in salt concentration influence droplet formation in the case of R14N-WDR5. Our results showed that R14N-WDR5 droplets maintained a similar size at 100 mM and 200 mM KCl. However, at 400 mM KCl, we observed a slight reduction in droplet size (Supplementary Figure S16). Further, we assessed the unlabeled ΔN-WDR5; no liquid-like droplets were detected in this case.

Therefore, we conclude that the Win-like motif-Win site interaction drives the phase separation of WDR5. Such site-specific homotypic interactions have been observed in other proteins, such as nucleophosmin 1 (NPM1 ^72^ and Ras-GTPase-activating protein binding protein 1 (G3BP1),^6,73^ which feature self-oligomerization domains that drive phase separation.

Using fluorescence recovery after photobleaching (FRAP), we examined the dynamics of fluidity within the WDR5 condensate.^74^ A confined region of the droplet was photobleached, followed by subsequent monitoring of the fluorescence recovery (Figure 2g, left). A substantial resurgence in the intensity of the targeted region was noted, attaining ∼66% restoration over a 50 s interval and with a half-life of 17.3 s (Figure 2g, right). This data indicates the liquid nature of the WDR5 droplet. Next, we examined the effect of ARTEVY on the droplet stability. ARTEVY showed a concentration-dependent disruption of these droplets (Figure 2h), providing additional evidence that the Win-like motif-Win site interaction mediates them.

Hexanediol was used to substantiate the reversibility of the WDR5 phase separation in a cell-free environment.^75^ WDR5-mVenus-forming droplets were incubated into 10% (v/v) hexanediol, and their disassembly was confirmed (Supplementary Figure S17). The droplets underwent a transition to smear-like shapes. In addition, we hypothesized that electrostatic interactions facilitate these liquid droplets through Arg-14 of the IDR and the acidic Win site (Supplementary Figure S18a). As anticipated, we observed a disruption of the liquid droplets (Figure 2i) and a decrease in their size at elevated KCl concentrations (Figure 2j). This confirms that the WDR5-mVenus phase separation is at least in part mediated by electrostatic interactions that stabilize self-association. The rhodamine-labeled NT peptide was also incubated in the phase separation buffer, and images were acquired. No droplets were noted, even at a very high NT peptide concentration (Supplementary Figure S18b), validating the direct participation of the Win-like motif–Win site interaction in generating the condensate.

In addition, we evaluated WDR5 condensation at various temperatures. The droplets were spherical at 25 and 37 °C (Supplementary Figure S19 and Table S9). Surprisingly, at 10 °C, they underwent an aspherical shape and showed adhesion, resulting in structures that deviated from the liquid phase. This process indicates nucleation and growth mechanisms within the condensates and incomplete merging events among multiple droplets. Intriguingly, an extended incubation at 25 °C also led to the merging of various droplets (Supplementary Figure S20a) with perturbed sphericity (Supplementary Figure S20b), leading to gel-like structures (Supplementary Figure S21). This data suggests a substantial temperature decrease and longer incubation times catalyze a transition from liquid to gel-like condensates. WDR5 has a seven-bladed, WD-40 repeat-based β-propeller structure. Each blade is made up of four large antiparallel β-strands. One current theory is that this structural composition may involve molecular rearrangements within the condensate, especially the strengthening of intermolecular interactions, such as the formation of extensive hydrogen-bonded β-sheet networks.^76^ These rearrangements cause a transition in material state from liquid-like to gel-like.

### Osmotically Induced Nuclear Condensation of WDR5 in Mammalian Cells

In nonstressed HeLa cells, the endogenous WDR5 displayed a diffuse immunostaining pattern within the nucleus (Figure 3a, top). However, the endogenous WDR5 underwent a substantial reorganization in the sorbitol-induced hyperosmotic stress as its concentration within punctate structures (Figure 3a, bottom). Osmotically stressed cells^77^ exhibited many WDR5 nuclear puncta (NP), showing a significantly higher accumulation of WDR5 than in unstressed cells (Figure 3b,c). These observations on endogenous WDR5 were recapitulated with HEK-293T cells (Supplementary Figure S22). To test if these LLPS observations are replicated in exogenously expressed cells, HeLa cells were transiently transfected with WDR5-mVenus and ΔN-WDR5-mVenus (Supplementary Figure S23a). Without osmotic stress, the exogenously expressed WDR5-mVenus diffused across the nucleus (Supplementary Figure S23b). In 300 mM sorbitol, only WDR5-expressing cells formed NP (Figure 3d), but not the ΔN-WDR5, its truncated variant (Supplementary Figures S23c and S24a).

**Figure 3:**
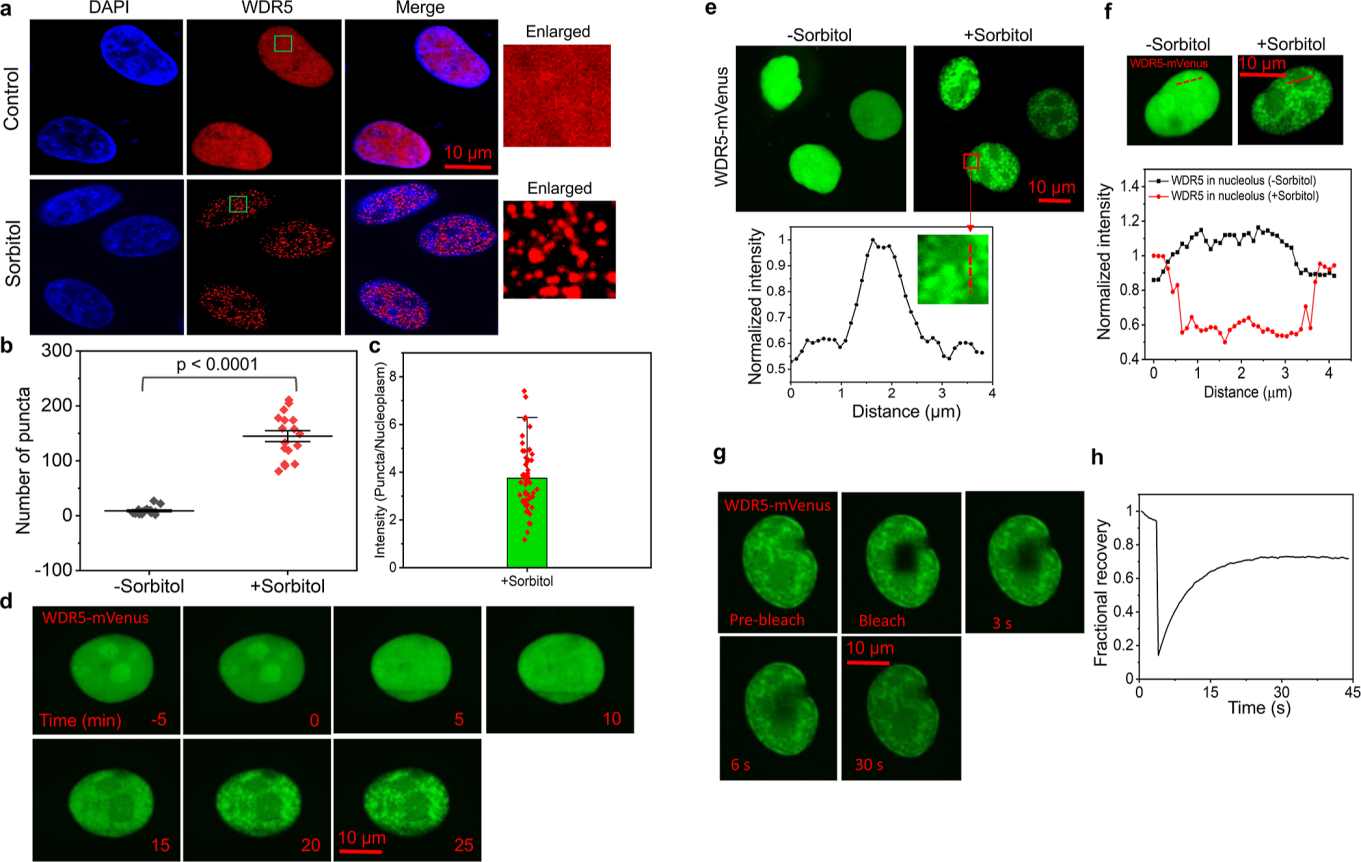
WDR5 undergoes LLPS in living cells. (a) The immunostaining of the endogenous WDR5 in HeLa cells shows that WDR5 is diffused in the nucleus (top) but forms NP under hyperosmotic conditions (bottom). The cells were incubated in 300 mM sorbitol for 25 min. Magnification of boxed regions in treated and nontreated cells to visualize NP. (b) A significant amplification of the number of NP with sorbitol under the experimental conditions from (a) (mean ± s.d., number of cells, *n* = 17). (c) The ratio of [WDR5] in NP to nucleoplasm (mean ± s.d., *n* = 54). (d) Time-lapse images of WDR5-mVenus-expressing HeLa cells were recorded in 300 mM sorbitol. (e) Images of HeLa cells expressing WDR5-mVenus were recorded without sorbitol and with 300 mM sorbitol after 20 min (top). The line scan across the NP represents the accumulation of WDR5 in the specified region (bottom). Intensity was normalized to the maximum value. Magnification of the boxed area in sorbitol-incubated cells indicates the NP (inset). (f) A single HeLa cell nucleus under sorbitol-free and sorbitol-incubated conditions (top) and the experimental conditions from (e). The fluorescence intensity was measured across the nucleolus (bottom). The fluorescence intensity was normalized to the maximum value. (g) FRAP images of sorbitol-incubated HeLa cells were recorded at various time points. The cells were incubated in 300 mM sorbitol for 20 min. (h) A representative FRAP recovery curve of WDR5 condensates. The experiment has been independently repeated for *n* = 5 times with similar results. In all imaging panels, the horizontal scale bar was 10 μm.

In those NP, we observed a ∼2-fold higher accumulation of WDR5 with respect to nucleoplasm (Figure 3e; Supplementary Figure S24b). No NP or higher accumulation in specific areas was found in HeLa cells expressing only mVenus under the sorbitol-induced hyperosmotic condition (Supplementary Figure S24c). Surprisingly, WDR5, which exhibited an amplified accumulation in the nucleoli under sorbitol-free conditions, diffused outside the nucleoli and formed punctate structures (Figure 3f), supporting the redistribution of WDR5 within the nucleus. In addition, we examined the diffusion pattern of WDR5 engaged in phase separation using FRAP. In osmotically stressed cells, when bleaching WDR5-mVenus, fluorescence quickly recovered (e.g., 30 s) with a half-life of ∼2.5 s and an overall recovery of ∼72% (Figure 3g,h). This finding is consistent with a liquid-like behavior. On the other hand, the fluorescence recovery was much lower in unstressed cells, with a half-life of ∼2.1 s and a recovery of ∼44% (Supplementary Figure S25). Therefore, WDR5 undergoes a nuclear phase separation in membraneless organelles under hyperosmotic conditions. There is a tendency for WDR5 to phase separate and accumulate in NP in stressed cells, suggesting that this phenomenon is a potential mechanism for finely controlling local gene expression.

Although tumor microenvironments may exhibit modest increases in osmolarity (320–340 mOsm; approximately 10–15%), these levels generally do not cause the distinct phase separation observed with high sorbitol treatment. To our knowledge, WDR5 nuclear condensates have not been reported in cancer cells or in physiologically hypertonic tissues, such as the renal medulla, under normal conditions. However, cells chronically exposed to elevated osmotic conditions, like renal medullary cells, could offer an interesting physiological context to explore whether sustained hyperosmolarity affects WDR5 condensation. We see this as a promising area for future research.

### MYC is Incorporated into WDR5 Condensates

MYC binds to regulatory sequences, modulating the expression of many target genes. WDR5 plays a central role in this process, facilitating the recruitment of MYC to the enhancer sites of chromatin.^27^ Therefore, we asked whether MYC can form droplets in vitro, with and without WDR5, its transcription cofactor. Recombinant MYC-mScarlet-I fusion protein was purified and characterized (Supplementary Figure S26). Here, mScarlet-I is a bright monomeric red fluorescence protein.^78^ Then, MYC-mScarlet-I was added to the phase separation buffer containing 10% (w/v) PEG-8k. Fluorescence microscopy of the mixture revealed that MYC forms homogeneous droplets at two tested concentrations (Figure 4a,b). Further, MYC droplets showed a typical fusion and fission behavior (Supplementary Figure S27a,b). The FRAP analysis of these droplets indicated a half-life of ∼5.3 s, which is ∼2.5-fold longer than WDR5 droplets and a recovery of ∼69% (Supplementary Figure S27c). When MYC-mScarlet-I and WDR5-mVenus were mixed, heterotypic droplets containing WDR5 and MYC were noted (Figure 4c,d).

**Figure 4:**
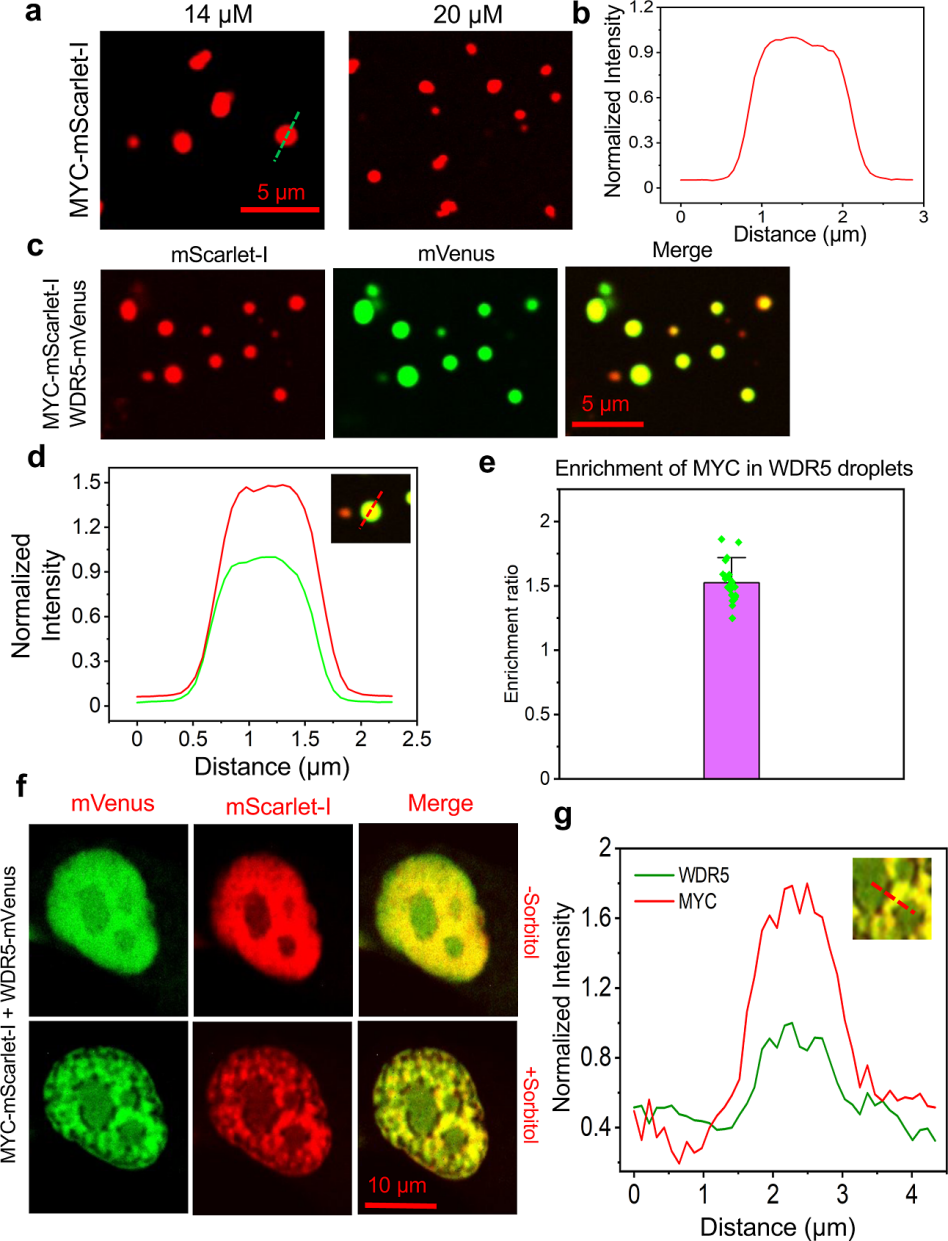
MYC is incorporated in WDR5 condensates. (a) Images of phase-separated WDR5 binding partner MYC at 14 μM and 20 μM concentrations. (b) The intensity profile of a confocal slice through a droplet center shows the MYC distribution. (c) Images show that MYC was recruited into WDR5 droplets. Twenty μM of each of MYC-mScarlet-I and WDR5-mVenus were added to the phase separation buffer. In (a,c), the scale bar is 5 μm. This experiment was independently repeated *n* = 3 times with similar results. (d) An intensity curve of a droplet containing WDR5 and MYC. Red and green denote MYC and WDR5, respectively. (e) The enrichment ratio of MYC-mScarlet-I in WDR5-mVenus droplets (*n* = 22). (f) Live-cell imaging showing the colocalization of WDR5 and MYC within NP of HeLa cells exposed to 300 mM sorbitol-induced hyperosmotic stress. The scale bar is 10 μm. This experiment was independently repeated for *n* = 5 times with similar results. (g) Intensity profiles of MYC-mScarlet-I (red) and WDR5-mVenus (green) in coexpressing HeLa cells. This experiment was independently repeated for *n* = 15, where *n* is the number of cells.

The enrichment of MYC in these droplets was ∼1.5-fold higher than that of WDR5 (Figure 4e). Finally, we explored how the coexpression of MYC and WDR5 behaves in living cells under hyperosmotic conditions. Hence, HeLa cells were cotransfected with MYC-mScarlet-I and WDR5-mVenus and imaged at the respective channels. In 300 mM sorbitol-induced hyperosmotic conditions, WDR5 and MYC formed droplet-like punctate structures (Figure 4f). Overlapping signals were noted in those condensates (Figure 4g). These results suggest that WDR5, which interacts with MYC,^27,34,39,40,79^ can impact targeted gene expression under adverse conditions through LLPS. To examine whether the MYC–WDR5 interaction contributes to MYC localization within WDR5 droplets, we used an inhibitory peptide (EEEIDVVSV)^28^ that mimics the MYC region responsible for binding WDR5. This competitive peptide was applied at a relatively high concentration of 30 μM to effectively disrupt the MYC–WDR5 interaction. Interestingly, upon this treatment, the accumulation of MYC within the condensates was markedly reduced (Supplementary Figure S28). This observation suggests that this interaction plays a key role in mediating the MYC sequestration into these condensates.

### Amyotrophic Lateral Sclerosis-Linked Proteins Colocalize with WDR5 in NP under Hyperosmotic Stress

Fused in sarcoma (FUS) and TAR DNA-binding protein-43 (TDP-43), key RBPs implicated in amyotrophic lateral sclerosis (ALS), have been reported to accumulate in nuclear granules under high salt conditions.^80^ These prion-like domain (PrLD)-containing proteins are also among the most studied RBPs that undergo LLPS in health and disease.^81^ To explore whether sorbitol-induced hyperosmotic stress promotes the phase separation of ALS-related proteins and their accumulation in NP, we performed immunofluorescence staining to label endogenous WDR5, FUS, and TDP-43 in HeLa cells. We examined the potential colocalization of WDR5 NP with FUS and TDP-43 in 300 mM sorbitol-induced hyperosmotic stress. Co-immunostaining for WDR5 and FUS, as well as WDR5 and TDP-43, revealed that both FUS and TDP-43 were present in WDR5 NP in response to sorbitol stress (Supplementary Figure S29a,b) and colocalized with WDR5 (Supplementary Figure S29c,d). These findings suggest that FUS and TDP-43 may play a regulatory role in the assembly or function of WDR5-associated NP during hyperosmotic stress.

### RNA Regulates the WDR5 Condensation Both In Vitro and in Living Cells

Adding RNA to RBPs can significantly influence their phase separation behavior.^10,19,82^ To investigate whether RNA regulates the phase separation of WDR5, we performed an in vitro droplet formation assay, in which the binding fragment of HOXA transcript at the distal tip long non-coding RNA (HOTTIP lncRNA)^38^ was added to a WDR5-containing phase-separating mixture. For the sake of simplicity, we will denote this binding fragment as RNA hereafter. Remarkably, we found that RNA was sequestered in WDR5 droplets in a concentration-dependent manner (Figures 5a,b and S30). Next, we confirmed that the RNA accumulation into F266A-WDR5 droplets was markedly diminished compared to WDR5 because of this mutant’s significantly reduced RNA-binding affinity^38^ (Figure 5c,d).

**Figure 5:**
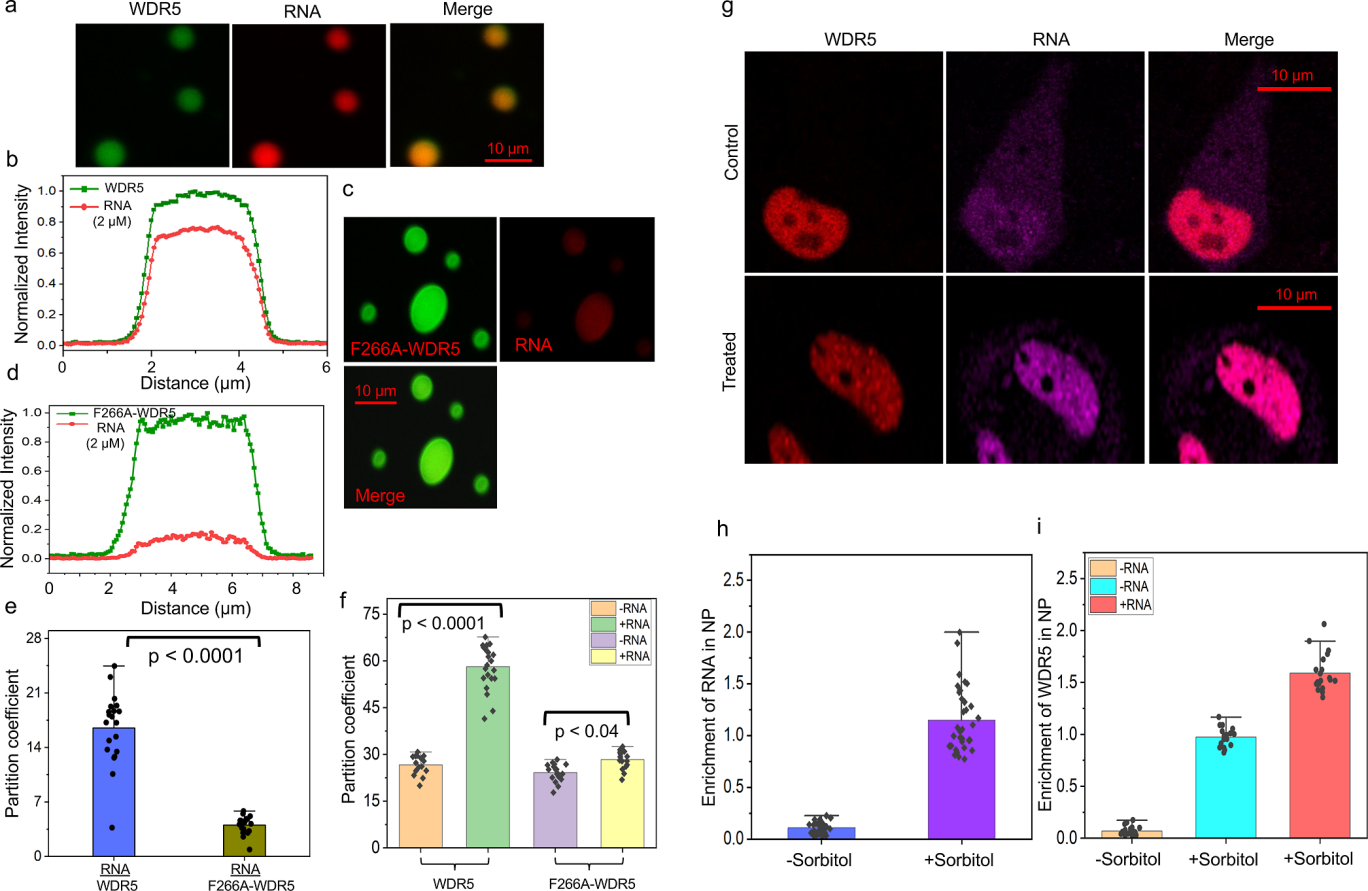
Sequestration of RNA in WDR5 droplets and NP in HeLa cells. (a) Images of 10 μM phase-separated WDR5 and 2 μM Alexa Fluor 568 (AF568)-labeled RNA. Images show that RNA was significantly accumulated into the WDR5 condensates. The horizontal scale bar is 10 μm. (b) A representative confocal slice’s intensity profile through the droplet’s center was plotted for WDR5 + RNA. Red and green denote RNA and WDR5, respectively. (c) Images of 10 μM phase-separated F266A-WDR5 and 2 μM AF568-labeled RNA. Images show that the accumulation of RNA was much lower in the F266A-WDR5 condensates than in the WDR5 condensates. The horizontal scale bar is 10 μm. (d) A representative confocal slice’s intensity profile through the droplet’s center was plotted for 10 μM F266A-WDR5 + 2 μM RNA. This experiment was independently repeated *n* = 4 times with similar results. (e) Partition coefficients of fluorescently labeled RNA with WDR5 and F266A-WDR5. RNA + WDR5 (*n* = 20) and RNA + F266A-WDR5 (*n* = 22), where *n* represents the number of distinct droplets from three biological replicates. (f) The partition coefficient of mVenus-labeled WDR5 and Alexa Fluor 488 (AF488)-labeled F266A-WDR5 with and without RNA. In this experiment, we measured the partition coefficient of wild-type and mutant WDR5 as a droplet versus the dilute phase intensity ratio. WDR5 without RNA (*n* = 15), WDR5 with RNA (*n* = 22), F266A-WDR5 without RNA (*n* = 18), and F266A-WDR5 with RNA (*n* = 16). In e and f, RNA and protein concentrations are indicated in a and c. A two-tailed unpaired *t*-test was used in e and f. (g) WDR5 phase separation was induced by incubating the cells in 300 mM sorbitol for 40 min. Immunofluorescence staining of WDR5 and RNA shows the enrichment of RNA in NP. Images are representative examples from *n* = 3 independent experiments. The horizontal scale bar is 10 μm. (h) The panel shows a plot of the enrichment of RNA in NP without and with sorbitol using *n* = 27 cells and *n* = 34 cells, respectively, from *n* = 3 independent experiments. (i) The panel shows a plot of the enrichment of WDR5 in sorbitol-stimulated NP in the absence and presence of RNA using *n* = 18 cells and *n* = 20 cells, respectively, from *n* = 3 independent experiments.

Furthermore, we assessed RNA partitioning in wild-type and mutant WDR5 condensates. To determine the extent to which RNA was sequestered into WDR5 and F266-WDR5 droplets, we synthesized an RNA with a 5′-attached Alexa Fluor 568 (AF568) tag. Upon adding RNA, fluorescent RNA-containing droplets appeared within minutes and enriched into liquid-like droplets. We measured the RNA partition coefficient between WDR5 and F266A-WDR5 liquid-like droplets across all sizable droplets. This analysis confirmed that RNA partitions at a higher concentration in WDR5 droplets than in F266A-WDR5 droplets (Figure 5e, Supplementary Table S10). Remarkably, we found that RNA significantly increased WDR5 partitioning into droplets relative to the surrounding dilute phase and RNA-free WDR5 droplets (Figure 5f, Supplementary Table S11). In contrast, this effect was much less pronounced for F266A-WDR5. Our findings directly support that RNA plays a crucial role in regulating the assembly of WDR5 liquid-like droplets. Next, we asked if the RNA is recruited into the WDR5 NP and modulates its phase separation in cells. Hence, we transfected HeLa cells with RNA oligos and assessed the distribution of endogenous WDR5 by immunofluorescence staining. Consistent with our in vitro results, RNA was recruited to the WDR5-containing NP in HeLa cells (Figure 5g,h). Finally, RNA enhanced WDR5 sequestration in NP under hyperosmotic conditions (Figure 5i).

### Functional Impact of RNA Association on WDR5 Activity

As a direct interactor of histone H3 lysine K4 (H3K4), WDR5 is potentially a transcriptional activator.^83,84^ Next, we conducted a luciferase reporter assay to determine gene activation. In this way, we examined the functional implications of the physical association between RNA and WDR5.^85^ The luciferase reporter assay is widely used in cell biology to determine whether a transcriptional regulator influences target gene expression.^37,86,87^ Interestingly, F266A-WDR5 showed a drastically diminished ability to activate the luciferase reporter gene compared to WDR5, as evidenced by the low luminescence signal (Methods; Supplementary Figure S31). This finding agrees well with this mutant’s significantly reduced RNA-binding affinity.^38^ This result suggests that the absence of WDR5 interaction with the lncRNA binding fragment significantly impairs its ability to activate gene expression in the reporter assay. Hence, this outcome illuminates the role of RNA participation in regulating WDR5 function and its phase separation behavior. In simplified reasoning, if the association of lncRNA with WDR5 in its diffuse state upregulates gene expression, as shown previously,^38^ then its accumulation along with RNA in NP would more than likely further enhance the expression of WDR5 target genes. Our study also establishes the phase-separation profiles of MYC and ALS proteins and their colocalization with WDR5 in NP. However, it does not show direct evidence of the functional implications of this coexistence. This warrants future explorations of WDR5-mediated LLPS in mammalian cells.

## Conclusions

In summary, we utilize full-atomistic MD simulations, a deep learning-based system, protein engineering, and an array of biophysical, biochemical, and imaging approaches to identify a unique molecular mechanism driving the LLPS of WDR5 in cell-free environments and living cells. The WDR5 self-association nucleates LLPS through a site-specific interaction between its relatively short N-terminal IDR and a multitasking Win pocket. Moreover, we demonstrate the formation of heterotypic condensates containing WDR5, RNA, and other binding proteins. A network of specific and nonspecific protein–protein and protein-RNA interactions also mediates these multicomponent condensates. Notably, the condensate formation in mammalian cells induces a substantial nuclear redistribution of WDR5, especially in nucleolar regions. A relatively small accumulation of MYC in the WDR5-driven condensates aligns with a weak–affinity interaction between MYC and WDR5.^27,79^ Our findings reveal that RNA modulates the phase separation profile of WDR5 and can stimulate the formation of WDR5 NP. In this case, RNA amplifies the partitioning of WDR5 into the phase-separated condensates through a recruitment pathway. This study provides compelling evidence for colocalization of the most well-studied PrLD-containing RBPs, FUS and TDP-43, with WDR5 in NP under hyperosmotic conditions. Further, luminescence data suggest that WDR5 can locally regulate protein expression, highlighting its functional importance in cellular stress adaptation. Previous studies have shown that, under stress conditions, phase-separated nuclear bodies protect accumulated proteins from the detrimental effects of stress.^88,89^ This suggests that phase separation via the WDR5-RNA interaction may serve as a general stress response mechanism to protect essential biomacromolecules and regulate local gene expression. These outcomes unravel the complex mechanisms governing heterotypic nuclear LLPS and the interplay between WDR5, MYC, and RNA in driving these multicomponent dynamic processes.

## Methods

### Proteins Examined in This Study

The amino acid sequences of WDR5 and MYC were extracted from Uniprot (WDR5-P61964; MYC-P01106). For the sake of simplicity, the full-length WDR5 (or wild-type WDR5) and N-terminus truncated WDR5, i.e., WDR5^23–334^, were named WDR5 and ΔN-WDR5, respectively. To assess the folding of the structured domains, the predicted WDR5 and R14N-WDR5 mutant conformations were compared with the crystal structure 4Y7R.^27^

### All-Atom Molecular Dynamics (MD) Simulations

Unless specified otherwise, all-atom MD simulations were performed using the classical MD package NAMD2 ^90^ under periodic boundary conditions and a 2 fs integration time step. The CHARMM36 force field^91^ was used to describe proteins, the TIP3P water model,^92^ and the compatible model of ions.^93^ The RATTLE^94^ and SETTLE^95^ algorithms were applied to covalent bonds involving hydrogen atoms in proteins and water molecules, respectively. The particle mesh Ewald (PME)^96^ algorithm was used to evaluate long-range electrostatic interactions over a 1 Å-spaced grid. van der Waals interactions were assessed using a smooth 12 Å-cutoff distance. Langevin dynamics maintained the temperature at 295 K. Wherever a constant number of atoms, pressure, and temperature (*NPT*) ensemble was used, the pressure was maintained at 1 atm using the Nose–Hoover Langevin piston pressure control^97^ by adjusting the system’s dimensions. In all simulations involving harmonic restraints, the spring force constant was set to 1 kcal/(mol Å^2^). The AlphaFold 3^54^ server was used to generate the initial atomic structures of WDR5 assemblies. The program was supplied with the protein sequence and the information that either two or four such proteins exist in the system. The AlphaFold 3^54^ structure predictions were performed for both WDR5 and R14N-WDR5. Each AlphaFold 3 calculation produced five structures ranked according to their atoms’ average predicted Local Distance Difference Test (pLDDT) score.

For our all-atom MD simulations of the WDR5 dimers, we solvated the top-ranked structure of the WT dimer with 150 mM KCl, producing a system with sizes 12 nm × 12 nm × 15 nm and containing 206,568 atoms. Another variant of that system was built by introducing the R14N mutation within the AlphaFold 3 structure of the WDR5 dimer with 206,560 atoms. Both systems were minimized for 5000 steps and then simulated in the *NPT* ensemble for 5 ns, having the C_α_ atoms of the protein harmonically restrained to their initial coordinates. After 5 ns, all restraints were released, and the systems were simulated in the *NPT* ensemble for 1 μs.

### MD Simulations for Generating Docking Configurations

Docking studies were performed using a high-ambiguity-driven protein–protein docking (HADDOCK) server.^59^ Thirty-three configurations were generated based on the protocol prescribed in the HADDOCK guide.^98^ The 1-through-29 residues of the WDR5 protein were first arranged to have a secondary structure of an α helix, a polyproline-II helix, and an unstructured peptide (Supplementary Figure S2a). The three configurations are immersed in a 150 mM KCl solution, a system of sizes 12 nm × 12 nm × 12 nm containing 166,959 atoms. The systems were first minimized for 5000 steps, equilibrated for 3 ns in the constant volume and temperature ensemble with the C_α_ atoms harmonically restrained, followed by a 3 ns restrained NPT simulation. Finally, the restraints were released, and the systems were simulated for 200 ns each. In each simulation, the peptide conformation was considerably diverted from its initial configuration (Supplementary Figure S2b).

### Dihedral Principal Component Analysis

The three 200 ns trajectories were combined for statistical analysis of the microscopic configurations. A set of dihedral angles represented each configuration. The dihedral angles were then transformed to their sine and cosine representations. The resulting data set was subjected to a covariance analysis to reduce the dimensionality of the configurational space. The first two principal axes accounted for ∼50% of the variance (Supplementary Figure S2c). Thus, we projected the configuration space onto these two axes and used 900 bins to divide the phase space. Thirty bins with the highest number of configurations were selected, and the first configuration within each was chosen for the docking calculations.

### Docking Calculations

A total of 33 structures, which included the 30 highest probable conformations and the three initial conformations (α helix, polyproline II, and unstructured), were used for docking calculations. We docked the peptide conformation into the WDR5 protein by specifying the docking pocket using the residues 49, 52, 65, 89, 107, 108, 130, 131, 149, 150, 170, 172, 173, 177, 191, 192, 216, 234, 259, 260, 279, 321. The docking calculations produced a range of docked poses for each configuration with varying scores. The top-scored conformation is shown in Supplementary Figure S2d. Analyses of the MD trajectories were carried out utilizing the MDAnalysis package.^99,100^ The plots were produced using Matplotlib.^101^

### Cloning and Generation of Expression Constructs

A bacterial codon-optimized version of the WDR5-mVenus and MYC-mScarlet-I was synthesized by Integrated DNA Technology (IDT, Coralville, IA). The cDNAs of WDR5-mVenus, ΔN-WDR5-mVenus, MYC-mScarlet-I, and mVenus were amplified and cloned in pET-28a at *NheI* and *Hin*dIII sites. The cDNAs of WDR5 and ΔN-WDR5 were cloned in the pET3a vector, which yielded pET3a-WDR5 and pET3a-ΔN-WDR5, respectively. R14N-WDR5 and F266A-WDR5 were created in pET3a-WDR5 using site-directed mutagenesis (New England Biolabs, Ipswich, MA). For the expression in the mammalian system, the cDNA of WDR5 and ΔN-WDR5 were cloned in the pmVenus-N1 (Addgene #27793) at *NheI* and *AgeI* sites. The cDNA of MYC was cloned in pLifeAct-mScarlet–I–N1 (Addgene #85054) at *NheI* and *Bam*HI sites, in which LifeAct was replaced by our targeted gene. All these cDNAs were genetically fused to the fluorescent proteins using a (GGS)_2_ linker. The cDNAs of these proteins were amplified using a set of primers (Supplementary Table S12). DNA sequencing confirmed all cloning and mutagenesis work (MCLAB, San Francisco, CA). pmVenus-C1 (Addgene plasmid #27794) and pmVenus-N1­(Addgene #27793) were a gift from Steven Vogel. pmScarlet–I–C1 (Addgene #85044), pLifeAct_mScarlet-I_N1 (Addgene #85054) were kindly gifted by Dorus Gadella.

### Protein Expression and Purification

The purification procedure for WDR5 and ΔN-WDR5 closely followed the method outlined earlier.^102,103^ In brief, the pET3a vector encompassing 6 × His-TEV-WDR5, 6 × His-TEV-ΔN-WDR5, 6 × His-TEV-R14N-WDR5, or 6 × His-TEV-F266A-WDR5 was transformed into competent Rosetta BL21­(DE3) pLysS strain of *E. coli* (Novagen, Cat #71403). Following the transformation, these cells were cultivated overnight on Luria–Bertani (LB) agar plates supplemented with carbenicillin and chloramphenicol at 37 °C. Subsequently, a single colony from these transformations was employed to inoculate a 50 mL starter culture of the Terrific Broth (TB) medium. Incubation of the starter culture occurred overnight at 30 °C. The next day, the starter culture was utilized to inoculate 1 L of TB media. The resulting expression culture was grown at 37 °C until OD_600_ reached 0.5. Then, this was equilibrated at RT for 30 min. Induction was initiated by adding 100 μM IPTG, and the culture was incubated at 16 °C for ∼20 h. Harvested cellular pellets were subjected to lysis through multiple passages using a microfluidizer (model M110L; Microfluidics, Newton, MA). The lysis buffer was 50 mM Tris–HCl, 300 mM KCl, 5 mM EDTA, 2 mM DTT, and pH 7.5, supplemented with 0.5 mM PMSF and an EDTA-free protease inhibitor. The lysate was subsequently centrifuged, and the supernatant was filtered using a 0.22 μm-cutoff filter, then processed through a Ni-NTA column integrated with an NGC Quest 10 Plus Chromatography System (Bio-Rad, Hercules, CA). WDR5 was eluted using an imidazole gradient, and the 6 × His tag was subsequently cleaved utilizing a TEV protease. Then, the Ni-NTA column was again employed to eliminate the 6 × His tag and the TEV protease from the protein solution. mVenus, WDR5-mVenus, and ΔN-WDR5-mVenus were purified similarly, except that 1 mM IPTG was used as an inducer.

To purify MYC-mScarlet-I, *E. coli* BL21 (DE3) cells were transformed with pET-28-MYC-mScarlet-I and induced with 1 mM IPTG. The sedimented cell pellets of 1 L culture were reconstituted in 35 mL of denaturing buffer containing 300 mM KCl, 50 mM Tris–HCl, 20 mM imidazole, 8 M urea, pH 7.5, which was supplemented with cOmplete protease inhibitors (Roche, Indianapolis IN). Subsequently, the resuspended cells were lysed using a microfluidizer (model M110L; Microfluidics). The lysate was centrifuged, and then the supernatant was loaded to a Ni-NTA column on an NGC Quest 10 Plus Chromatography System (Bio-Rad). This was washed with a 10-column volume of the washing buffer (50 mM Tris–HCl, 300 mM KCl, 30 mM imidazole, 8 M urea, and pH 7.5). The elution buffer was 50 mM Tris–HCl, 300 mM KCl, 500 mM imidazole, 8 M urea, and pH 7.5. The protein samples were sequentially dialyzed in three steps. First, they were dialyzed against a buffer containing 150 mM KCl, 50 mM Tris–HCl, 2 mM DTT, 4 M urea, and pH 7.5. Then, they were dialyzed against the same buffer containing 2 M urea. Finally, the dialysis of protein samples was performed against two changes of the buffer, with 10% (v/v) glycerol and without urea. Any remaining precipitates after dialysis were removed through centrifugation at 6000*g* for 10 min. Pure fractions were consolidated and utilized in a cell-free environment for subsequent experiments. MYC-mScarlet-I was further characterized by its excitation and emission being recorded via a SpectraMax i3 plate reader (Molecular Devices, San Jose, CA).

### Peptide Synthesis, Labeling, Purification, and Analysis

The peptides were synthesized and purified by GenScript (Piscataway, NJ; Table 1). Their purity was greater than 95%. All peptides were amidated at the C terminus. For the BLI experiments, the peptides were biotinylated at the N terminus. Conversely, the label-free peptides were acetylated at the N terminus. The peptides were labeled at the N terminus with tetramethyl rhodamine (TMR) for the steady-state fluorescence polarization (FP) assays. GenScript conducted a comprehensive amino acid (AA) analysis and solubility tests to ascertain their quality.

### Biolayer Interferometry (BLI)

The OctetRED384 system (FortéBio, Fremont, CA) was utilized to conduct BLI experiments. Our experimental procedures closely mirrored those detailed in our previous studies.^66^ In this study, WDR5, ΔN-WDR5, or N-terminal tail (NT) peptide (each biotinylated) were immobilized onto streptavidin-coated BLI sensors. Biotin labeling was performed at the N terminus of these ligands. A flexible (GGS)_2_ peptide spacer was introduced to alleviate potential steric hindrance between the biotinylated site and the peptide sequence. For WDR5 and ΔN-WDR5, 11 poly­(ethylene glycol) (PEG) repeats were used for the same purpose. For the association phase, these ligand-immobilized sensors were immersed in wells containing the analyte, while the subsequent shift to analyte-free wells facilitated the recording of the dissociation phase. All the binding curves were rectified by subtracting the response corresponding to the reference well that contained the analyte only. Unless otherwise stated, the running buffer contained 150 mM KCl, 20 mM Tris–HCl, 1 mM TCEP, 1 mg mL^–1^ BSA, and pH 7.5. Further data analysis and curve fittings were achieved by employing the Octet data analysis software (FortéBio). Using global fitting of BLI curves acquired at different analyte concentrations, we extracted the corresponding kinetic rate constants of association and dissociation, *k*_on_ and *k*_off_, respectively. The equilibrium dissociation constant, *K*_D_, was indirectly derived from these kinetic parameters. All reported data and plots resulted from three independent BLI measurements.

### Steady-State Fluorescence Polarization (FP) Measurements

23-residue N-terminal tail (NT) peptide and its mutant R14N-NT were labeled using TMR, employing primary amine chemistry (GenScript; Table 1). These TMR-labeled peptides were introduced into the wells at a final concentration of 20 nM. To evaluate their interactions with ΔN-WDR5, a steady-state fluorescence polarization (FP) anisotropy^104^ assay was conducted in triplicate. The running buffer contained 150 mM KCl, 20 mM Tris–HCl, 1 mM TCEP, and pH 7.5. A serial dilution of ΔN-WDR5 was employed against a fixed concentration of the labeled peptides. This assay used black flat-bottom 96-well Costar assay plates (Corning Inc., Kennebunk, ME). The SpectraMax i3 plate reader (Molecular Devices) was utilized to acquire all steady-state FP measurements. Data was acquired using the SoftMax Pro 6.4 software (Molecular Devices). Measurements were conducted in a light-protected environment. Data were collected at the beginning and after a one-hour incubation at RT. Subsequently, the acquired dose–response data were averaged for fitting using a logistic regression function. This fitting facilitated the determination of the *K*_D_.

### Dynamic Light Scattering (DLS) Measurements

WDR5 and ΔN-WDR5 were filtered to remove the aggregates and then concentrated by centrifugation at 4500*g* at 4 °C using a 3 kDa cutoff Spin-X UF concentrator (Corning). Bovine serum albumin (BSA) was prepared and filtered in the same buffer. This buffer was 20 mM Tris–HCl, 150 mM KCl, 1 mM TCEP, and pH 7.5. Light scattering assays were performed using DynaPro NanoStar II (Wyatt Technology, Santa Barbara, CA). Three independent measurements were made for each protein sample, and the data were analyzed using the DYNAMICS software (Wyatt Technology). Data were fitted using the regularization method.^105^

### Cell Culture and Transfection

HeLa and HEK-293T cells were cultured in six-well plates, coated with collagen or uncoated (Cellvis, Mountain View, CA), at an approximate density of ∼2 × 10^5^ cells per well. The culture environment was kept at 37 °C, 5% CO2, and 70% relative humidity. A PCR test was performed to ensure the absence of mycoplasma contamination. FuGENE-HD (Promega, Madison, WI) or Lipofectamine 3000 (Invitrogen by Thermo Fisher Scientific, Carlsbad, CA) was employed for transfection, which was carried out in serum-free Dulbecco’s Modified Eagle Medium (DMEM). Opti-MEM (Thermo Fisher Scientific) was chosen as the medium for preparing transfection mixtures, which consisted of a transfection reagent and plasmid DNA. In cases of cotransfection, a balanced 1:1 ratio of the two plasmids was employed. To achieve recombinant protein expression levels closely similar to endogenous WDR5 and MYC, plasmid concentrations ranged from approximately 500 to 800 ng per well. The transfection mixture was incubated at RT for 15–20 min before being added to the wells. Cells were incubated with the transfection mixture for ∼6 h. Then, the complete media was added, allowing for the expression of recombinant proteins for approximately 24 h. Subsequently, the cells were washed using Dulbecco’s phosphate-buffered saline (DPBS; Thermo Fisher Scientific) and then replenished with imaging media, DMEM with 25 mM HEPES and no phenol red (Thermo Fisher Scientific) for live-cell imaging.

### Fluorescence Imaging of Phase Separation

To avoid any stimulatory effect of fluorescent protein conjugation on phase separation, monomeric versions of yellow and red fluorescent proteins, mVenus and mScarlet-I,^28^ respectively, were employed for phase separation experiments. Live-cell imaging of HeLa cells was conducted using a spinning-disc confocal microscope, which involved a Yokogawa CSU-W1 50 μm 60 Pinhole system (Nikon, Tokyo, Japan). This was integrated into an inverted Nikon Ti-E microscope with a 100× oil immersion objective (1.4 NA). The experimental setup was enclosed within an incubation chamber (Okolab USA, Ambridge, PA), and an Andor Zyla CMOS camera captured the images. The image acquisition process was managed using the NIS-Elements software (Nikon). The time series data underwent subsequent analysis via ImageJ/FIJI. During live- and fixed-cell imaging, the excitation wavelengths for mVenus and mScarlet-I were 488 and 561 nm, respectively. The emissions of these fluorophores were at the wavelengths of 525 ± 50 nm and 630 ± 75 nm, respectively. mVenus images were pseudocolored and shown as green throughout the article. During live-cell imaging, the exposure time for each fluorophore was 400 ms. The exposure time for LLPS experiments in a cell-free environment was 30 ms. The phase separation buffer contained 20 mM Tris–HCl, 150 mM KCl, 1 mM TCEP, and pH 7.5 with 10% (w/v) PEG-8K. We used the buffers with 20 mM Tris–HCl and 1 mM TCEP for salt analysis experiments and added KCl concentrations, [KCl], 75 mM, 150 mM, and 450 mM. For RNA accumulation and sequestration experiments, the binding fragment of HOTTIP RNA was synthesized and labeled with Alexa Fluor 568 (AF568) by Integrated DNA Technologies (IDT, Coralville, IA). Various concentrations of RNA were added to the phase separating WDR5 mixtures, and confocal imaging was conducted. The purified F266A-WDR5 was labeled with Alexa Fluor 488 (AF488) to facilitate fluorescence droplet imaging. The excitation intensity for AF488 was optimized to achieve an emission profile like that of mVenus. This adjustment was essential to effectively compare fluorescence signals between the dye and mVenus, minimizing discrepancies due to differences in their intrinsic brightness or spectral properties. For experiments about the colocalization analysis, images of HeLa cells coexpressing WDR5-mVenus + MYC-mScarlet-I were captured. The Coloc2 tool was employed for precise colocalization assessment.^106^ Combining object-recognition-based colocalization analysis with pixel–intensity correlation facilitated the derivation of an object-corrected Pearson coefficient.^28^

### Immunofluorescence for Detecting Nuclear Punctum (NP) Proteins

HeLa and HEK-293T cells were fixed with 4% paraformaldehyde (PFA) for 15 min at RT. After removing PFA, cells were rinsed thrice in DPBS for 5 min each. To detect WDR5, anti-WDR5 (1:200) was used as the primary antibody (Cell Signaling Technology, Danvers, MA; Cat #: 13105). To visualize the FUS and TDP-43, anti-FUS (1:400) and anti-TDP-43 (1:400) were used as the primary antibodies (Proteintech, Rosemont, IL; Cat #68262-1-Ig, Cat #60019-2-Ig). Antimouse and Antirabbit IgG conjugated with AF488 and Alexa Fluor 594 (AF594) (1:1000) (Cell Signaling) were employed as the secondary antibodies. The nuclei were stained with DAPI (Thermo Fischer Scientific, Cat # 62248). Images were taken with a spinning-disc confocal instrument (Yokogawa CSU-W1 50 μm 60 Pinhole, Nikon) on an inverted Ti-E microscope (Nikon, Japan) with a 100× oil-immersion objective (1.4 NA).

### Luciferase Reporter Assay

HEK293T cells were cultured in DMEM supplemented with 10% FBS and 1% penicillin–streptomycin and maintained at 37 °C in a humidified atmosphere with 5% CO_2_. A firefly luciferase reporter plasmid containing the GAL4 UAS sites upstream of the luciferase gene was used. Plasmids were prepared using a standard plasmid isolation kit. Cells were cultured in 24-well plates up to 70–80% confluency. Cotransfection in each well was performed using FuGENE (Promega, USA), with firefly luciferase plasmid and indicated amounts of pGAL4-DBD (DNA binding domain) or pGAL4-WDR5 or pGAL4-F266A-WDR5 under the control of a constitutive promoter. 24–48 h post-transfection, cells were washed with PBS and lysed. The lysates were transferred to a 96-well plate for luminescence measurements. The luciferase gene product was measured using a luciferase assay kit (Promega, USA) on a luminescence plate reader. The relative light units (RLU) were normalized as the fold activity of the control. All experiments were performed in triplicate, and the data were expressed as mean ± standard deviation (s.d.).

### Fluorescence Recovery after Photobleaching (FRAP)

These experiments were performed on the same confocal microscope as described above. 405 nm laser was used to bleach the region of interest on droplets and in living HeLa cells. Images were collected utilizing the 488 nm laser at a 400 ms exposure before and after bleaching. Images before bleaching were captured to determine the baseline. Upon data collection, images from each data set were analyzed within ImageJ, where they were treated as 16 bit stacks. FRAP curves were created using the ImageJ FRAP Calculator Macro plug-in, evaluating fluorescence intensity versus time. The data points were copied into Origin v9.7 (OriginLab, Northampton, MA). These curves were fitted using a single-exponential function. This approach enabled the determination of the recovery half-time.

### Image Processing and Statistical Analysis

Quantifying fluorescence intensity and size measurement of imaged particles were conducted using ImageJ. Particle analysis was performed using the particle analyzer tool in ImageJ. FRAP Calculator Macro plug-in in ImageJ v1.353f was employed for the FRAP curve analysis. Origin v9.7 (OriginLab, Northampton, MA) was used for statistical analyses and curve fittings. A steady-state analysis was performed to fit the BLI data. To determine the *K*_D_, a logistic regression function was used to fit the FP anisotropy data set.^104,107^ An unpaired two-tailed student *t*-test was used for comparison. Statistical significance was considered at a test level *p* < 0.05.

### Molecular Graphics

All protein graphics were prepared using the PyMOL Molecular Graphics System (Version 2.4.0 Schrödinger, LLC).

## Supplementary Material



## Notes

The AlphaFold 3 model and full-atomistic MD simulations of WDR5 self-interactions, the docking of IDR tail into the Win pocket, list of specific and nonspecific interactions of WDR5 dimers and tetramers, BLI measurements for probing the WDR5 self-association, steady-state fluorescence polarization (FP) anisotropy of the interaction of the R14N-NT peptide with the ΔN-WDR5 truncation variant, control BLI experiments for the WDR5 self-association, characterization of the WDR5 self-association using dynamic light scattering, the purification and characterization of the WDR5-mVenus protein, the structural prediction of the WDR5-mVenus fusion protein, phase separation assays of WDR5 in a cell-free environment, liquid–liquid phase separation of WDR5 in HEK-293T cells, fluorescence recovery after photobleaching of WDR5 condensates in cell-free environment, the purification and characterization of the MYC-mScarlet-I protein, the fusion, fission, and FRAP analysis of MYC droplets, the effect of a peptide inhibitor on the heterotypic MYC-WDR5 condensate, colocalization of Amyotrophic lateral sclerosis (ALS)-linked proteins with WDR5 condensates under hyperosmotic conditions, in vitro sequestration of RNA in WDR5 droplets, list of primers utilized in this study, Supplemental references (PDF)

## References

[1] Gomes E., Shorter J. (2019). The molecular language of membraneless organelles. J. Biol. Chem..

[2] Dignon G. L., Best R. B., Mittal J. (2020). Biomolecular Phase
Separation: From
Molecular Driving Forces to Macroscopic Properties. Annu. Rev. Phys. Chem..

[3] Musacchio A. (2022). On the role
of phase separation in the biogenesis of membraneless compartments. EMBO J..

[4] Hirose T., Ninomiya K., Nakagawa S., Yamazaki T. (2023). A guide to
membraneless
organelles and their various roles in gene regulation. Nat. Rev. Mol. Cell Biol..

[5] Cai D., Feliciano D., Dong P., Flores E., Gruebele M., Porat-Shliom N., Sukenik S., Liu Z., Lippincott-Schwartz J. (2019). Phase separation
of YAP reorganizes genome topology for long-term YAP target gene expression. Nat. Cell Biol..

[6] Yang P., Mathieu C., Kolaitis R. M., Zhang P., Messing J., Yurtsever U., Yang Z., Wu J., Li Y., Pan Q. (2020). G3BP1 Is a Tunable Switch that Triggers Phase
Separation
to Assemble Stress Granules. Cell.

[7] Liang J., Cai D. (2023). Membrane-less compartments
in the nucleus: Separated or connected
phases?. Curr. Opin. Cell Biol..

[8] Banani S. F., Lee H. O., Hyman A. A., Rosen M. K. (2017). Biomolecular
condensates:
organizers of cellular biochemistry. Nat. Rev.
Mol. Cell Biol..

[9] Mehta S., Zhang J. (2022). Liquid-liquid phase separation drives cellular function and dysfunction
in cancer. Nat. Rev. Cancer.

[10] Molliex A., Temirov J., Lee J., Coughlin M., Kanagaraj A. P., Kim H. J., Mittag T., Taylor J. P. (2015). Phase separation
by low complexity domains promotes stress granule assembly and drives
pathological fibrillization. Cell.

[11] Wang B., Zhang L., Dai T., Qin Z., Lu H., Zhang L., Zhou F. (2021). Liquid-liquid phase
separation in
human health and diseases. Signal Transduction
Targeted Ther..

[12] Strom A. R., Emelyanov A. V., Mir M., Fyodorov D. V., Darzacq X., Karpen G. H. (2017). Phase separation
drives heterochromatin domain formation. Nature.

[13] Larson A. G., Elnatan D., Keenen M. M., Trnka M. J., Johnston J. B., Burlingame A. L., Agard D. A., Redding S., Narlikar G. J. (2017). Liquid
droplet formation by HP1α suggests a role for phase separation
in heterochromatin. Nature.

[14] Boija A., Klein I. A., Sabari B. R., Dall’Agnese A., Coffey E. L., Zamudio A. V., Li C. H., Shrinivas K., Manteiga J. C., Hannett N. M. (2018). Transcription Factors
Activate Genes through the Phase-Separation Capacity of Their Activation
Domains. Cell.

[15] Sabari B. R., Dall’Agnese A., Boija A., Klein I. A., Coffey E. L., Shrinivas K., Abraham B. J., Hannett N. M., Zamudio A. V., Manteiga J. C. (2018). Coactivator condensation
at super-enhancers
links phase separation and gene control. Science.

[16] Hnisz D., Shrinivas K., Young R. A., Chakraborty A. K., Sharp P. A. (2017). A Phase Separation
Model for Transcriptional Control. Cell.

[17] Woodruff J. B., Hyman A. A., Boke E. (2018). Organization
and Function of Non-dynamic
Biomolecular Condensates. Trends Biochem. Sci..

[18] Kar M., Dar F., Welsh T. J., Vogel L. T., Kühnemuth R., Majumdar A., Krainer G., Franzmann T. M., Alberti S., Seidel C. A. M. (2022). Phase-separating RNA-binding
proteins form heterogeneous distributions of clusters in subsaturated
solutions. Proc. Natl. Acad. Sci. U. S. A..

[19] Lin Y., Protter D. S., Rosen M. K., Parker R. (2015). Formation and Maturation
of Phase-Separated Liquid Droplets by RNA-Binding Proteins. Mol. Cell.

[20] Skrott Z., Mistrik M., Andersen K. K., Friis S., Majera D., Gursky J., Ozdian T., Bartkova J., Turi Z., Moudry P. (2017). Alcohol-abuse drug disulfiram
targets cancer via p97
segregase adaptor NPL4. Nature.

[21] Protter D. S. W., Parker R. (2016). Principles and Properties
of Stress Granules. Trends Cell Biol..

[22] Mokin Y. I., Gavrilova A. A., Fefilova A. S., Kuznetsova I. M., Turoverov K. K., Uversky V. N., Fonin A. V. (2023). Nucleolar- and Nuclear-Stress-Induced
Membrane-Less Organelles: A Proteome Analysis through the Prism of
Liquid-Liquid Phase Separation. Int. J. Mol.
Sci..

[23] Alberti S., Hyman A. A. (2021). Biomolecular condensates
at the nexus of cellular stress,
protein aggregation disease and ageing. Nat.
Rev. Mol. Cell Biol..

[24] Bryan A. F., Wang J., Howard G. C., Guarnaccia A. D., Woodley C. M., Aho E. R., Rellinger E. J., Matlock B. K., Flaherty D. K., Lorey S. L. (2020). WDR5
is a conserved regulator of protein synthesis gene expression. Nucleic Acids Res..

[25] Guarnaccia A. D., Rose K. L., Wang J., Zhao B., Popay T. M., Wang C. E., Guerrazzi K., Hill S., Woodley C. M., Hansen T. J. (2021). Impact of WIN site inhibitor on the WDR5 interactome. Cell Rep..

[26] Guarnaccia A. D., Tansey W. P. (2018). Moonlighting with
WDR5: A Cellular Multitasker. J. Clin. Med..

[27] Thomas L. R., Wang Q., Grieb B. C., Phan J., Foshage A. M., Sun Q., Olejniczak E. T., Clark T., Dey S., Lorey S. (2015). Interaction with WDR5 promotes target gene recognition
and tumorigenesis by MYC. Mol. Cell. Biochem..

[28] Ahmad M., Imran A., Movileanu L. (2024). Overlapping
characteristics of weak
interactions of two transcriptional regulators with WDR5. Int. J. Biol. Macromol..

[29] Kaustov L., Lemak A., Wu H., Faini M., Fan L., Fang X., Zeng H., Duan S., Allali-Hassani A., Li F. (2019). The MLL1 trimeric catalytic complex is a dynamic conformational
ensemble stabilized by multiple weak interactions. Nucleic Acids Res..

[30] Xue H., Yao T., Cao M., Zhu G., Li Y., Yuan G., Chen Y., Lei M., Huang J. (2019). Structural basis of
nucleosome recognition and modification by MLL methyltransferases. Nature.

[31] Ge Z., Song E. J., Kawasawa Y. I., Li J., Dovat S., Song C. (2016). WDR5 high expression and its effect on tumorigenesis in leukemia. Oncotarget.

[32] Wu Y., Diao P., Li Z., Zhang W., Wang D., Wang Y., Cheng J. (2018). Overexpression of WD repeat domain
5 associates with aggressive clinicopathological features and unfavorable
prognosis in head neck squamous cell carcinoma. J. Oral Pathol. Med..

[33] Cui Z., Li H., Liang F., Mu C., Mu Y., Zhang X., Liu J. (2018). Effect of high WDR5 expression on the hepatocellular carcinoma prognosis. Oncol. Lett..

[34] Thomas L. R., Foshage A. M., Weissmiller A. M., Tansey W. P. (2015). The MYC-WDR5 Nexus
and Cancer. Cancer Res..

[35] Chen X., Xie W., Gu P., Cai Q., Wang B., Xie Y., Dong W., He W., Zhong G., Lin T., Huang J. (2015). Upregulated WDR5 promotes
proliferation, self-renewal and chemoresistance
in bladder cancer via mediating H3K4 trimethylation. Sci. Rep..

[36] Sun W., Guo F., Liu M. (2018). Up-regulated
WDR5 promotes gastric cancer formation
by induced cyclin D1 expression. J. Cell. Biochem..

[37] Wang K. C., Yang Y. W., Liu B., Sanyal A., Corces-Zimmerman R., Chen Y., Lajoie B. R., Protacio A., Flynn R. A., Gupta R. A. (2011). A long
noncoding RNA maintains active chromatin
to coordinate homeotic gene expression. Nature.

[38] Yang Y. W., Flynn R. A., Chen Y., Qu K., Wan B., Wang K. C., Lei M., Chang H. Y. (2014). Essential
role of
lncRNA binding for WDR5 maintenance of active chromatin and embryonic
stem cell pluripotency. Elife.

[39] Thomas L. R., Adams C. M., Wang J., Weissmiller A. M., Creighton J., Lorey S. L., Liu Q., Fesik S. W., Eischen C. M., Tansey W. P. (2019). Interaction of the oncoprotein transcription
factor MYC with its chromatin cofactor WDR5 is essential for tumor
maintenance. Proc. Natl. Acad. Sci. U.S.A..

[40] Thomas L. R., Adams C. M., Fesik S. W., Eischen C. M., Tansey W. P. (2020). Targeting
MYC through WDR5. Mol. Cell. Oncol..

[41] Oksuz O., Henninger J. E., Warneford-Thomson R., Zheng M. M., Erb H., Vancura A., Overholt K. J., Hawken S. W., Banani S. F., Lauman R. (2023). Transcription factors interact with RNA to regulate
genes. Mol. Cell.

[42] Zhou Y., Su J. M., Samuel C. E., Ma D. (2019). Measles Virus
Forms
Inclusion Bodies with Properties of Liquid Organelles. J. Virol..

[43] Li W., Wu L., Jia H., Lin Z., Zhong R., Li Y., Jiang C., Liu S., Zhou X., Zhang E. (2021). The low-complexity
domains of the KMT2D protein regulate histone monomethylation transcription
to facilitate pancreatic cancer progression. Cell. Mol. Biol. Lett..

[44] Namitz K. E. W., Tan S., Cosgrove M. S. (2023). Hierarchical
assembly of the MLL1
core complex regulates H3K4 methylation and is dependent on temperature
and component concentration. J. Biol. Chem..

[45] Mayse L. A., Imran A., Wang Y., Ahmad M., Oot R. A., Wilkens S., Movileanu L. (2023). Evaluation
of Nanopore Sensor Design
Using Electrical and Optical Analyses. ACS Nano.

[46] Li Y., Han J., Zhang Y., Cao F., Liu Z., Li S., Wu J., Hu C., Wang Y., Shuai J. (2016). Structural
basis for activity regulation of MLL family methyltransferases. Nature.

[47] Wang P., Dreger M., Madrazo E., Williams C. J., Samaniego R., Hodson N. W., Monroy F., Baena E., Sánchez-Mateos P., Hurlstone A., Redondo-Muñoz J. (2018). WDR5 modulates cell
motility and morphology and controls nuclear changes induced by a
3D environment. Proc. Natl. Acad. Sci. U. S.
A..

[48] Siladi A. J., Wang J., Florian A. C., Thomas L. R., Creighton J. H., Matlock B. K., Flaherty D. K., Lorey S. L., Howard G. C., Fesik S. W. (2022). WIN
site inhibition disrupts a subset of WDR5
function. Sci. Rep..

[49] Dao T. P., Kolaitis R. M., Kim H. J., O’Donovan K., Martyniak B., Colicino E., Hehnly H., Taylor J. P., Castañeda C. A. (2018). Ubiquitin Modulates Liquid-Liquid Phase Separation
of UBQLN2 via Disruption of Multivalent Interactions. Mol. Cell.

[50] Zhu J., Jiang L. (2022). Liquid-Liquid Phase Separation Bridges Physics, Chemistry,
and Biology. Langmuir.

[51] Hyman A. A., Weber C. A., Jülicher F. (2014). Liquid-liquid
phase separation in
biology. Annu. Rev. Cell Dev. Biol..

[52] Watson J. L., Seinkmane E., Styles C. T., Mihut A., Krüger L. K., McNally K. E., Planelles-Herrero V.
J., Dudek M., McCall P. M., Barbiero S. (2023). Macromolecular condensation
buffers intracellular water potential. Nature.

[53] Riback J. A., Katanski C. D., Kear-Scott J. L., Pilipenko E. V., Rojek A. E., Sosnick T. R., Drummond D. A. (2017). Stress-Triggered
Phase Separation Is an Adaptive, Evolutionarily Tuned Response. Cell.

[54] Abramson J., Adler J., Dunger J., Evans R., Green T., Pritzel A., Ronneberger O., Willmore L., Ballard A. J., Bambrick J. (2024). Accurate structure prediction of biomolecular
interactions with AlphaFold 3. Nature.

[55] Patel A., Dharmarajan V., Cosgrove M. S. (2008). Structure of WDR5 bound to mixed
lineage leukemia protein-1 peptide. J. Biol.
Chem..

[56] Patel A., Vought V. E., Dharmarajan V., Cosgrove M. S. (2008). A conserved arginine-containing
motif crucial for the assembly and enzymatic activity of the mixed
lineage leukemia protein-1 core complex. J.
Biol. Chem..

[57] Dharmarajan V., Lee J. H., Patel A., Skalnik D. G., Cosgrove M. S. (2012). Structural
basis for WDR5 interaction (Win) motif recognition in human SET1 family
histone methyltransferases. J. Biol. Chem..

[58] Zhang P., Lee H., Brunzelle J. S., Couture J. F. (2012). The plasticity of WDR5 peptide-binding
cleft enables the binding of the SET1 family of histone methyltransferases. Nucleic Acids Res..

[59] Honorato R. V., Trellet M. E., Jiménez-García B., Schaarschmidt J. J., Giulini M., Reys V., Koukos P. I., Rodrigues J., Karaca E., van Zundert G. C. P. (2024). The HADDOCK2.4 web server for integrative modeling
of biomolecular complexes. Nat. Protoc..

[60] Imran A., Moyer B. S., Kalina D., Duncan T. M., Moody K. J., Wolfe A. J., Cosgrove M. S., Movileanu L. (2022). Convergent
Alterations of a Protein Hub Produce Divergent Effects Within a Binding
Site. ACS Chem. Biol..

[61] Jacobs W. M., Oxtoby D. W., Frenkel D. (2014). Phase separation
in solutions with
specific and nonspecific interactions. J. Chem.
Phys..

[62] Krainer G., Welsh T. J., Joseph J. A., Espinosa J. R., Wittmann S., de Csilléry E., Sridhar A., Toprakcioglu Z., Gudiškytė G., Czekalska M. A. (2021). Reentrant liquid condensate phase of proteins
is stabilized by hydrophobic
and non-ionic interactions. Nat. Commun..

[63] Rossi A. M., Taylor C. W. (2011). Analysis of protein-ligand
interactions by fluorescence
polarization. Nat. Protoc..

[64] Odho Z., Southall S. M., Wilson J. R. (2010). Characterization
of a novel WDR5-binding
site that recruits RbBP5 through a conserved motif to enhance methylation
of histone H3 lysine 4 by mixed lineage leukemia protein-1. J. Biol. Chem..

[65] Alicea-Velázquez N. L., Shinsky S. A., Loh D. M., Lee J. H., Skalnik D. G., Cosgrove M. S. (2016). Targeted Disruption of the Interaction between WD-40
Repeat Protein 5 (WDR5) and Mixed Lineage Leukemia (MLL)/SET1 Family
Proteins Specifically Inhibits MLL1 and SETd1A Methyltransferase Complexes. J. Biol. Chem..

[66] Imran A., Moyer B. S., Canning A. J., Kalina D., Duncan T. M., Moody K. J., Wolfe A. J., Cosgrove M. S., Movileanu L. (2021). Kinetics of
the multitasking high-affinity Win binding site of WDR5 in restricted
and unrestricted conditions. Biochem. J..

[67] Imran A., Moyer B. S., Wolfe A. J., Cosgrove M. S., Makarov D. E., Movileanu L. (2022). Interplay
of Affinity and Surface Tethering in Protein
Recognition. J. Phys. Chem. Lett..

[68] Jachimska B., Wasilewska M., Adamczyk Z. (2008). Characterization of globular protein
solutions by dynamic light scattering, electrophoretic mobility, and
viscosity measurements. Langmuir.

[69] Nagai T., Ibata K., Park E. S., Kubota M., Mikoshiba K., Miyawaki A. (2002). A variant of yellow
fluorescent protein with fast and
efficient maturation for cell-biological applications. Nat. Biotechnol..

[70] Jumper J., Evans R., Pritzel A., Green T., Figurnov M., Ronneberger O., Tunyasuvunakool K., Bates R., Žídek A., Potapenko A. (2021). Highly accurate protein structure prediction
with AlphaFold. Nature.

[71] Tunyasuvunakool K., Adler J., Wu Z., Green T., Zielinski M., Žídek A., Bridgland A., Cowie A., Meyer C., Laydon A. (2021). Highly accurate protein structure prediction for the
human proteome. Nature.

[72] Mitrea D. M., Cika J. A., Stanley C. B., Nourse A., Onuchic P. L., Banerjee P. R., Phillips A. H., Park C. G., Deniz A. A., Kriwacki R. W. (2018). Self-interaction
of NPM1 modulates multiple mechanisms
of liquid-liquid phase separation. Nat. Commun..

[73] Guillén-Boixet J., Kopach A., Holehouse A. S., Wittmann S., Jahnel M., Schlüßler R., Kim K., Trussina I., Wang J., Mateju D. (2020). RNA-Induced
Conformational
Switching and Clustering of G3BP Drive Stress Granule Assembly by
Condensation. Cell.

[74] Mekonnen G., Djaja N., Yuan X., Myong S. (2023). Advanced imaging techniques
for studying protein phase separation in living cells and at single-molecule
level. Curr. Opin. Chem. Biol..

[75] Alberti S., Gladfelter A., Mittag T. (2019). Considerations and
Challenges in
Studying Liquid-Liquid Phase Separation and Biomolecular Condensates. Cell.

[76] Shen Y., Chen A., Wang W., Shen Y., Ruggeri F. S., Aime S., Wang Z., Qamar S., Espinosa J. R., Garaizar A. (2023). The liquid-to-solid
transition of FUS is promoted
by the condensate surface. Proc. Natl. Acad.
Sci. U.S.A..

[77] Jalihal A. P., Schmidt A., Gao G., Little S. R., Pitchiaya S., Walter N. G. (2021). Hyperosmotic phase separation: Condensates beyond inclusions,
granules and organelles. J. Biol. Chem..

[78] Bindels D. S., Haarbosch L., van Weeren L., Postma M., Wiese K. E., Mastop M., Aumonier S., Gotthard G., Royant A., Hink M. A., Gadella T. W. (2017). mScarlet: a bright
monomeric red fluorescent protein for cellular imaging. Nat. Methods.

[79] Mayse L. A., Wang Y., Ahmad M., Movileanu L. (2024). Real-Time
Measurement of a Weak Interaction of a Transcription Factor Motif
with a Protein Hub at Single-Molecule Precision. ACS Nano.

[80] Gao C., Gu J., Zhang H., Jiang K., Tang L., Liu R., Zhang L., Zhang P., Liu C., Dai B., Song J. (2022). Hyperosmotic-stress-induced liquid-liquid phase separation of ALS-related
proteins in the nucleus. Cell Rep..

[81] Portz B., Lee B. L., Shorter J. (2021). FUS and TDP-43
Phases in Health and
Disease. Trends Biochem. Sci..

[82] Rhine K., Vidaurre V., Myong S. (2020). RNA Droplets. Annu. Rev. Biophys..

[83] Dou Y., Milne T. A., Tackett A. J., Smith E. R., Fukuda A., Wysocka J., Allis C. D., Chait B. T., Hess J. L., Roeder R. G. (2005). Physical association
and coordinate function of the
H3 K4 methyltransferase MLL1 and the H4 K16 acetyltransferase MOF. Cell.

[84] Wysocka J., Swigut T., Milne T. A., Dou Y., Zhang X., Burlingame A. L., Roeder R. G., Brivanlou A. H., Allis C. D. (2005). WDR5 associates with histone H3 methylated at K4 and
is essential for H3 K4 methylation and vertebrate development. Cell.

[85] Dhungel, P. ; Cantu, F. ; Hernandez, C. ; Yang, Z. In Vitro Transcribed RNA-based Luciferase Reporter Assay to Study Translation Regulation in Poxvirus-infected Cells. J. Visualized Exp. 2019, (147). 10.3791/59626 PMC7236376 31107441

[86] DeNicola G. M., Chen P. H., Mullarky E., Sudderth J. A., Hu Z., Wu D., Tang H., Xie Y., Asara J. M., Huffman K. E. (2015). NRF2 regulates serine
biosynthesis in non-small cell lung cancer. Nat. Genet..

[87] Cotton J.
L., Li Q., Ma L., Park J. S., Wang J., Ou J., Zhu L. J., Ip Y. T., Johnson R. L., Mao J. (2017). YAP/TAZ and
Hedgehog Coordinate Growth and Patterning in Gastrointestinal Mesenchyme. Dev. Cell.

[88] Franzmann T. M., Alberti S. (2019). Protein Phase Separation as a Stress Survival Strategy. Cold Spring Harbor Perspect. Biol..

[89] Jung K. H., Sun J., Hsiung C. H., Lian X. L., Liu Y., Zhang X. (2023). Nuclear bodies
protect phase separated proteins from degradation in stressed proteome. bioRxiv.

[90] Phillips J. C., Hardy D. J., Maia J. D. C., Stone J. E., Ribeiro J. V., Bernardi R. C., Buch R., Fiorin G., Hénin J., Jiang W. (2020). Scalable
molecular dynamics on CPU and GPU architectures
with NAMD. J. Chem. Phys..

[91] Hart K., Foloppe N., Baker C. M., Denning E. J., Nilsson L., Mackerell A. D. (2012). Optimization of the CHARMM additive
force field for DNA: Improved treatment of the BI/BII conformational
equilibrium. J. Chem. Theory Comput..

[92] Jorgensen W. L., Chandrasekhar J., Madura J. D., Impey R. W., Klein M. L. (1983). Comparison
of simple potential functions for simulating liquid water. J. Phys. Chem..

[93] Beglov D., Roux B. (1994). Finite Representation
of An Infinite Bulk System - Solvent Boundary
Potential for Computer Simulations. J. Chem.
Phys..

[94] Andersen H.
C. (1983). RATTLE
- A velocity version of the shake algorithm for molecular-dynamics
calculations. J. Comput. Phys..

[95] Miyamoto S., Kollman P. A. (1992). SETTLE - An analytical
version of the shake and rattle
algorithm for rigid water models. J. Comput.
Chem..

[96] Darden T., York D., Pedersen L. (1993). Particle mesh
Ewald - An N.LOG­(N)
method for the Ewald sums in large systems. J. Chem. Phys..

[97] Martyna G. J., Tobias D. J., Klein M. L. (1994). Constant-pressure molecular dynamics
algorithms. J. Chem. Phys..

[98] Geng, C. L. ; Narasimhan, S. ; Rodrigues, J. ; Bonvin, A. Information-Driven, Ensemble Flexible Peptide Docking Using HADDOCK. In Modeling Peptide-Protein Interactions: Methods and Protocols; SchuelerFurman, O. , London, N. , Eds.; Methods in Molecular Biology, 2017; Springer; Vol. 1561, pp 109–138. 10.1007/978-1-4939-6798-8_8 28236236

[99] Michaud-Agrawal N., Denning E. J., Woolf T. B., Beckstein O. (2011). Software News
and Updates MDAnalysis: A Toolkit for the Analysis of Molecular Dynamics
Simulations. J. Comput. Chem..

[100] Gowers, R. J. ; Linke, M. ; Barnoud, J. ; Reddy, T. J. E. ; Melo, M. N. ; Seyler, S. L. ; Dotson, D. L. ; Domanski, J. ; Buchoux, S. ; Kenney, I. M. ; Beckstein, O. MD Analysis: A Python package for the rapid analysis of molecular dynamics simulations. In The 15th Python in Science Conference; Benthall, S. , Rostrup, S. , Eds.: Austin, TX, 2016; pp 98–105. 10.25080/majora-629e541a-00e.

[101] Hunter J. D. (2007). Matplotlib: A 2D graphics environment. Comput. Sci. Eng..

[102] Mayse L. A., Imran A., Larimi M. G., Cosgrove M. S., Wolfe A. J., Movileanu L. (2022). Disentangling the recognition complexity
of a protein hub using a nanopore. Nat. Commun..

[103] Ahmad M., Ha J. H., Mayse L. A., Presti M. F., Wolfe A. J., Moody K. J., Loh S. N., Movileanu L. (2023). A generalizable
nanopore sensor for highly specific protein detection at single-molecule
precision. Nat. Commun..

[104] Wolfe A. J., Si W., Zhang Z., Blanden A. R., Hsueh Y. C., Gugel J. F., Pham B., Chen M., Loh S. N., Rozovsky S. (2017). Quantification of membrane
protein-detergent complex interactions. J. Phys.
Chem. B.

[105] Bobbili K. B., Sivaji N., Priya B., Suguna K., Surolia A. (2023). Structure
and interactions of the phloem lectin (phloem
protein 2) Cus17 from Cucumis sativus. Structure.

[106] Moser B., Hochreiter B., Herbst R., Schmid J. A. (2017). Fluorescence
colocalization microscopy analysis can be improved by combining object-recognition
with pixel-intensity-correlation. Biotechnol.
J..

[107] Wolfe A. J., Hsueh Y. C., Blanden A. R., Mohammad M. M., Pham B., Thakur A. K., Loh S. N., Chen M., Movileanu L. (2017). Interrogating Detergent Desolvation
of Nanopore-Forming
Proteins by Fluorescence Polarization Spectroscopy. Anal. Chem..

