# Supplementary material for: A Site-Specific Self-Association of a Protein Hub Drives Its Phase Separation
Source: ACS Chem Biol. 2025 Dec 24;21(1):46–61. doi: 10.1021/acschembio.5c00424 (PMC12813979; doi:10.1021/acschembio.5c00424)
Supplement: Supplementary file 1 [file cb5c00424_si_001.pdf]

SUPPORTING INFORMATION FILE

A Site-Specific Self-Association of a Protein Hub Drives Its Phase Separation

Mohammad Ahmad[1], Yazheng Wang[1,2], Siddharth Krishnan[3], Ali Imran[1], Aleksei Aksimentiev[3], and Liviu Movileanu[1,2,4,5*]

[1]*Department of Physics, Syracuse University, 201 Physics Building, Syracuse, New York 13244, United States*

[2]*Department of Biomedical and Chemical Engineering, Syracuse University, 329 Link Hall, Syracuse, New York 13244, United States*

[3]*Department of Physics, University of Illinois at Urbana-Champaign, Urbana, Illinois 61801, United States*

[4]*The BioInspired Institute, Syracuse University, Syracuse, New York, 13244, United States*

[5]*Department of Biology, Syracuse University, 114 Life Sciences Complex, Syracuse, New York 13244, United States*

Keywords: WDR5; MYC; Biolayer interferometry; Molecular dynamics simulations; Intrinsically disordered protein; Liquid-liquid phase separation.

*The corresponding author:
Liviu Movileanu, PhD, Phone: 315-443-8078; E-mail: lmovilea@syr.edu

1. The AlphaFold 3 model and full-atomistic MD simulations of WDR5 self-interactions.

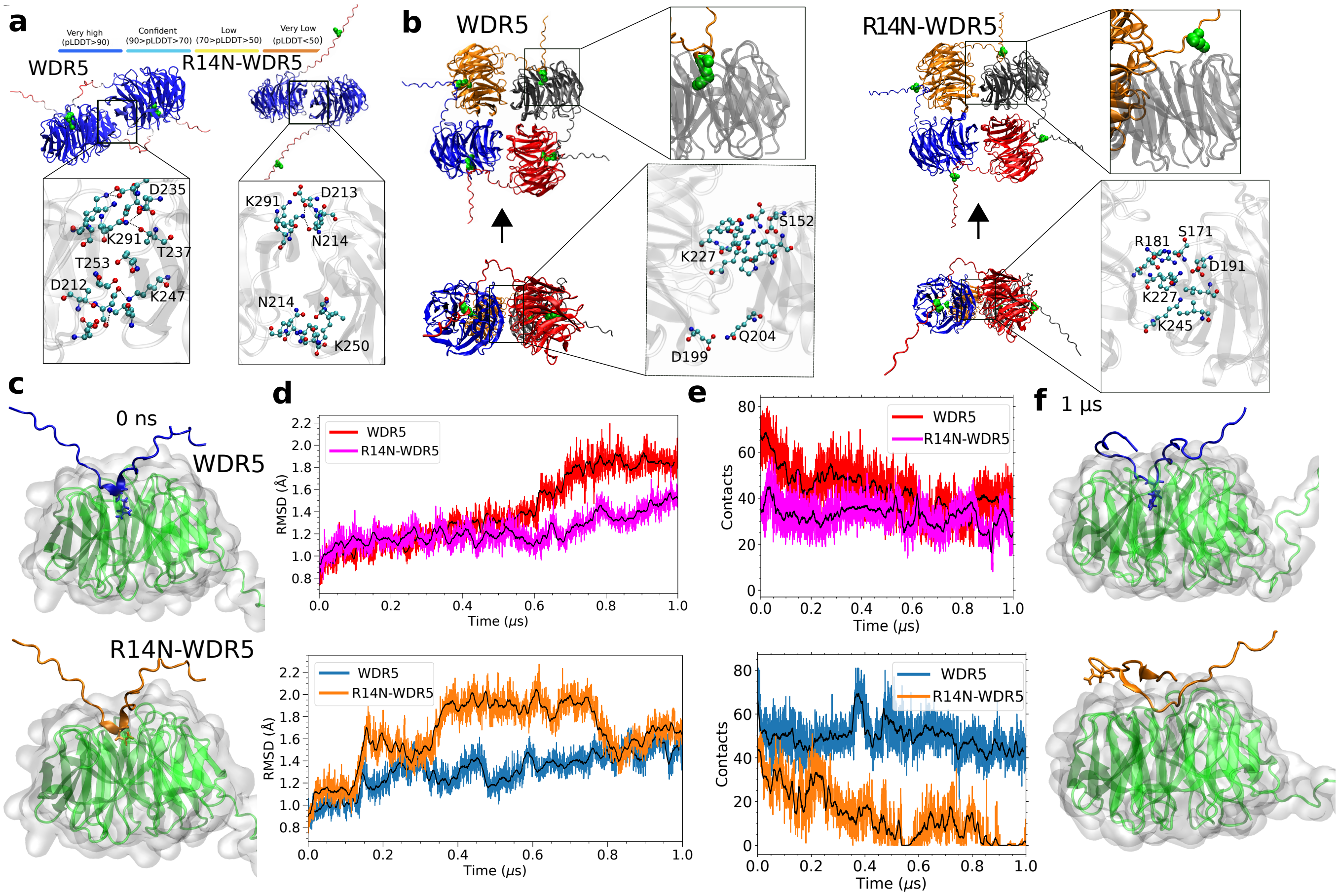

<u>Supplementary Figure S1.</u> The role of Arg-14 in WDR5 self-interactions. (a) The AlphaFold 3 model[1] of the WDR5 (left) and R14N-WDR5 mutant (right) of the dimer colored according to the average predicted local distance difference test (pLDDT) score of each residue. The atoms of Arg-14 or Asn-14 residues are represented using green van der Waals (vdW) spheres. The region between the two folded domains is zoomed in to show interfacial contacts using a ball-and-stick model with cyan, red, and blue spheres representing carbon, oxygen, and nitrogen atoms, respectively**.** A few residues at the interfaces are annotated. **(b)** The Alpha Fold 3 model[1] of the wild-type WDR5 (left) and R14N-WDR5 mutant (right) of the tetramer. The arrow indicates the side from which the view is taken. A zoomed-in view at the top shows one of the Win pockets of WDR5. A zoomed-in view at the bottom illustrates contact interactions between residues of the folded domains. **(c)** Initial structural models of the self-interaction of the WDR5 (top) and R14N-WDR5 (bottom). One WDR5 protein is shown as both a semi-transparent molecular surface and as a secondary structure cartoon (green). Two N-terminal peptides, each attached to the second WDR5 protein of the dimer (whose folded domain is not shown), are illustrated as blue (WDR5) or orange (R14N-WDR5). **(d)** C_α atoms' root mean square deviation (RMSD) of each folded domain in the dimer for the WDR5 and R14N-WDR5 structures relative to the crystal structure 4Y7R[2] throughout a 1 μs-duration MD simulation. The top panel shows the data for one monomer, and the bottom panel illustrates the data for the second monomer. The black lines show a 10-ns running average of the instantaneous data. **(e)** The top plot presents the number of contacts formed by the AR or AN motifs of the WDR5 tail of the first monomer with the Win site of the second monomer during the all-atom MD simulation of the dimer. The bottom plot shows the same data for the second Win site. The black lines show a 10-ns running average of the instantaneous data. **(f)** The final conformations of WDR5 and R14N-WDR5 after 1μs all-atom MD simulation.

2. The docking of IDR tail into the Win pocket.

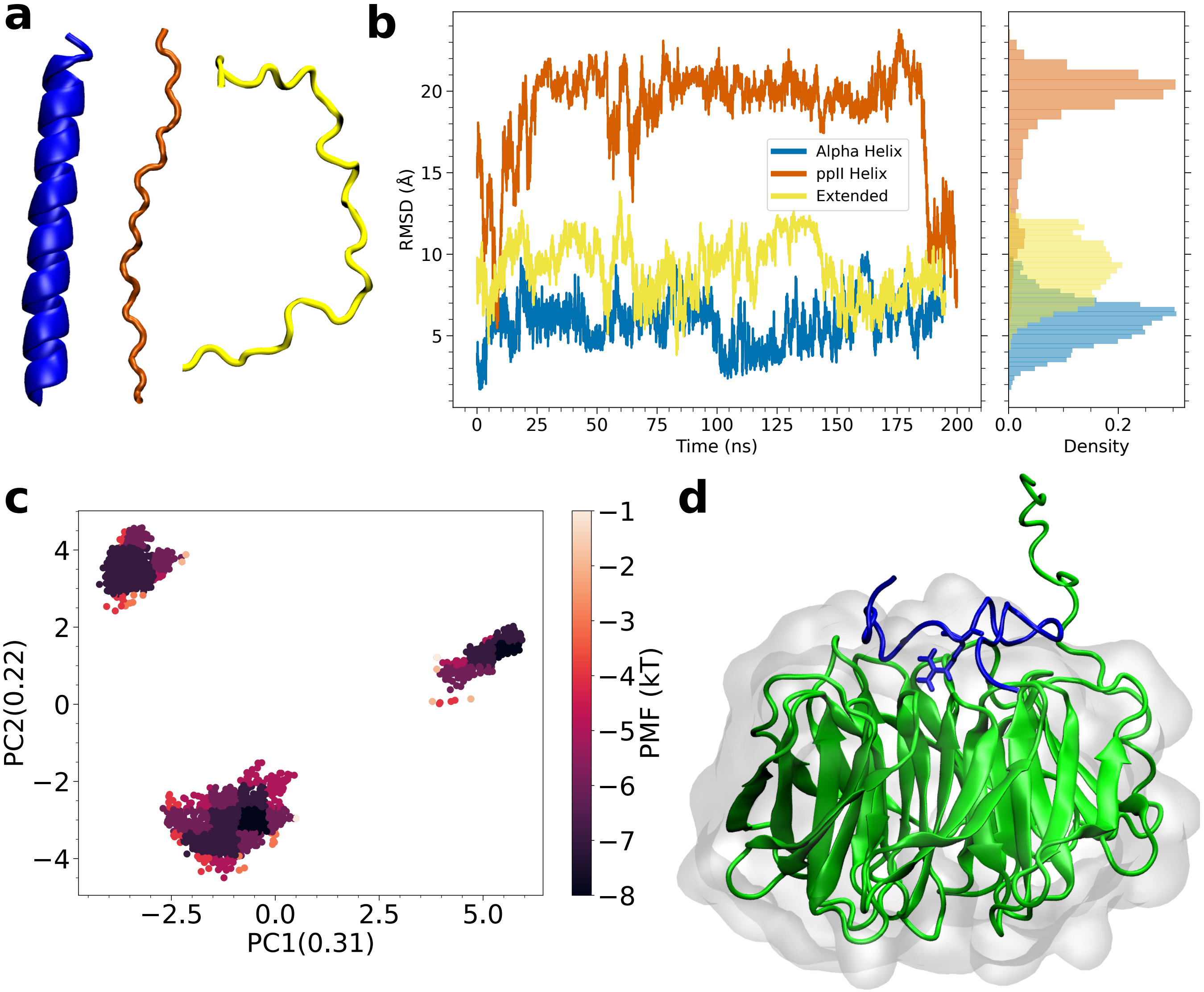

Supplementary Figure S2. The docking of the IDR tail into the Win pocket. (a) The starting configuration for all-atom MD simulations, where the tail sequence adopts the structure of an alpha-helix (blue), poly-proline(ppII) helix (vermillion), or an extended chain (yellow). **(b)** RMSD of the tail's C_α coordinates from its initial configuration during the equilibrium simulation. The histograms for the RMSD values are shown to the right of the traces. **(c)** Phase space explored by the MD simulations projected onto the two principal axes. The variance is shown along the axes in the brackets. The components were found using dihedral angle principal component analysis (dPCA).[3] The color gradient represents the potential of mean force (PMF) calculated as $k_B T \log(P)$, where P is the number of configurations in the bin. The lowest energy configuration from each of the 30 bins and the initial three configurations were selected for subsequent docking calculations. **(d)** Top-scoring structure from docking studies.[4] The N-terminal disordered peptide tail is shown in blue. Arg-14 is displayed using a representation of molecular bonds. One WDR5 protein is shown as both a semi-transparent molecular surface and as a secondary structure cartoon (green).

3. List of specific and nonspecific interactions of WDR5 dimers and tetramers.

<u>Supplementary Table S1.</u> The mapping of the specific contacts at the WDR5-WDR5 interface between the IDR of the first WDR5 protein and the Win pocket of the second WDR5 protein. These results were obtained using the top-scored AlphaFold 3[1] structure of the WDR5 dimer in **Supplementary Figure S1a**. The cut-off distance for identifying these contacts was 3.5 Å. Hydrogen atoms were not considered. The first residue in each bond belongs to the IDR of the first WDR5 protein, among which Ala-13 and Arg-14 belong to the Win motif,[5] whereas the second residue belongs to the Win pocket of the second WDR5 protein. Among these, amino acids S91, F133, C261, and F263 belong to the Win site[5-8] of WDR5.

Contacts	Distance (Å)
A12-A65	3.4
A12-I90	3.3
A12-D107	3.4
A13-S91	3.3
A13-D107	3.4
A13-D107	3.0
A13-D107	3.1
R14-F133	3.2
R14-F133	3.3
R14-S91	3.1
R14-F263	3.5
R14-S49	3.0
R14-S91	3.3
R14-S91	2.7
R14-C261	3.0
R14-F263	3.5
R14-F263	3.3
R14-F133	2.8

Supplementary Table S2. The mapping of the nonspecific contacts at the interface between the folded domains of the WDR5 dimer. These results were obtained using the top-scored AlphaFold 3[1] structure of the WDR5 dimer in **Supplementary Figure S1a**. The cut-off distance for identifying these contacts was 3.5 Å. Hydrogen atoms were not considered. The first residue in each bond belongs to one of the WDR5 proteins in the dimer, whereas the second one belongs to the other WDR5.

**Contacts**	**Distance (Å)**
K291-T237	2.6
K291-D235	3.0
K291-T237	3.3
K247-D212	3.4
K247-D212	2.7
T253-T253	2.8
D235-T290	3.3
T237-K291	3.0
T237-K291	3.4
D212-K247	2.8
D213-K250	3.0
D235-K291	2.8

Supplementary Table S3. The mapping of the nonspecific contacts at the interface between the folded domains of the R14N-WDR5 dimer. These results were obtained using the top-scored AlphaFold 3[1] structure for the R14N-WDR5 dimer in **Supplementary Figure S1a**. The cut-off distance for identifying these contacts was 3.5 Å. Hydrogen atoms were not considered. The first residue in each bond belongs to one of the dimer's R14N-WDR5 proteins, whereas the second one belongs to the other R14N-WDR5.

Contacts	Distance (Å)
P215-K291	2.8
N214-K291	3.3
D213-K250	2.5
D235-T253	3.3
P215-K291	3.3
P215-K291	2.4
N214-K291	3.5
N214-L249	3.4
N214-L249	3.2
N214-L249	2.3
T253-D235	3.3
K239-T251	3.2
T251-K239	3.2
K291-N214	3.4
K291-N214	3.3
K291-P215	2.9
K291-P215	2.6
K291-D213	3.2

Supplementary Table S4. The mapping of the nonspecific contacts at the interface between the folded domains of the WDR5 tetramer. These results were obtained using the top-scored AlphaFold 3[1] structure of the WDR5 tetramer in **Supplementary Figure S1b**. The cut-off distance for identifying these contacts was 3.5 Å. Hydrogen atoms were not considered. The first residue in each bond belongs to one of the WDR5 monomers in the tetramer, whereas the second one belongs to the neighboring WDR5 monomer. The monomers are named A, B, C, and D. The contacts between each monomer are listed below.

A-B interface		B-C interface		C-D interface		D-A interface	
**Contacts**	**Distance (Å)**	**Contacts**	**Distance (Å)**	**Contacts**	**Distance (Å)**	**Contacts**	**Distance (Å)**
I210-K245	3.1	I210-K245	3.0	I210-K245	3.4	I210-K245	3.4
I210-K245	3.1	I210-K245	3.5	I210-K245	2.9	I210-K245	3.4
I210-K245	3.3	I210-K245	2.9	I210-K245	3.1	I210-K245	2.8
I210-K245	2.9	I210-K245	2.9	L194-K245	2.8	I210-K245	3.0
L194-K245	2.8	I210-K245	3.1	I210-K245	3.2	I210-K245	3.1
D192-K227	3.0	I210-K245	3.5	I210-K245	3.5	I210-K245	3.6
D192-K227	3.5	I210-K245	3.2	D192-K227	3.1	I210-K245	3.0
L194-K227	3.2	L194-K245	3.3	L194-K227	3.2	L194-K227	3.5
L194-K227	3.1	D192-K227	3.3	L194-K227	3.1	L194-K227	2.9
S171-N225	3.1	D192-K227	2.9	T208-K245	3.5	L194-K227	2.9
S171-N225	3.1	L194-K227	3.0	S171-K227	3.2	L194-K245	2.9
S171-K227	3.2	L194-K227	2.9	S171-Y228	3.0	S171-N225	2.6
S171-Y228	2.9	T208-S244	3.2	R196-S244	3.4	S171-Y228	3.2
D192-K227	2.5	S171-N225	2.9	R196-K227	3.2	T208-K245	3.4
T208-S244	3.5	S171-N225	2.5	T208-S244	3.1	T208-S244	3.0
T208-S244	3.1	R196-K227	3.1	D192-K227	2.6	R196-K227	3.0
R196-K227	3.1	P168-N225	3.3	S171-N225	3.1	D192-K227	3.0
H170-N225	3.1	P168-N225	3.1	S171-N225	3.0	D192-K227	2.6
		P168-D182	3.3	E151-N225	3.5	S171-N225	3.4
		Q204-D199	3.4	H170-N225	3.3	S171-N225	2.7
		Q204-D199	3.0	P168-N225	3.3	P168-N225	3.2
		T166-D182	3.5	P168-N225	3.4	H170-N225	3.4
				P168-N225	2.6	H170-N225	3.1
				Q204-D199	3.4	P168-N225	2.8
				Q204-D199	3.0	Q204-D199	3.3

<u>Supplementary Table S5.</u> The mapping of the nonspecific contacts at the interface between the folded domains of the R14N-WDR5 tetramer. These results were obtained using the top-scored AlphaFold 3[1] structure for the R14N-WDR5 tetramer in **Supplementary Figure S1b**. The cut-off distance for identifying these contacts was 3.5 Å. Hydrogen atoms were not considered. The first residue in each bond belongs to one of the R14N-WDR5 monomers in the R14N-WDR5 tetramer, whereas the second one belongs to the neighboring R14N-WDR5 monomer. The monomers are named A, B, C, and D. The contacts between each monomer are listed below.

**A-B interface**		**B-C interface**		**C-D interface**		**D-A interface**	
**Contacts**	**Distance (Å)**	**Contacts**	**Distance (Å)**	**Contacts**	**Distance (Å)**	**Contacts**	**Distance (Å)**
Q204-D199	3.2	E151-N225	2.8	D192-K227	3.3	L194-K245	3.1
Q204-D199	3.1	E151-N225	3.5	D192-K228	3.2	L194-K246	3.0
T208-K245	3.3	S171-Y228	3.5	D192-K229	2.9	L194-K247	3.1
T208-S244	3.3	S171-Y228	3.5	D192-K230	3.4	L194-K248	3.5
I210-K245	2.9	S171-Y228	2.7	D192-K231	3.0	L194-K249	2.9
I210-K245	3.4	S171-K227	3.4	D192-K232	3.5	L194-K250	3.2
I210-K245	2.8	S171-K227	3.4	D192-K233	3.5	L194-K251	3.0
I210-K245	3.1	S171-K227	3.1	D192-K234	3.4	L194-K252	3.3
I210-K245	2.8	S171-N225	3.0	D192-K235	2.9	L194-K253	2.8
I210-K245	3.0	S171-N225	3.2	D192-K236	3.3	L194-K254	3.1
R196-K227	3.1	P168-R181	3.1	D192-K237	3.3	L194-K255	3.1
L194-K245	2.7	P168-N225	3.5	D192-K238	3.2	L194-K256	3.1
L194-K227	3.0	H170-N225	3.2	D192-K239	2.8	L194-K257	3.2
L194-K227	3.1	D192-K227	3.2	D192-K240	2.5	L194-K258	2.7
I210-K245	3.5	P168-D182	3.5	D192-K241	3.5	L194-K259	3.5
P168-R181	3.4	P168-N225	2.8	D192-K242	3.3	L194-K260	3.4
P168-N225	2.9	D192-K227	3.4	D192-K243	2.5	L194-K261	3.4
P168-R181	3.4	D192-K227	2.5	D192-K244	2.8	L194-K262	3.3
P168-R181	2.8	R196-S244	3.3	D192-K245	3.5	L194-K263	2.7
S171-N225	3.0	R196-K227	3.4	D192-K246	3.5	L194-K264	3.4
D192-K227	2.6	S152-R181	3.0	D192-K247	2.9	L194-K265	3.3
S171-K227	3.3	T166-D182	3.2	D192-K248	3.4	L194-K266	3.1
S171-Y228	3.3	L167-R181	3.4	D192-K249	3.4	L194-K267	3.2
D192-K227	3.1	D192-K227	3.0	D192-K250	2.6	L194-K268	3.1

S152-R181	3.5	L194-K227	3.3	D192-K251	2.8	L194-K269	3.3
E151-N225	3.5	L194-K227	3.3	D192-K252	2.9	L194-K270	3.2
H170-N225	3.4	L194-K245	2.6	D192-K253	3.4	L194-K271	3.0
S171-N225	3.1	L194-K245	3.2	D192-K254	3.4	L194-K272	3.1
D192-K227	3.5	T208-S244	3.4	D192-K255	3.4	L194-K273	3.2
		I210-K245	3.1	D192-K256	2.8	L194-K274	3.5
		I210-K245	3.2	D192-K257	3.0	L194-K275	3.5
		I210-K245	3.5	D192-K258	2.9	L194-K276	2.7
		I210-K245	3.4	D192-K259	2.9	L194-K277	3.4
		I210-K245	3.0	D192-K260	3.3		
		I210-K245	3.5	D192-K261	3.1		
		Q204-D199	2.9	D192-K262	3.4		
		Q204-D199	3.1	D192-K263	3.4		
		Q204-D199	3.4	D192-K264	3.3		
				D192-K265	3.3		
				D192-K266	3.1		
				D192-K267	2.9		
				D192-K268	3.0		
				D192-K269	3.3		
				D192-K270	3.4		
				D192-K271	3.3		

4. BLI measurements for probing the WDR5 self-association.

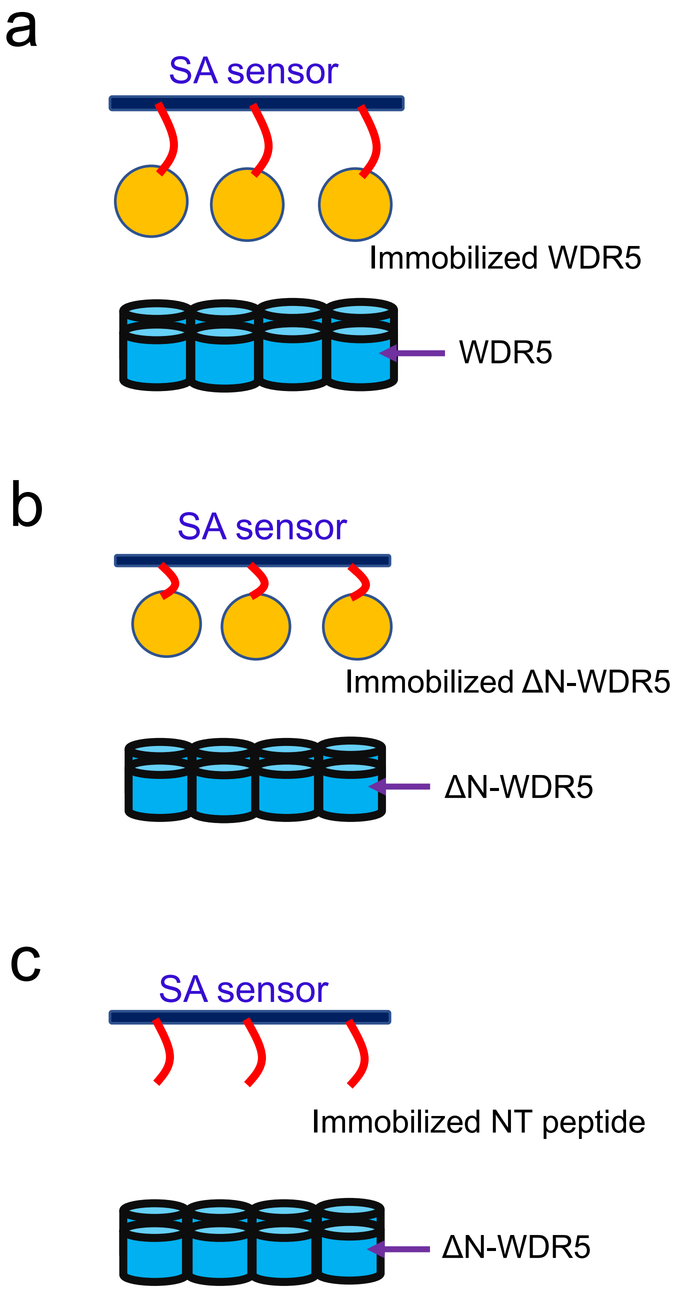

Supplementary Figure S3. Schematic representation of BLI measurements for probing the WDR5 self-association. (a) A system for testing the WDR5-WDR5 interaction. **(b)** A system for testing the interaction of ΔN-WDR5 with ΔN-WDR5. **(c)** A system for testing the interaction of the NT peptide with the ΔN-WDR5 truncated variant; Biotin-tagged immobilization reagents were loaded onto streptavidin (SA)-coated BLI sensors. These were 20 nM WDR5, 20 nM ΔN-WDR5, or 5 nM NT peptide. Titration series of WDR5 or ΔN-WDR5 were injected as analytes, and the corresponding association and dissociation phases were recorded, as previously reported.[9-11]

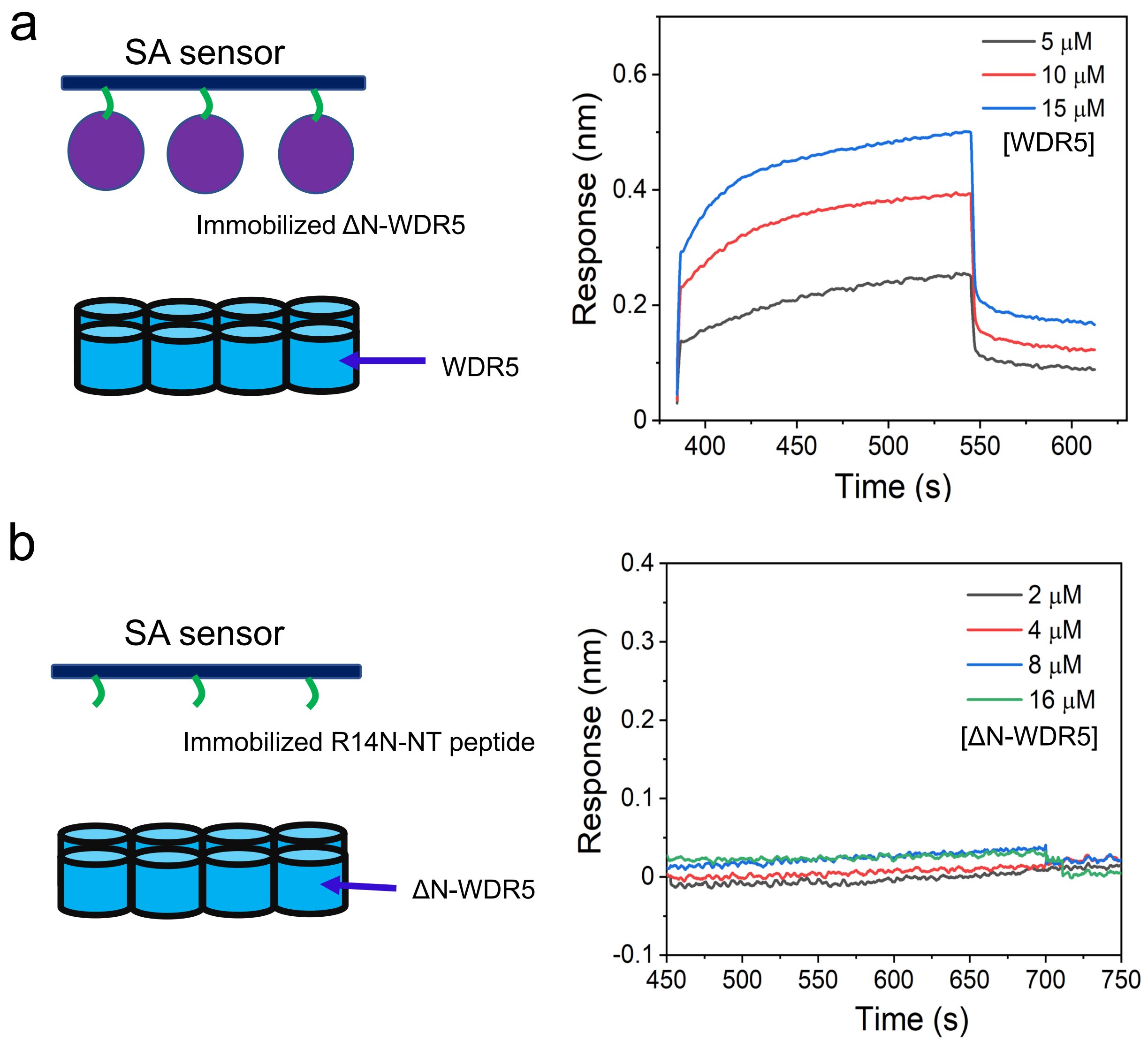

<u>Supplementary Figure S4.</u> **Experimental evidence for the self-association of WDR5 using BLI. (a)** Schematic representation of the BLI system for characterizing the interaction of the immobilized ΔN-WDR5 with WDR5 (*left*). BLI sensorgrams were obtained for this interaction (*right*). Biotinylated ΔN-WDR5 was immobilized onto streptavidin (SA)-coated BLI sensors and WDR5 was kept free in the wells. **(b)** Schematic representation of the BLI system for characterizing the interaction of the R14N-NT peptide with the ΔN-WDR5 truncation variant (*left*). BLI sensorgrams were obtained for this interaction (*right*). Biotinylated R14N-NT peptide was immobilized onto streptavidin (SA)-coated BLI sensors, and ΔN-WDR5 was kept free in the wells. In both panels, the concentrations of the free analyte were indicated in the legend. The sensorgrams included the association and dissociation phases, corresponding to sensor wells with and without the free analyte, respectively.

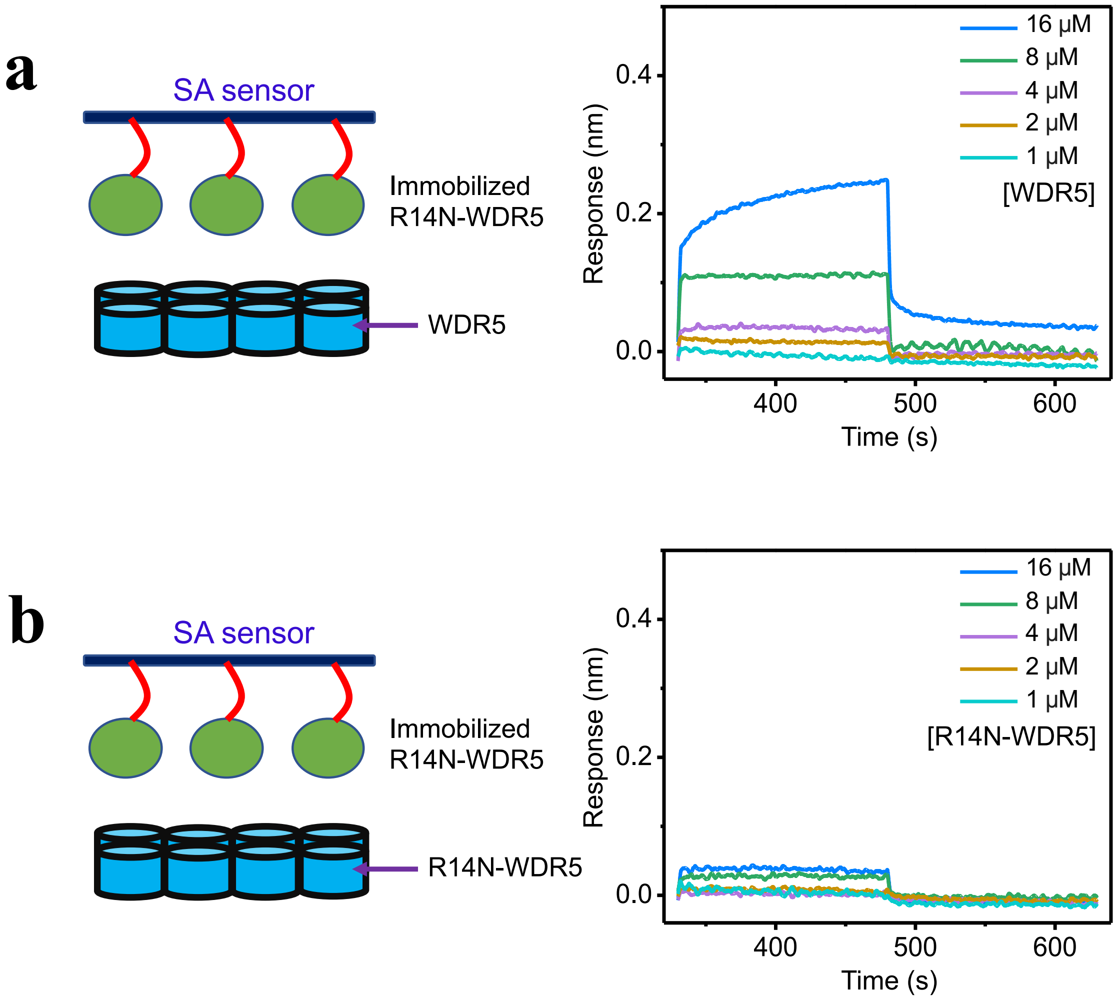

Supplementary Figure S5. Direct experimental evidence for the critical importance of Arg-14 in mediating the WDR5 self-association interaction. (a) Schematic representation of the BLI system for characterizing the interaction of the immobilized R14N-WDR5 with WDR5 (*left*). BLI sensorgrams were obtained for this interaction (*right*). Biotinylated R14N-WDR5 was immobilized onto streptavidin (SA)-coated BLI sensors, and WDR5 was kept free in the wells. **(b)** A schematic representation of the BLI system for characterizing the interaction of the immobilized R14N-WDR5 with R14N-WDR5 (*left*). BLI sensorgrams were obtained for this interaction (*right*). Biotinylated R14N-WDR5 was immobilized onto streptavidin (SA)-coated BLI sensors, and R14N-WDR5 was kept free in the wells. In both panels, the concentrations of the free analyte were indicated in the legend. The sensorgrams included the association and dissociation phases, corresponding to sensor wells with and without the free analyte, respectively.

5. Steady-state fluorescence polarization (FP) anisotropy of the interaction of the R14N-NT peptide with the ΔN-WDR5 truncation variant.

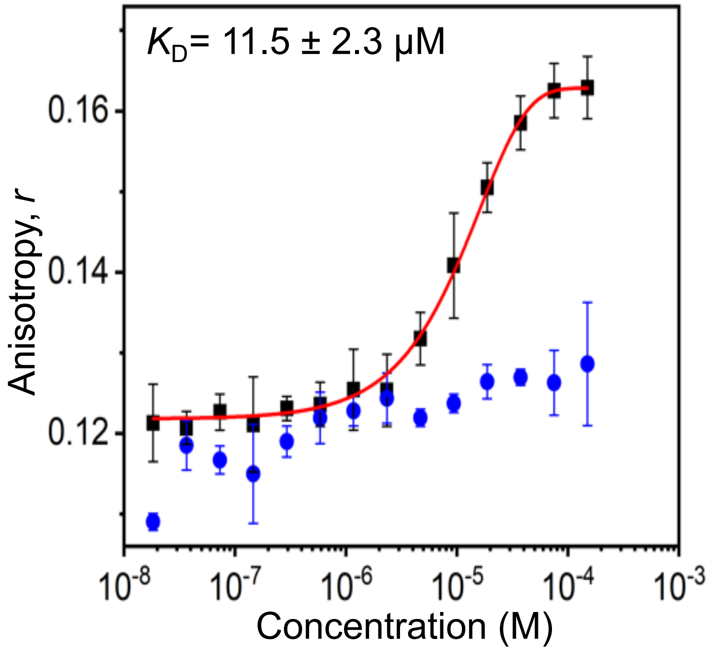

<u>Supplementary Figure S6.</u> **Steady-state fluorescence polarization (FP) anisotropy curves of the interaction of the R14N-NT peptide (blue ovals) with the ΔN-WDR5 truncation variant and its comparison with the native NT peptide (black rectangles).** Both peptides were labeled with rhodamine at the N terminus. The final concentration of the labeled peptides in each well was 20 nM. They were titrated against various concentrations of the ΔN-WDR5 truncation variant. Data indicate mean ± s.d. from n = 3 independent experiments.

6. Control BLI experiments for the WDR5 self-association.

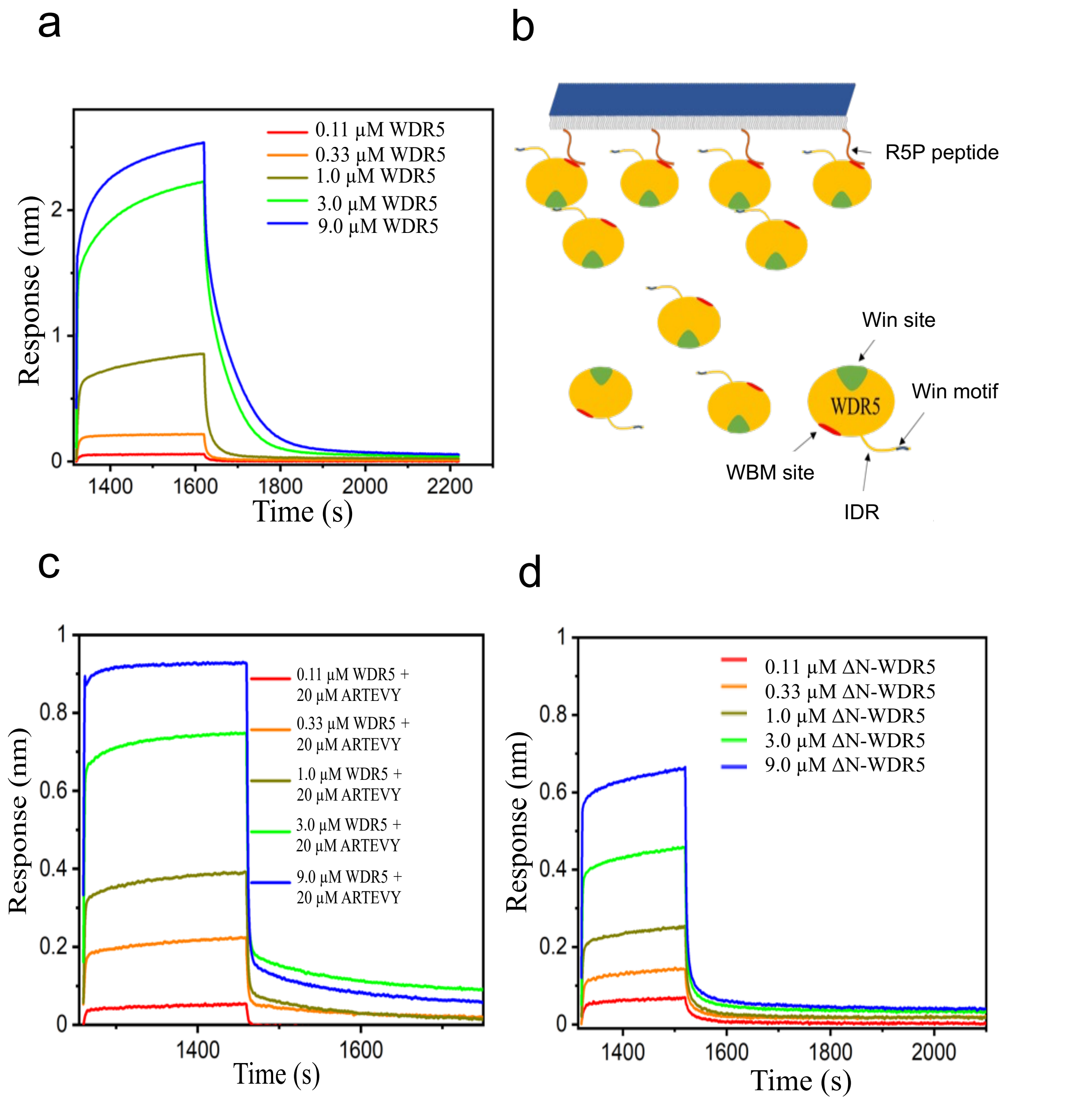

<u>Supplementary Figure S7.</u> BLI sensorgrams for the R5P-WDR5 interaction. **(a)** BLI sensorgrams obtained for the R5P-WDR5 interaction. **(b)** Depiction of self-association of WDR5 while R5P was attached to the streptavidin (SA)-coated BLI sensor surface. **(c)** BLI sensorgrams were obtained for the R5P-WDR5 interaction with ARTEVY spiked in the wells. **(d)** The R5P-ΔN-WDR5 interaction. 5 nM biotinylated R5P was immobilized onto streptavidin (SA)-coated BLI sensors. The sensors were dipped into wells containing different concentrations of analytes. The sensorgrams were obtained using five different WDR5 or ΔN-WDR5 concentrations, as shown in the legends.

Supplementary Table S6. The rate constants of association (k_{on}) and dissociation (k_{off}) of the interaction of R5P with WDR5 or ΔN-WDR5. For WDR5, 20 μM ARTEVY was spiked in the well to block the Win site and prevent the WDR5 self-association. Values were obtained using BLI sensorgrams in **Supplementary Figure S7cd.** These sensorgrams were fitted to obtain k_{on} and k_{off}, which were used to obtain K_D indirectly. Triplicates were performed. Data indicate mean ± s.d.

Protein	k_{on} ($\times 10^4$ $M^{-1}s^{-1}$)	k_{off} ($\times 10^{-1}$ s^{-1})	K_D (μM)
**WDR5**	17 ± 2	2.4 ± 0.3	1.4 ± 0.4
**ΔN-WDR5**	9.4 ± 1.1	1.4 ± 0.1	1.5 ± 0.3

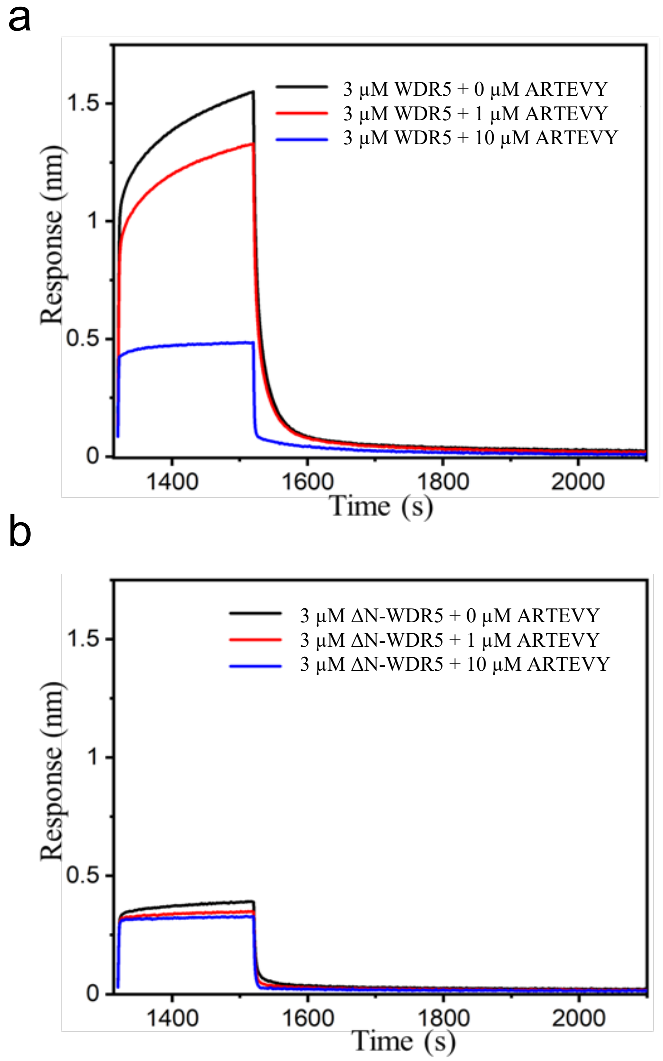

Supplementary Figure S8. Blocking the Win site using ARTEVY. The ARTEVY peptide inhibits the R5P-WDR5 interaction. Biotinylated R5P was immobilized onto streptavidin (SA)-coated BLI sensors. **(a)** WDR5. **(b)** ΔN-WDR5.

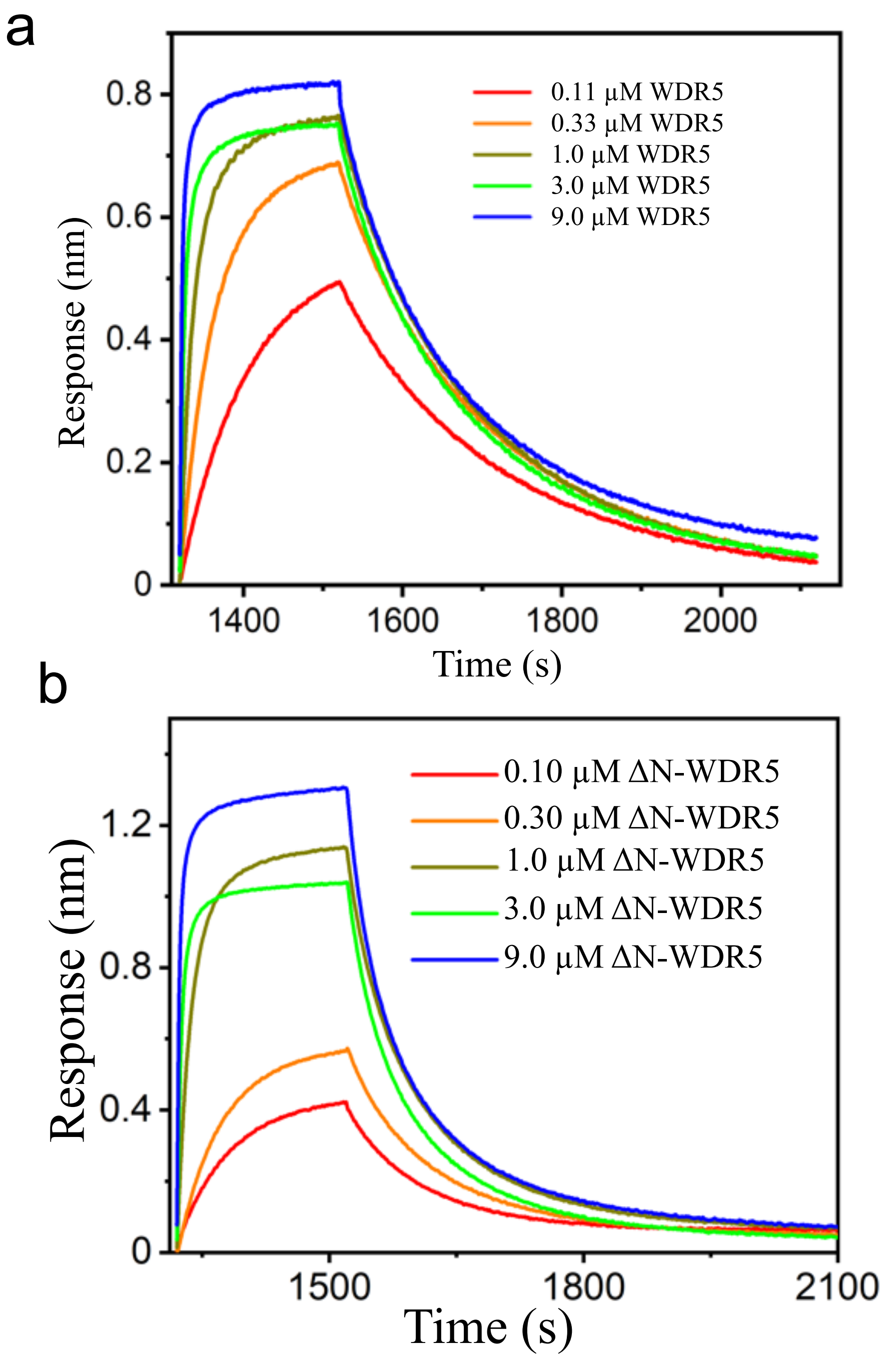

<u>Supplementary Figure S9.</u> **BLI sensorgrams for the interaction of the MLL3$_{\mathbf{Win}}$ peptide with WDR5 or ΔN-WDR5. (a)** WDR5. **(b)** ΔN-WDR5. Biotinylated MLL3$_{Win}$ peptide was immobilized onto streptavidin-coated BLI sensors. The sensors were dipped into wells containing different concentrations of either WDR5 or ΔN-WDR5.

<u>Supplementary Table S7.</u> The rate constants of association (k_{on}) and dissociation (k_{off}) of the interaction of the MLL3$_{Win}$ peptide with WDR5 or ΔN-WDR5. Values were obtained using BLI sensorgrams in **Supplementary Figure S9.** These sensorgrams were fitted to obtain k_{on} and k_{off}, which were used to obtain K_D indirectly. Triplicates were performed. Data indicate mean ± s.d.

Protein	k_{on} ($\times10^4$ $M^{-1}s^{-1}$)	k_{off} ($\times10^{-2}$ s^{-1})	K_D (nM)
**WDR5**	6.6 ± 1.8	1.2 ± 0.2	190 ± 60
**ΔN-WDR5**	6.3 ± 2.8	0.97 ± 0.04	170 ± 70

7. Characterization of the WDR5 self-association using dynamic light scattering.

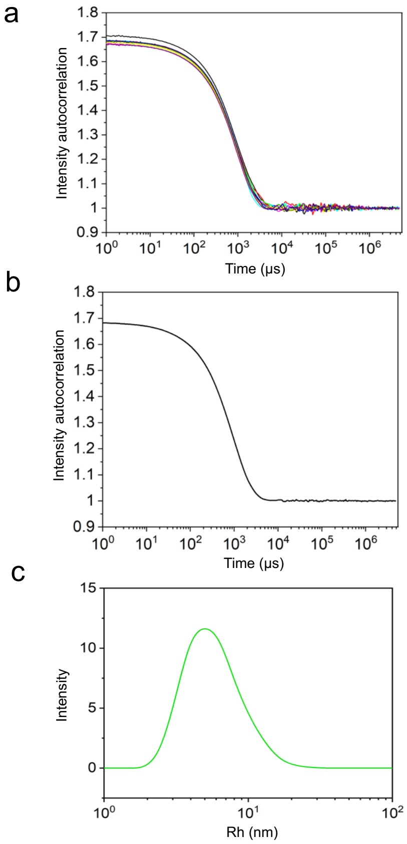

<u>Supplementary Figure S10.</u> Dynamic light scattering (DLS) of bovine serum albumin (BSA). (a) DLS data for BSA was acquired at a concentration of 50 μM, illustrating the correlation functions for 10 data acquisitions. **(b)** The average correlation function. Data for the averaging process was used from (a). **(c)** The corresponding scattered intensity distribution versus the hydrodynamic radius, R_h. The hydrodynamic radius was 4.2 ± 0.6 nm, which is in accordance with previously reported data.[12]

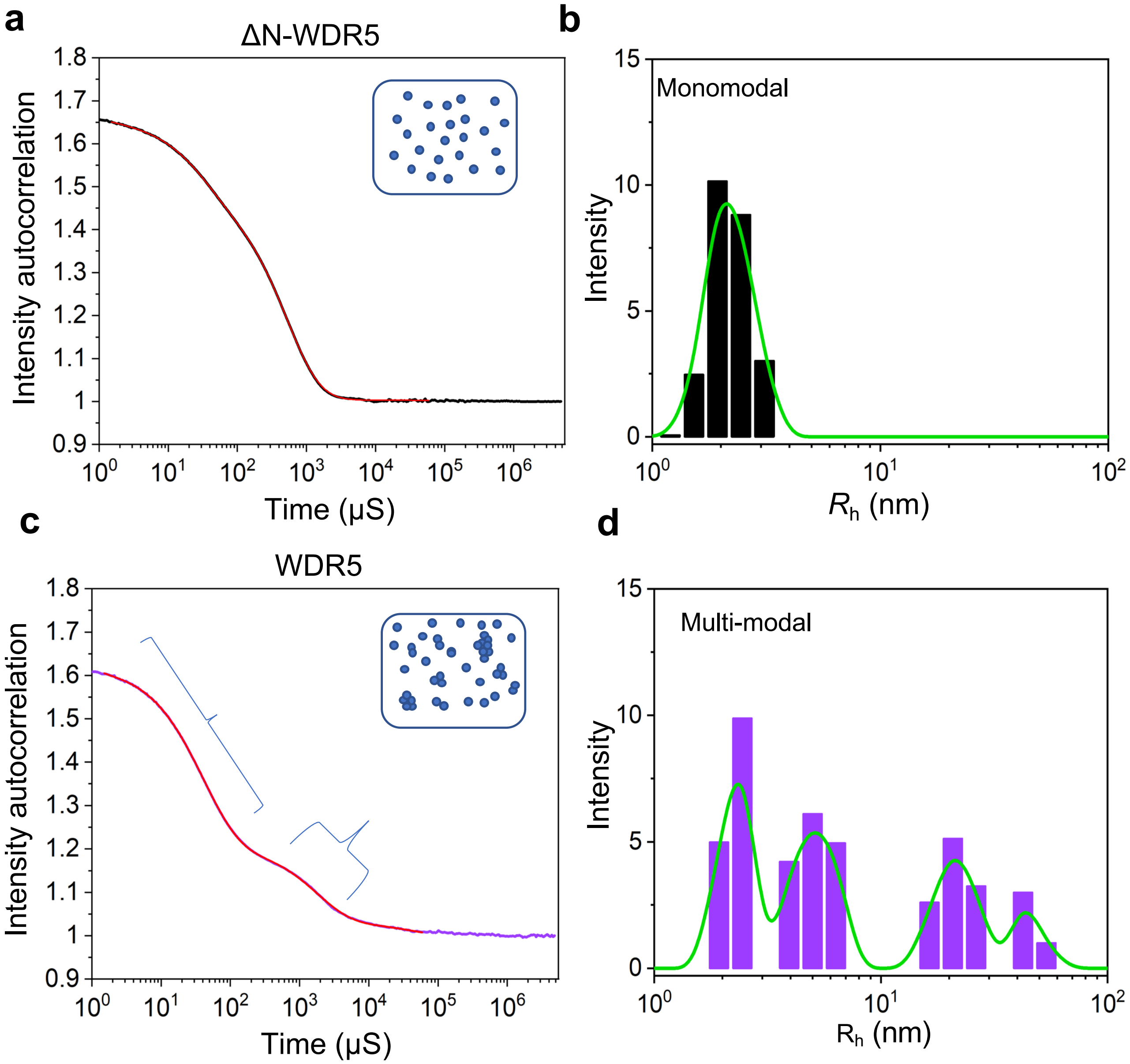

<u>Supplementary Figure S11.</u> **Characterization of the self-association of WDR5 using dynamic light scattering (DLS). (a)** DLS data for ΔN-WDR5 was acquired at 25 µM ΔN-WDR5, illustrating the raw correlation function. τ, the relaxation time constant for ΔN-WDR5 (mean ± s.d.), was 0.23 ± 0.02 ms. **(b)** The corresponding scattered intensity distribution versus the hydrodynamic radius, R_h, using (a). **(c)** DLS data for WDR5 was acquired at 25 µM WDR5, illustrating the raw correlation function. In the case of WDR5, the relaxation time constant for the rapidly decaying phase (mean ± s.d.), τ_1, was 0.024 ± 0.002 ms, and for the slowly decaying phase, τ_2, was 1.28 ± 0.30 ms. **(d)** The corresponding scattered intensity distribution versus R_h using (c).

8. The purification and characterization of the WDR5-mVenus protein.

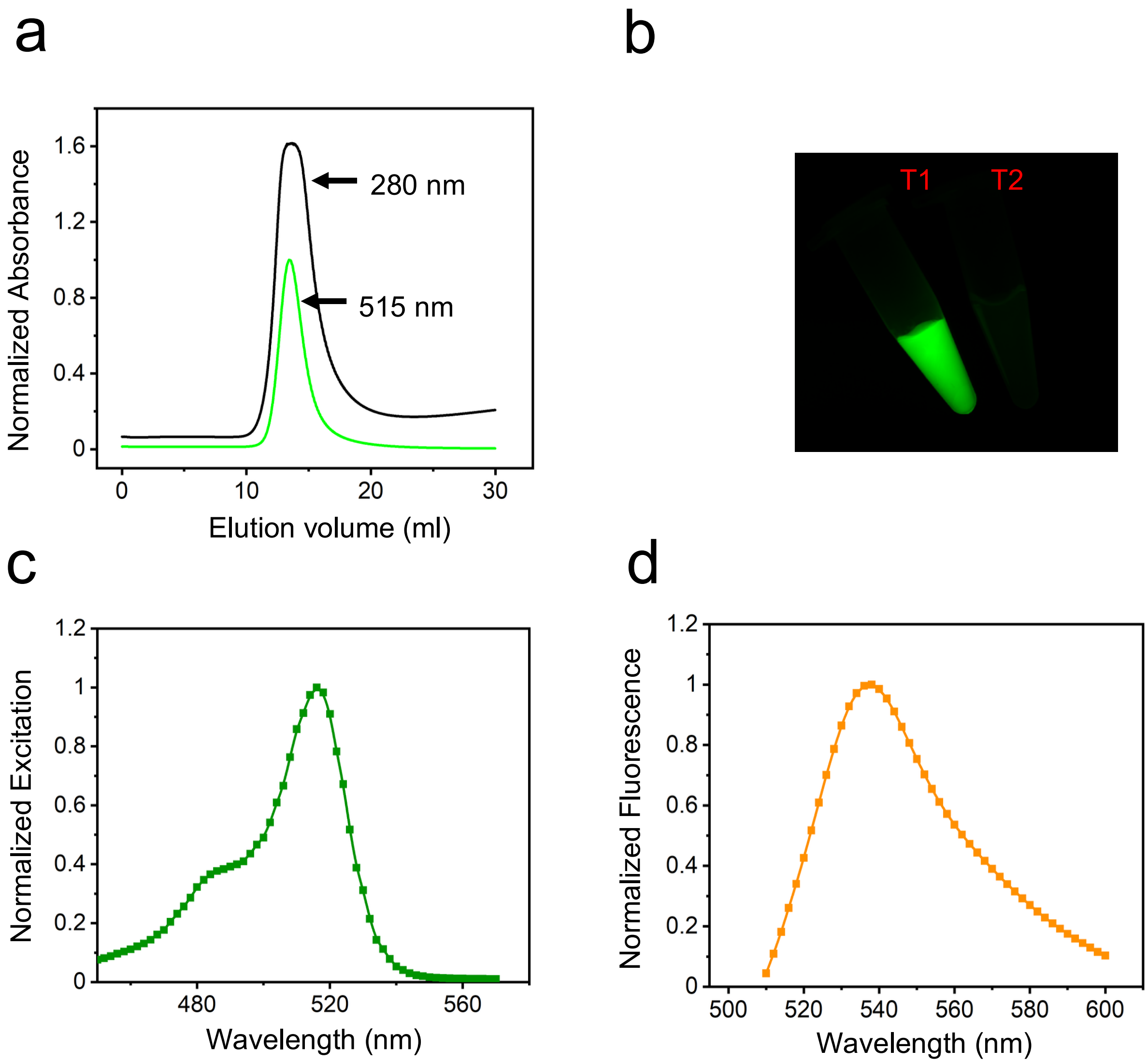

Supplementary Figure S12. The purification and characterization of the WDR5-mVenus protein. (a) A **c**hromatogram of the purified WDR5-mVenus fusion protein showing the normalized absorbance values at 280 and 515 nm. **(b)** An image of WDR5-mVenus containing microfuge tube (T1) and buffer (T2) was captured using the MP imaging system (Bio-Rad, Hercules, CA) equipped with the Alexa fluorophore 488 (AF488) filter. **(c)** The normalized excitation spectrum of the purified WDR5-mVenus protein recorded from 450 to 570 nm using a SpectraMax i3 microplate reader (Molecular Devices, San Jose, CA). **(d)** The normalized emission spectrum of the purified WDR5-mVenus protein recorded from 510 to 600 nm using a SpectraMax i3 microplate reader (Molecular Devices).

9. The structural prediction of the WDR5-mVenus fusion protein.

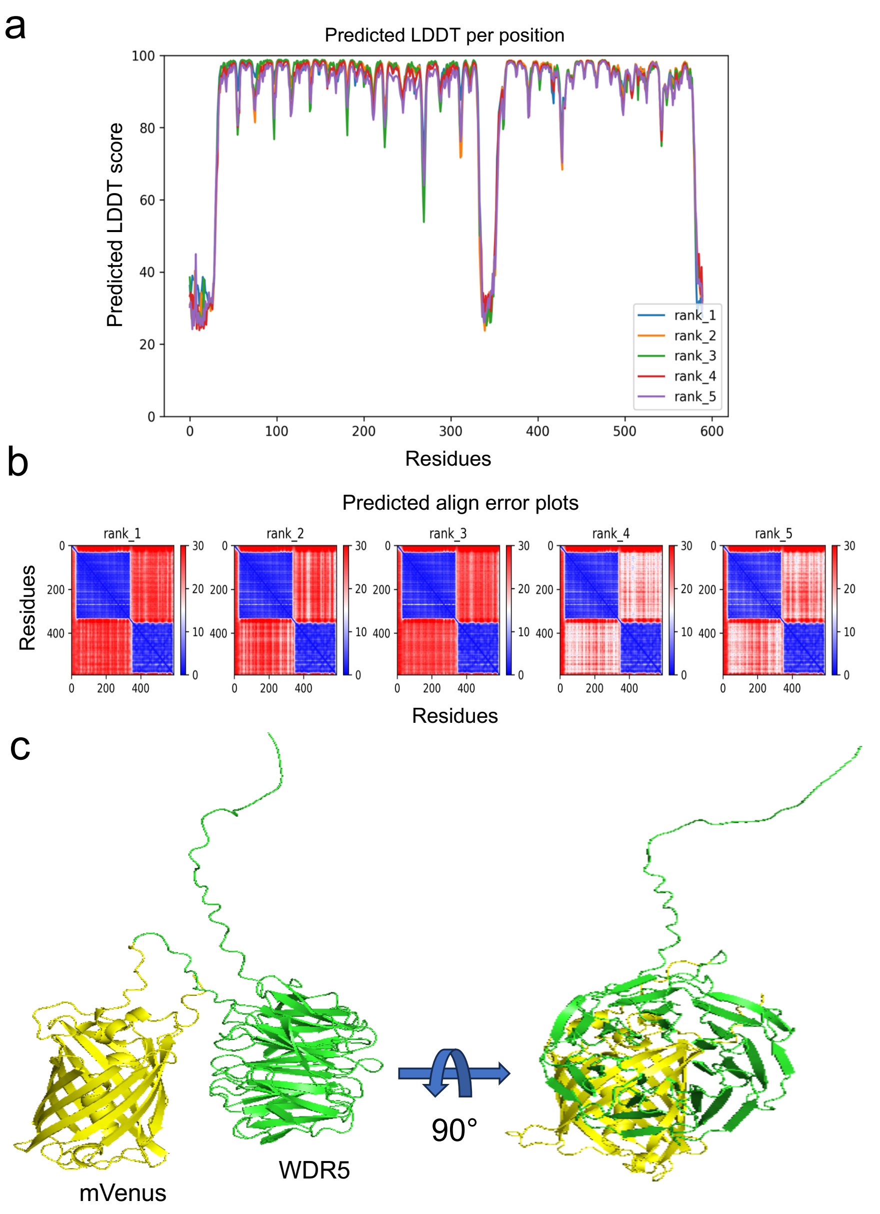

<u>Supplementary Figure S13.</u> The structural prediction of the WDR5-mVenus fusion protein using AlphaFold 2. (a) The predicted Local Distance Difference Test (pLDDT) confidence score per residue was between 80 and 100 for most residues except the N-terminal IDR and the linker between the two proteins. **(b)** The predicted aligned error (PAE) was determined for each residue of the WDR5-mVenus fusion protein. The PAE was low for most residues except for the N-terminal IDR and the linker between the two proteins. For these two regions, a low pLDDT score coupled with a high PAE highlights a high degree of disorder. **(c)** The structure of WDR5-mVenus, as predicted by AlphaFold 2,[13-15] shows the folded and disordered regions of WDR5 (green) and mVenus (yellow) using the side (*left*) and top (*right*) views.

10. Phase separation assays of WDR5 in a cell-free environment.

Supplementary Table S8. **Osmolarity measurements for the hyperosmotic conditions examined in this study.** The phase separation buffer used in most experiments was 20 mM Tris-HCl, 150 mM KCl, 1 mM TCEP, and pH 7.5, exhibiting an osmolarity of 315 ± 3 mOsmol/l. The phase separation buffer with 10% (w/v) PEG-8k showed an osmolarity of 377 ± 6 mOsmol/l. When the phase separation buffer included 300 mM sorbitol, the osmolarity was 577 ± 6 mOsmol/l. Three independent measurements were used to calculate the reported means. Data indicate mean ± s.d.

KCl (mM)	Osmolarity (mOsmol/L)	150 mM KCl and PEG-8k (%)	Osmolarity (mOsmol/L)
**75**	197 ± 5	**5**	342 ± 7
**150**	315 ± 3	**10**	377 ± 6
**300**	644 ± 6	**20**	418 ± 9
**450**	651 ± 8	**30**	441 ± 10

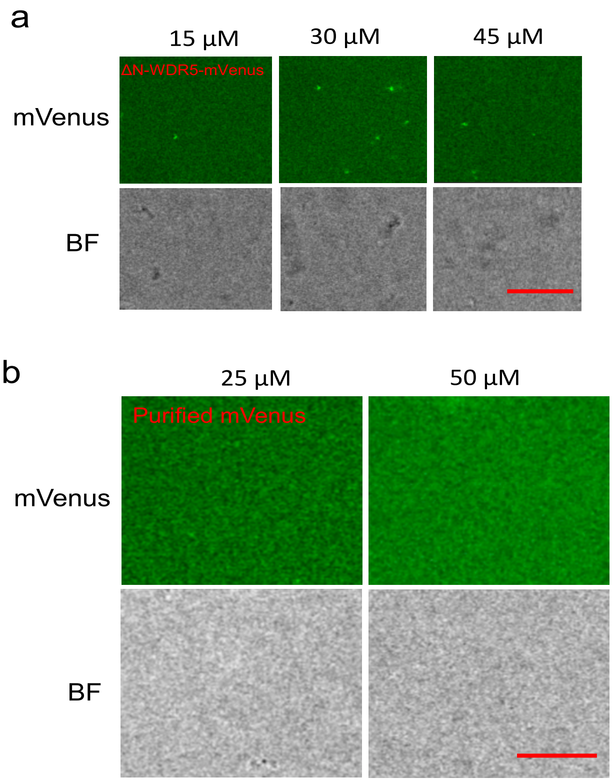

Supplementary Figure S14. **Phase separation assay of ΔN-WDR5-mVenus and mVenus in a cell-free environment. (a)** Images of ΔN-WDR5-mVenus recorded at 448 nm and brightfield (BF) channels. Images were collected at 15, 30, and 45 µM ΔN-WDR5-mVenus. This experiment was independently repeated three times with similar results. **(b)** Images of mVenus were recorded at 488 nm and BF channels. Images were collected at 25 and 50 µM mVenus. The droplet formation was examined using the phase separation buffer (20 mM Tris-HCl, 150 mM KCl, 1 mM TCEP, and pH 7.5) with 10% (w/v) PEG-8k. This experiment was independently repeated three times with similar results. The horizontal scale bar is 10 µm.

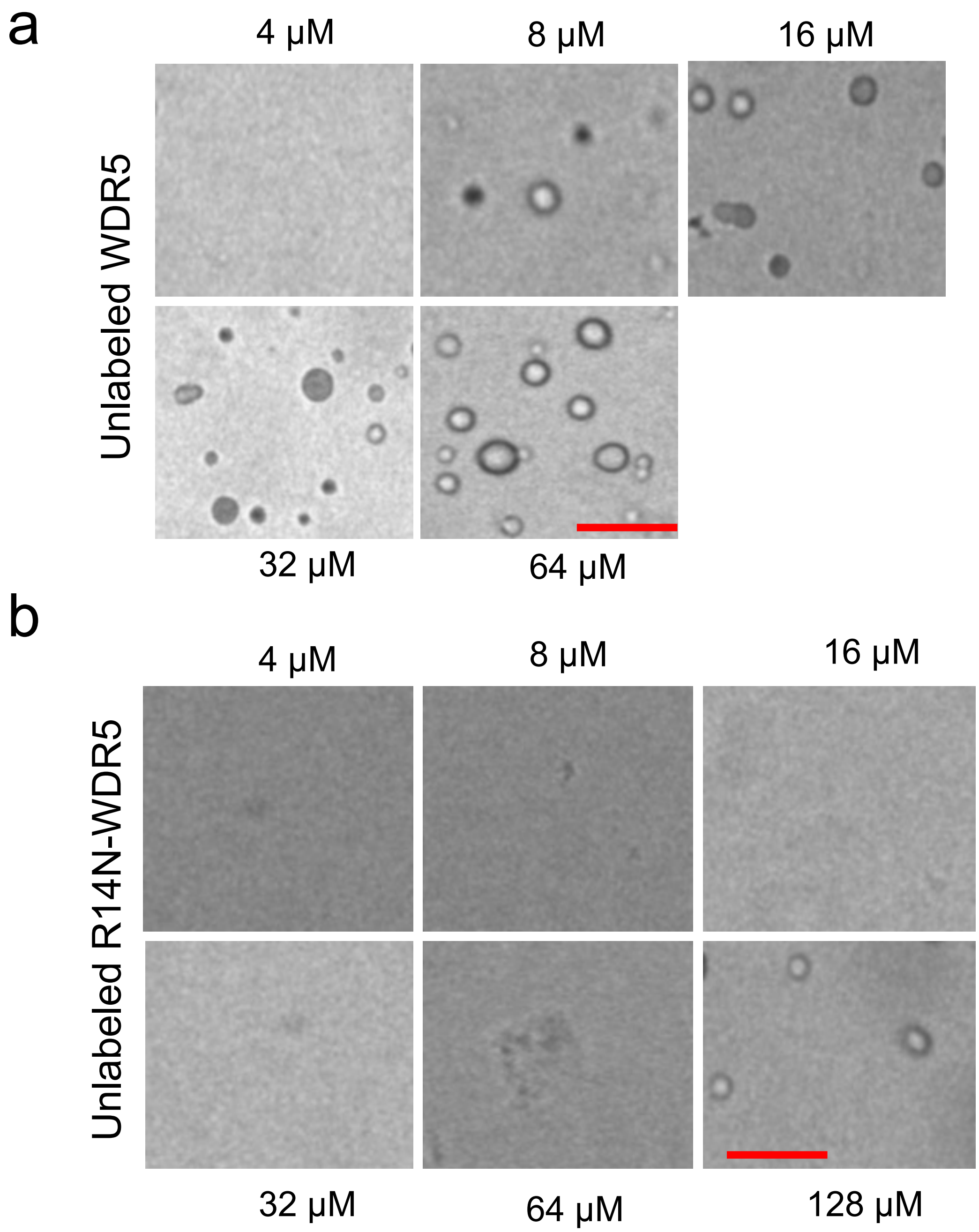

<u>Supplementary Figure S15.</u> Phase separation assay of unlabeled proteins in a cell-free environment. (a) Images of unlabeled WDR5 droplets recorded at brightfield channels using various [WDR5] values. Arrows indicate the fusion of two droplets. **(b)** Images of the unlabeled R14N-WDR5 mutant were recorded the same way as in (a). The droplet formation was examined using the phase separation buffer (20 mM Tris-HCl, 150 mM KCl, 1 mM TCEP, and pH 7.5) with 10% (w/v) PEG-8k. These experiments were independently repeated three times with similar results. The horizontal scale bar is 10 μm.

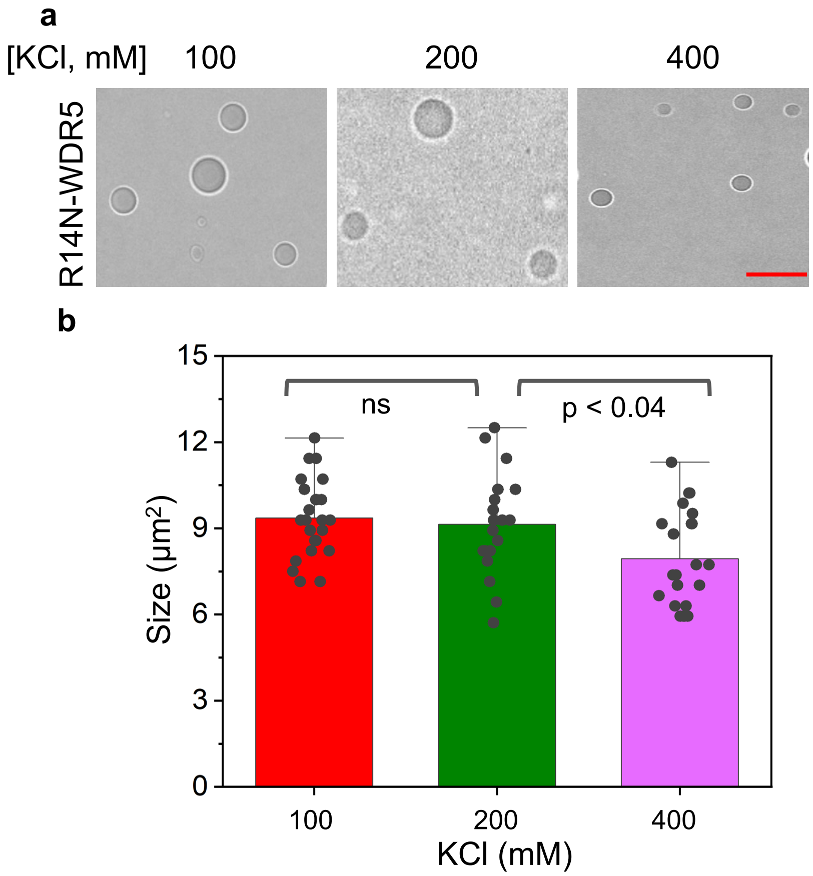

<u>Supplementary Figure S16.</u> The effect of the KCl concentration on the phase separation of R14N-WDR5. (a) Images of R14N-WDR5 droplets were recorded at brightfield channels using 150 μM R14N-WDR5. The horizontal scale bar was 10 μm. **(b)** The droplet size was determined at various [KCl] values (mean ± s.d., n = 24, 19, 22 droplets for 100, 200, and 400 mM KCl, respectively). The phase separation buffer included 10% (w/v) PEG-8k. A two-tailed unpaired t-test was used in b.

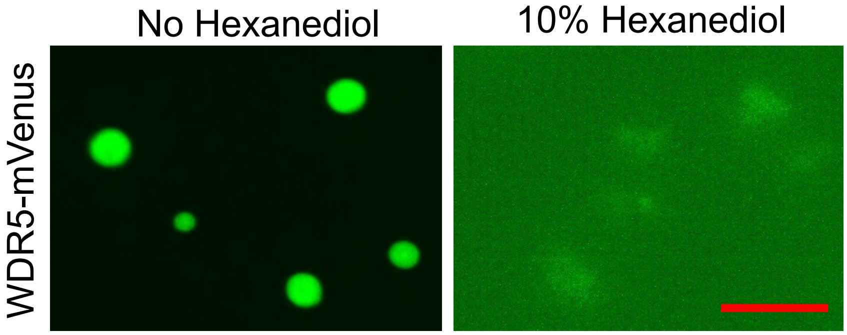

<u>Supplementary Figure S17.</u> The effect of 10% (v/v) hexanediol on WDR5-mVenus droplets. 20 μM WDR5-mVenus was used. The droplet formation was examined using the phase separation buffer (20 mM Tris-HCl, 150 mM KCl, 1 mM TCEP, and pH 7.5) with 10% (w/v) PEG-8k. These experiments were independently repeated n = 3 times with similar results. The horizontal scale bar is 10 μm.

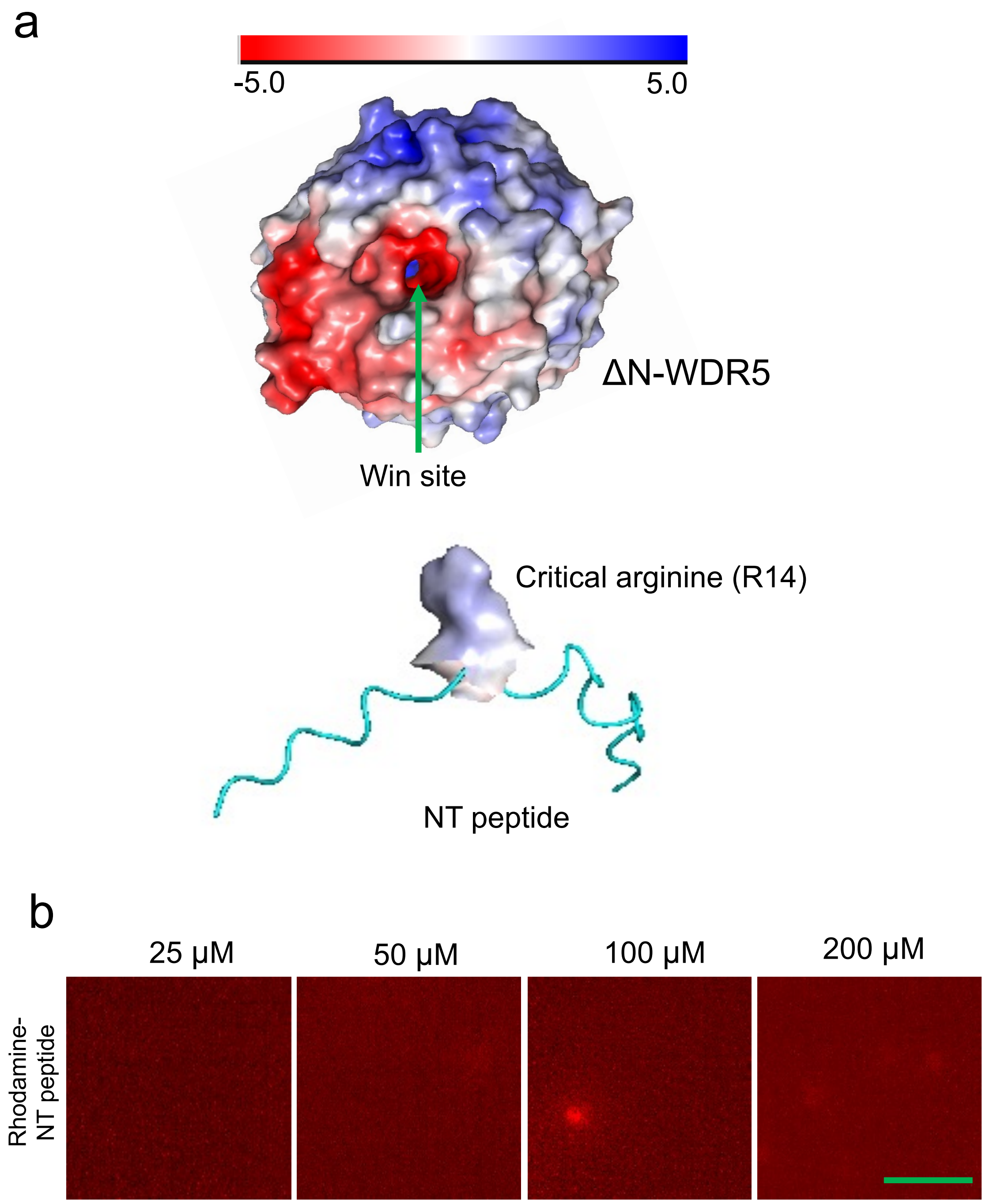

<u>Supplementary Figure S18.</u> The NT peptide's charge distribution and the phase separation assay in a cell-free environment. (a) The charge distribution of ΔN-WDR5 and the NT peptide, highlighting the negatively charged Win site of ΔN-WDR5 and the positively charged Arg-14 of the NT peptide. **(b)** Rhodamine-labeled NT peptide was diluted in the phase separation buffer (20 mM Tris-HCl, 150 mM KCl, 1 mM TCEP, and pH 7.5) with 10% (w/v) PEG-8k. Images were recorded at various NT concentrations. The horizontal scale bar is 10 μm.

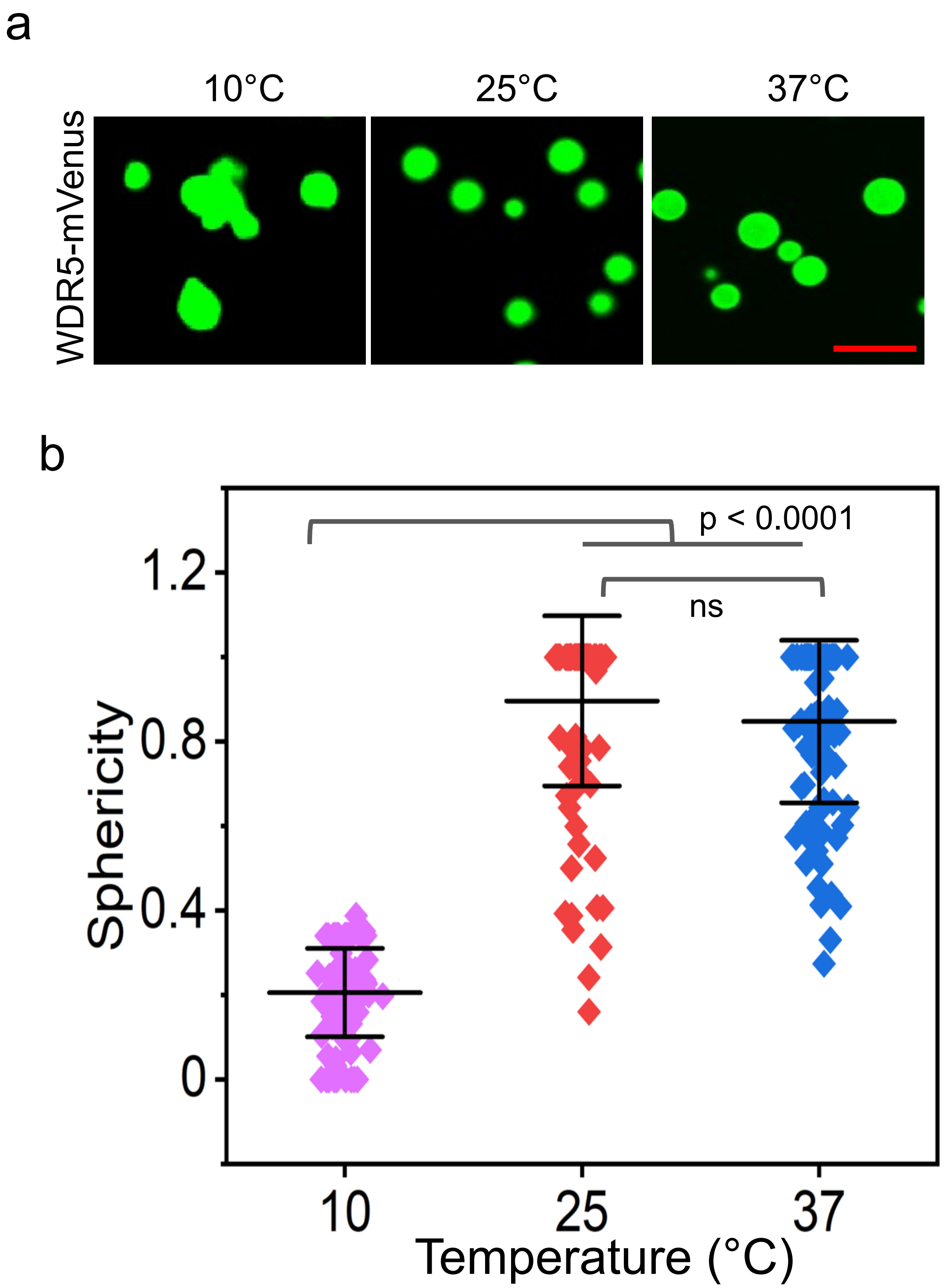

Supplementary Figure S19. **Temperature dependence of WDR5 droplets. (a)** Images were captured at various distant temperatures. The concentration of WDR5-mVenus was 25 µM. The experiment was independently repeated n = 3 times with similar results. The horizontal scale bar is 10 µm. **(b)** Sphericity of WDR5 condensates at various temperatures using ImageJ. Sphericity varies between 0 and 1. 0 and 1 denote sphericity values for very aspherical and spherical droplets, respectively. In each case, the number of droplets, $n = 108$, was selected from three independent experiments for a direct comparison. A two-tailed unpaired t-test was used in b. The center of the data is the mean. Error bars show s.e.m.

Supplementary Table S9. The droplet sphericity at various temperatures and incubation times. Values were obtained using confocal images of droplets, as shown in **Supplementary Figures S19-S20**. Triplicates were performed, and the resultant mean ± s.d values were shown.

Temperature (°C)	Sphericity
10	0.22 ± 0.01
25	0.90 ± 0.04
37	0.88 ± 0.05
**Time (min)**	**Sphericity**
0	0.91 ± 0.03
1	0.64 ± 0.02
2	0.50 ± 0.01
3	0.37 ± 0.01

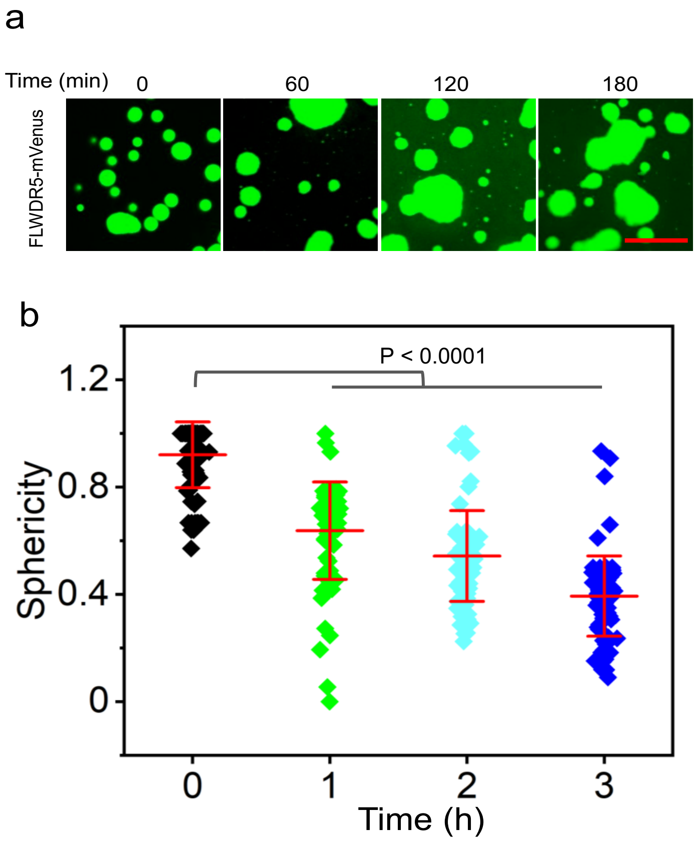

Supplementary Figure S20. Dependence of the droplet sphericity on the incubation time. (a) Images were captured at various times. The concentration of WDR5-mVenus was 55 µM. The experiment was independently repeated n = 3 times with similar results. The horizontal scale bar is 10 µm. **(b)** Sphericity of WDR5 condensates at various time points using ImageJ. Sphericity varies between 0 and 1. 0 and 1 denote sphericity values for very aspherical and spherical droplets, respectively. In each case, the number of droplets, $n = 89$, was selected from three independent experiments for a direct comparison. A two-tailed unpaired t-test was used in (b). The center of the data is the mean. Error bars show s.e.m.

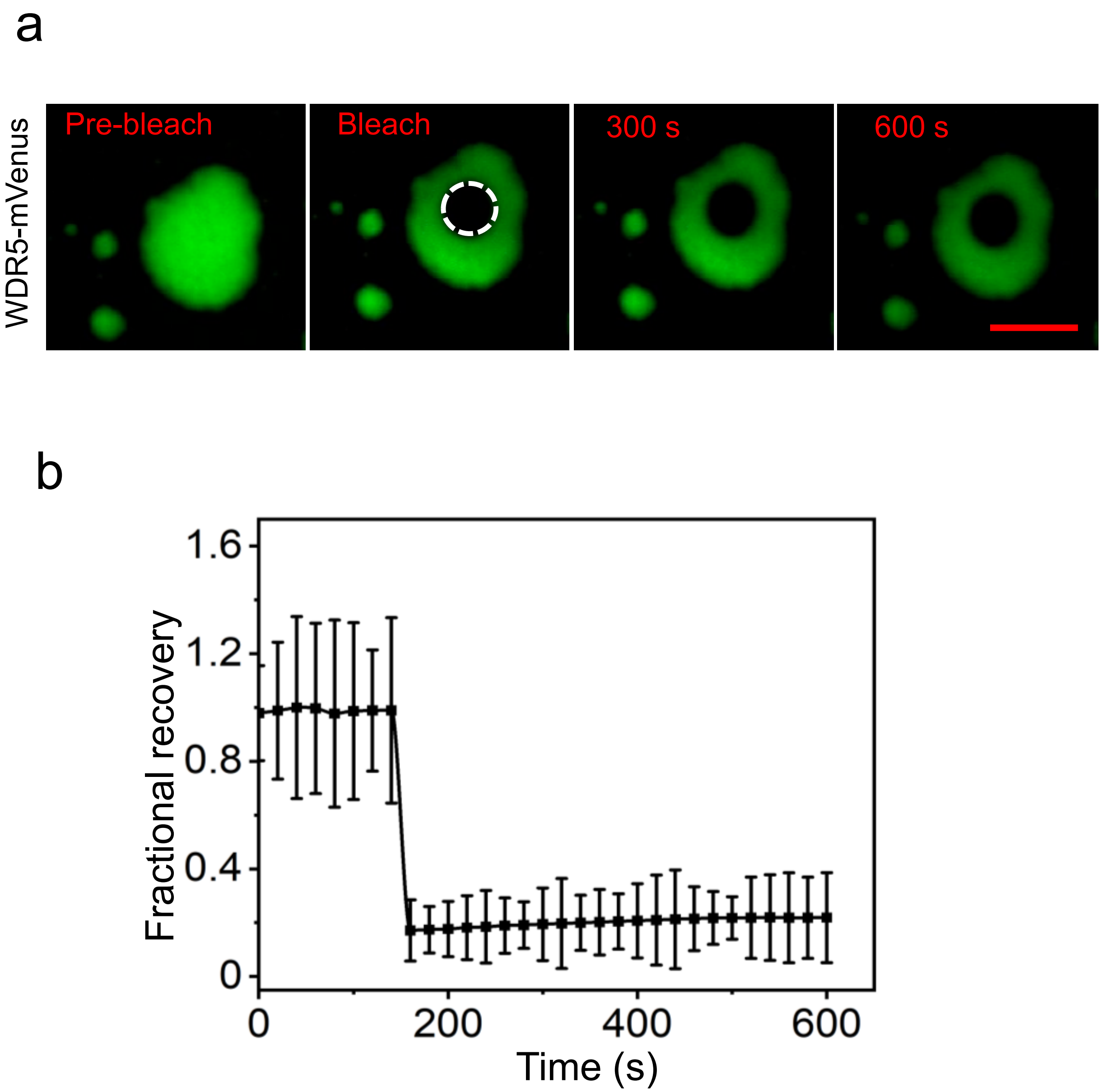

<u>Supplementary Figure S21.</u> Fluorescence recovery after photobleaching (FRAP) signals of WDR5 droplets. (a) A sequence of FRAP images with a droplet before bleach, at bleach, and recovery phases. Here, an aspherical droplet from a sample incubated for 180 minutes at 25°C was selected for photobleaching. The horizontal scale bar is 10 µm. **(b)** Quantifying the FRAP signal normalized to the maximum intensity. 6 droplets were selected from *n* = 3 independent experiments. Data represent mean ± s.d.

11. Liquid-liquid phase separation of WDR5 in HEK-293T cells.

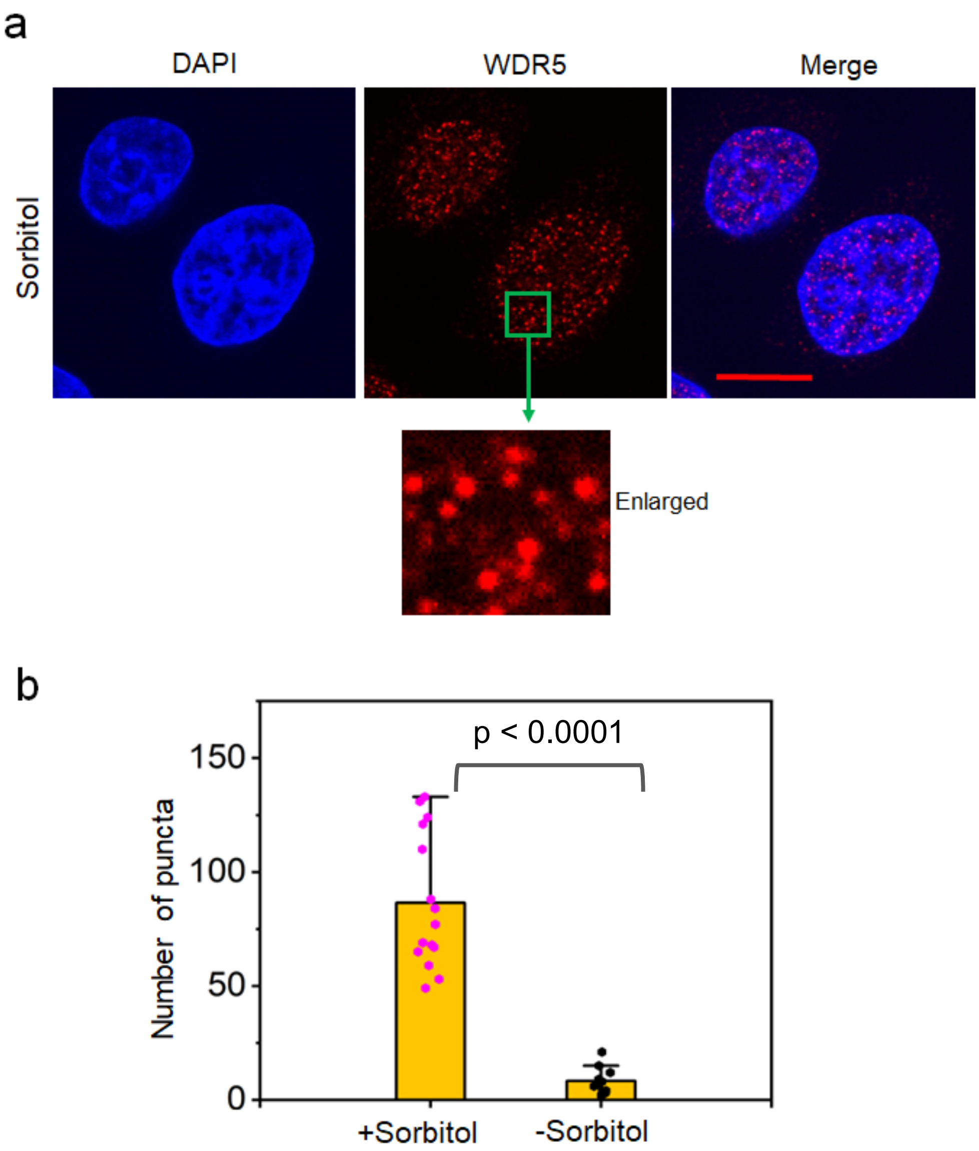

Supplementary Figure S22. WDR5 forms LLPS in HEK-293T cells. **(a)** The immunostaining for endogenous WDR5 in HEK-293T cells shows that WDR5 forms NP under hyperosmotic conditions. The osmotic stress was induced by incubating the cells in 300 mM sorbitol for 25 min, a condition featuring an osmolarity of 577 ± 6 mOsmol/L. Magnification of boxed region was shown to visualize punctate structures. The horizontal scale bar is 10 µm. **(b)** The number of puncta with and without sorbitol. A two-tailed unpaired t-test was used in (b). Each dot represents one cell (n = 15 (with sorbitol); n = 12 (without sorbitol)). The center of the data is the mean. Error bars show s.e.m.

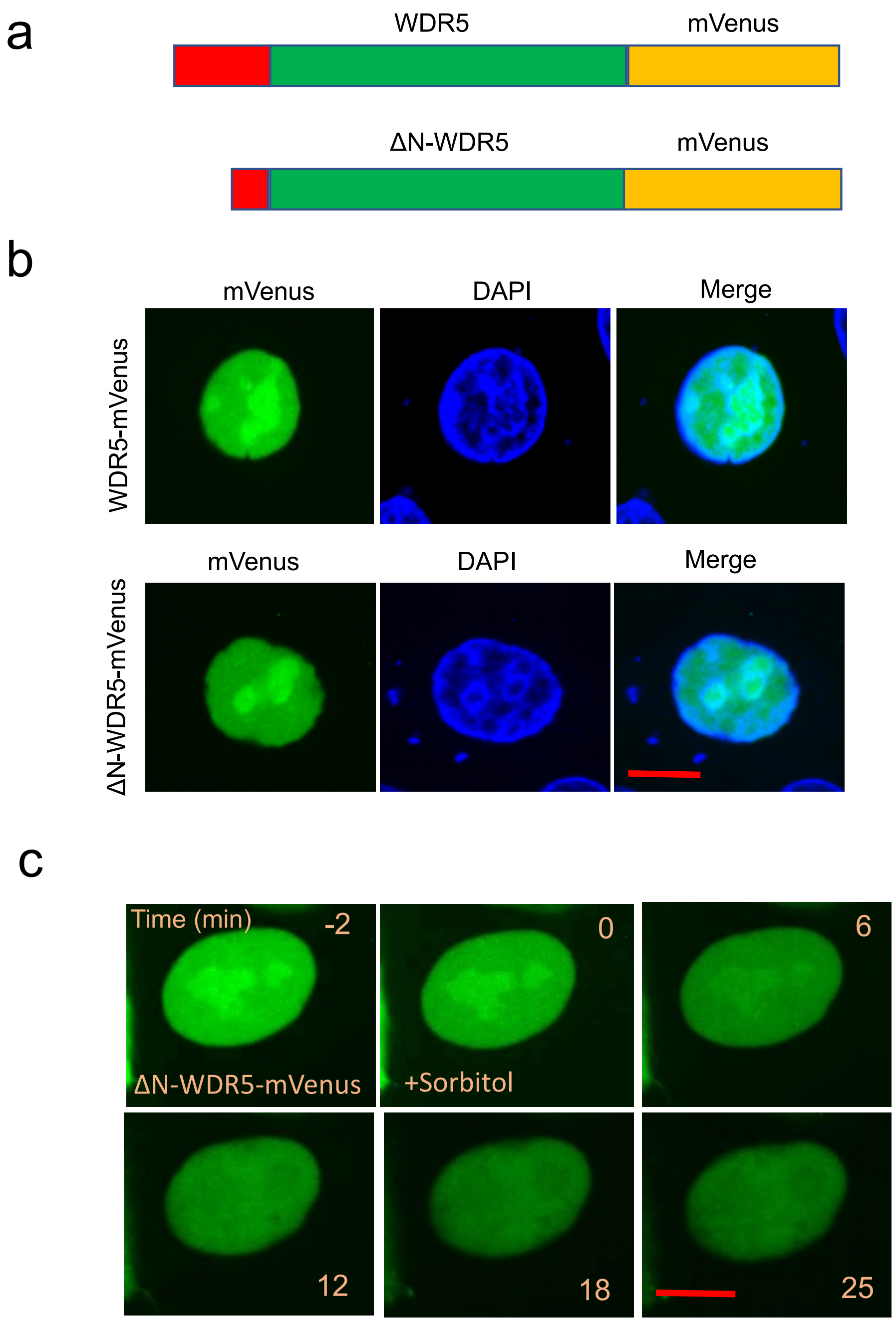

<u>Supplementary Figure S23.</u> Localization of WDR5 and ΔN-WDR5. (a) Schematic representations of WDR5-mVenus construct (*top*) and ΔN-WDR5-mVenus construct (23 residues deleted from the N-terminus of WDR5) (*bottom*). The red sections indicate the unstructured regions of these fusion proteins. These regions include 33 residues in the top construct and 10 in the bottom. **(b)** Images of HeLa cells showing the localization of WDR5-mVenus (*top*) and ΔN-WDR5-mVenus (*bottom*). **(c)** Time-lapse images of HeLa cells expressing ΔN-WDR5-mVenus were recorded in 300 mM sorbitol. The horizontal scale bar is 10 μm.

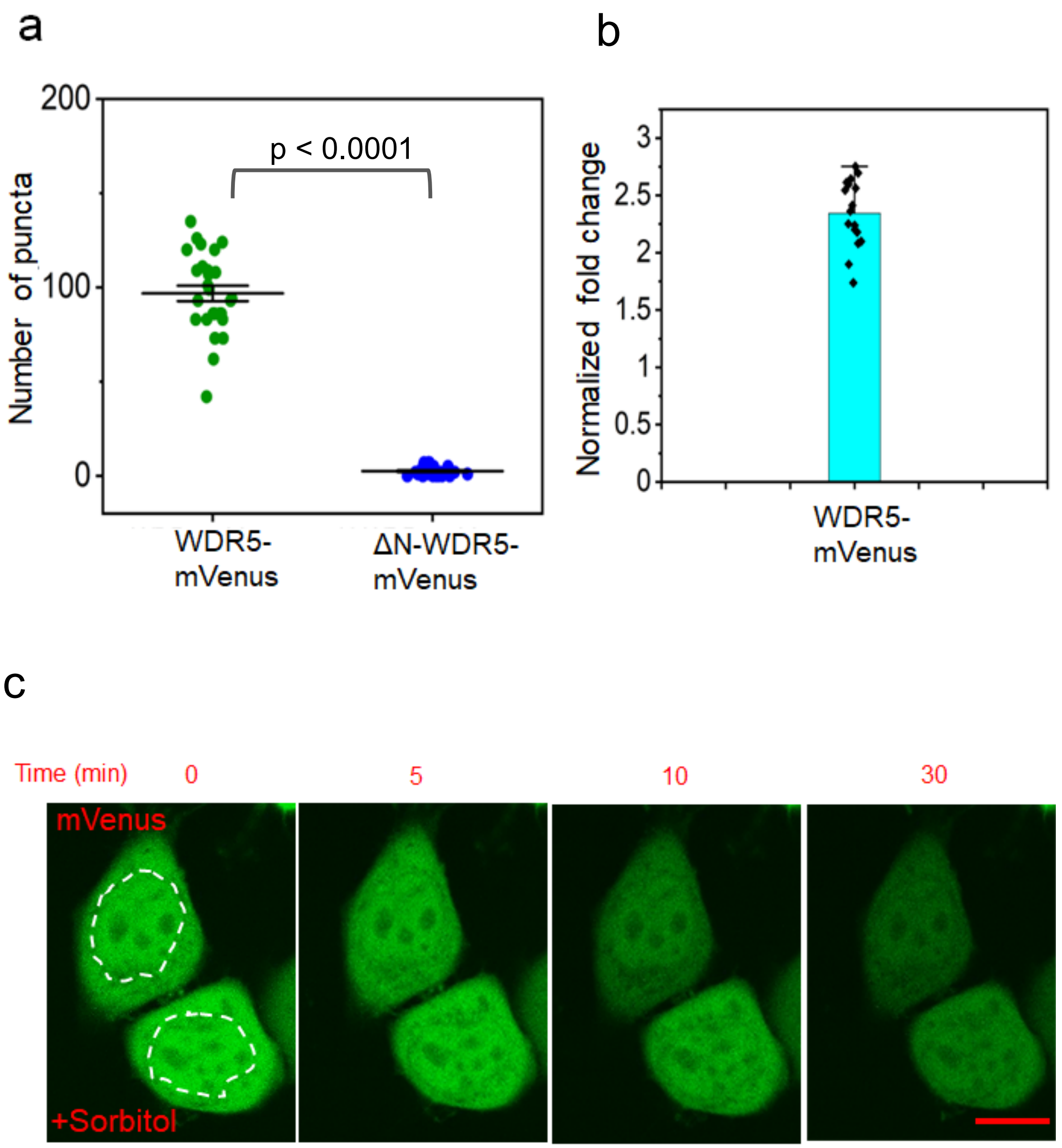

<u>**Supplementary Figure S24.**</u> **Effect of sorbitol on WDR5 puncta in exogenously expressing HeLa cells. (a)** The number of puncta was calculated in WDR5-Venus (n = 27) and ΔN-WDR5-mVenus (n = 31) expressing cells. Cells were treated with 300 mM sorbitol. **A t**wo-tailed unpaired t-test was used in (a). The center of the data is the mean. Error bars show s.d. **(b)** After sorbitol treatment, the accumulation of WDR5 was analyzed, and the change normalized with respect to the nucleoplasm value was calculated (n = 17). **(c)** The effect of sorbitol on mVenus was tested in HeLa cells. Images of HeLa cells expressing the mVenus only were recorded at various time points in the presence of 300 mM sorbitol. The horizontal scale bar is 10 µm.

12. Fluorescence recovery after photobleaching of WDR5 condensates in cell-free environment.

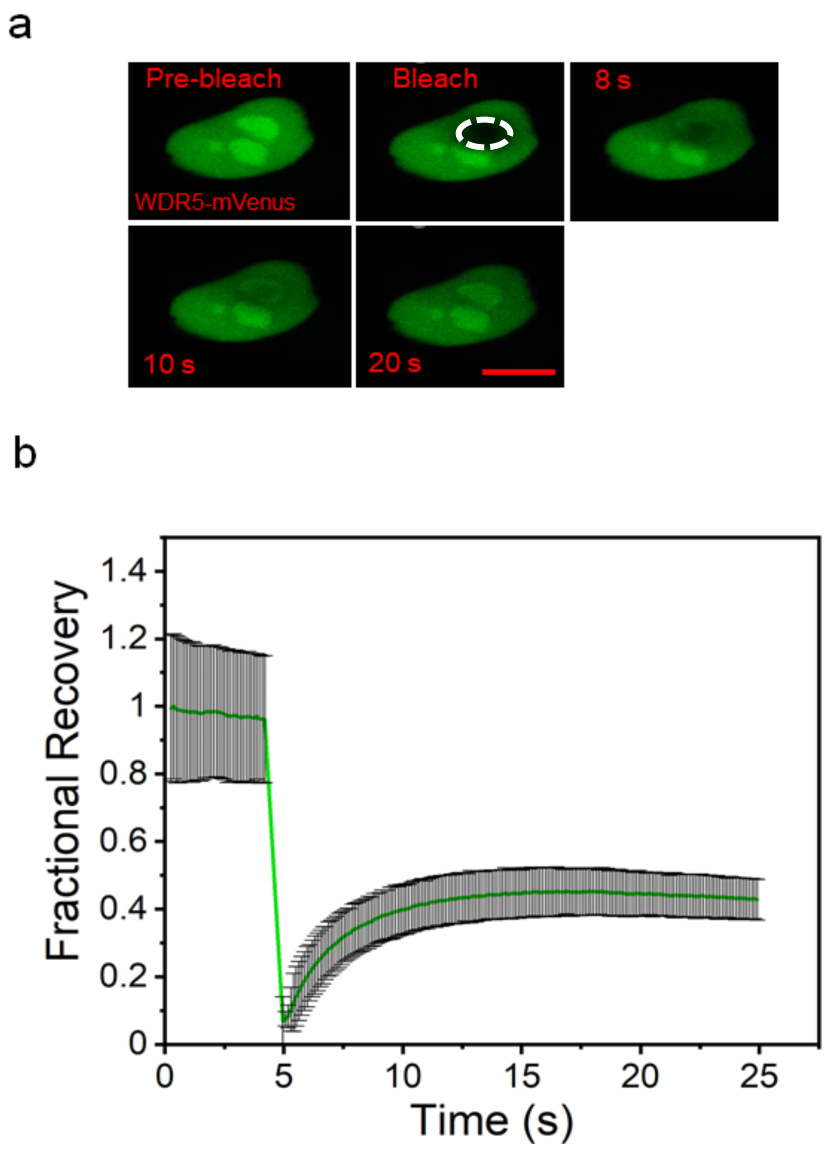

<u>Supplementary Figure S25.</u> The FRAP signal under unstressed conditions. (a) The sequence of the FRAP image sequence with the droplet before bleach, at bleach, and during its recovery phases. The horizontal scale bar is 10 μm. **(b)** The quantification of fractional recovery over time was normalized to the maximum intensity, showing a half-time of ~2.1 s and an overall recovery of ~44% (*n* = 5 different cells selected from three independent experiments).

13. The purification and characterization of the MYC-mScarlet-I protein.

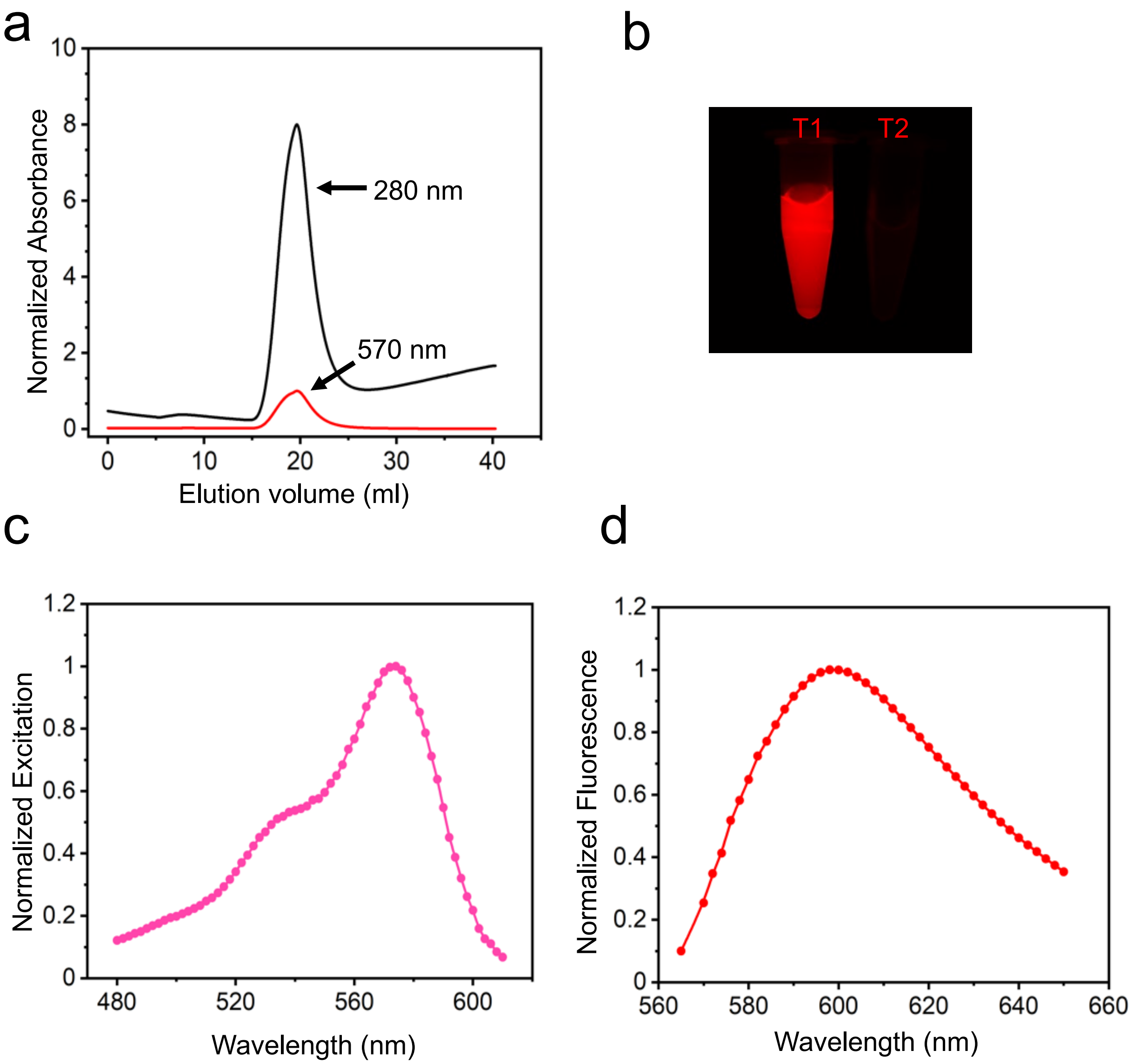

Supplementary Figure S26. Characterization of MYC-mScarlet-I. (a) A chromatogram of the purified MYC-mScarlet-I fusion protein shows the normalized absorbance values at 280 and 570 nm wavelengths. **(b)** An image of the MYC-mScarlet-I-containing microfuge tube (T1) and buffer (T2) was captured using an MP imaging system (Bio-Rad, Hercules, CA) using the Cy3 filter. **(c)** The excitation spectrum of the purified MYC-mScarlet-I protein was recorded in the 480-610 nm range using a SpectraMax i3 microplate reader (Molecular Devices). **(d)** The emission spectrum of the purified MYC-mScarlet-I protein was recorded in the 565-650 nm range using a SpectraMax i3 microplate reader (Molecular Devices).

14. The fusion, fission, and FRAP analysis of MYC droplets.

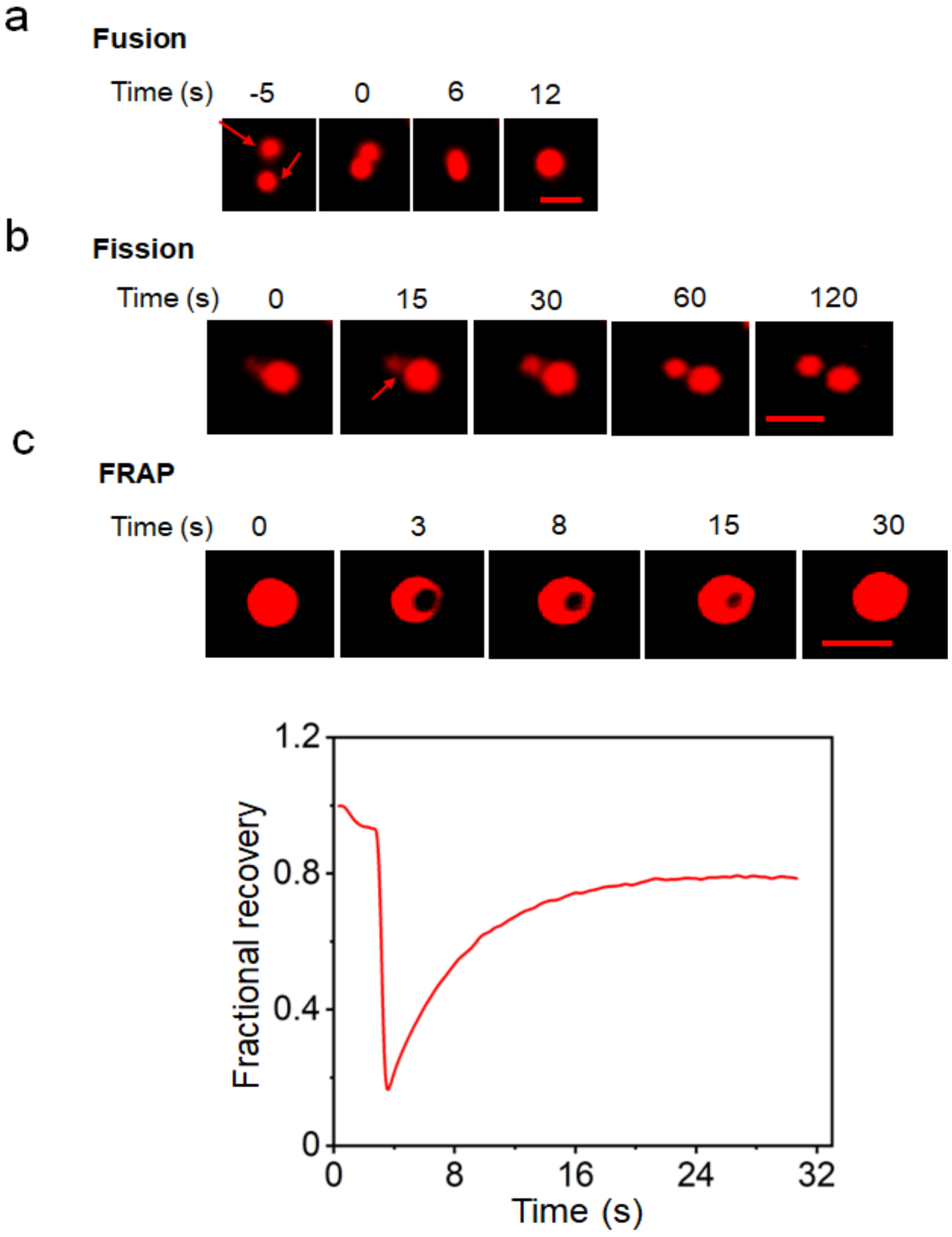

<u>Supplementary Figure S27.</u> The fusion, fission, and FRAP analysis of MYC droplets. **(a)** MYC droplet coalescence is shown in fluorescence illumination. The time dependence of the image sequence of merging droplets is presented. 15 µM MYC-mScarlet-I was added to the phase separation buffer. **(b)** The fission behavior of MYC droplets was shown as a function of time. 15 µM MYC-mScarlet-I was added to the phase separation buffer. **(c)** A FRAP image sequence with an MYC droplet before bleach (t = 0 s), at bleach (t = 3 s), and the overall recovery (t = 4 - 30 s) (*top*). Quantification of the FRAP signal normalized to the maximum fluorescence intensity (*bottom*). These experiments have been independently repeated n = 3 times with similar results. The horizontal scale bar is 10 µm.

15. The effect of a peptide inhibitor on the heterotypic MYC-WDR5 condensate.

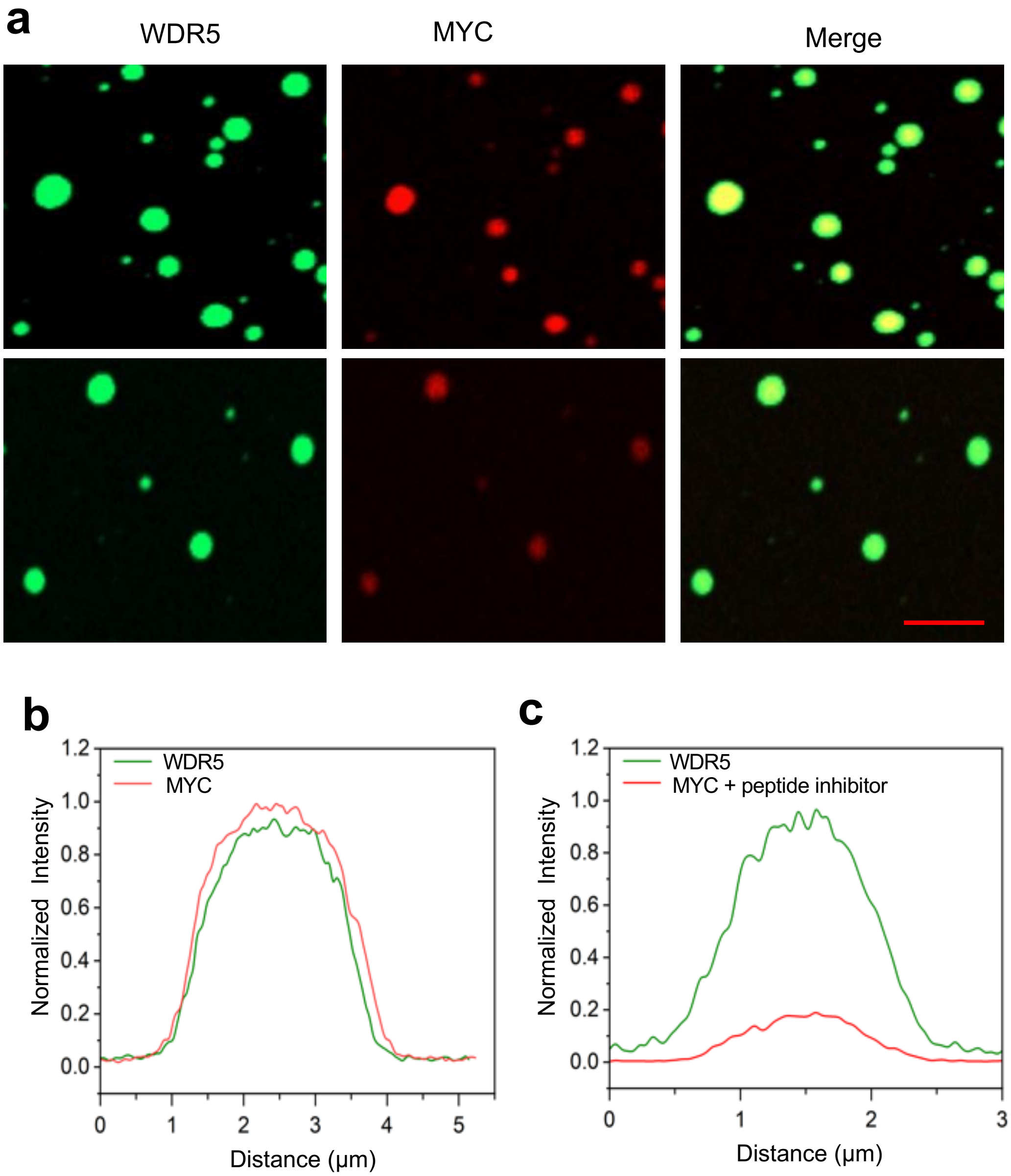

<u>Supplementary Figure S28.</u> The effect of a peptide inhibitor on the co-phase separation of MYC and WDR5. (a) 20 μM of each of MYC-mScarlet-I and WDR5-mVenus were added to the phase separation buffer, and fluorescence imaging was performed. In the upper panels, images show that MYC was recruited into WDR5 droplets. A competitive peptide based on the MYC-interacting region with WDR5 (EEEIDVVSV)[16] was added at a concentration of 30 μM to the phase separation buffer containing the droplets. In this case, images were also acquired, as illustrated in the bottom panels. **(b)** An intensity curve of a droplet containing WDR5 and MYC without an inhibitor. **(c)** An intensity curve of a droplet containing WDR5 and MYC with the peptide inhibitor. Red and green denote MYC and WDR5, respectively. In (a) and (b), the horizontal scale bar is 5 μm. This experiment was independently repeated $n = 3$ times with similar results.

16. Colocalization of Amyotrophic lateral sclerosis (ALS)-linked proteins with WDR5 condensates under hyperosmotic conditions.

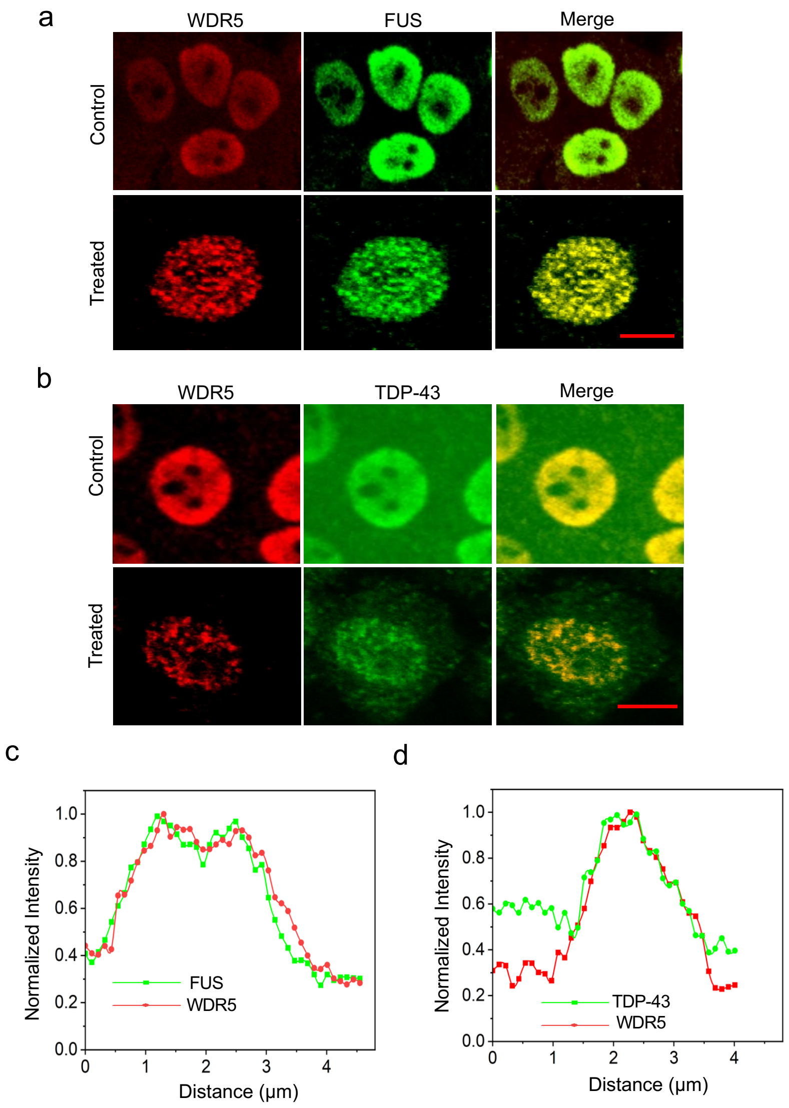

<u>Supplementary Figure S29.</u> **Amyotrophic lateral sclerosis (ALS)-linked proteins were colocalized with WDR5 condensates under hyperosmotic stress. (a)** The co-immunostaining for endogenous FUS and WDR5 in HeLa cells shows that WDR5 forms nuclear puncta (NP) under hyperosmotic conditions, and those punctate structures were enriched in FUS. The osmotic stress was induced by incubating the cells in 300 mM sorbitol for 40 minutes. **(b)** The co-immunostaining for endogenous TDP-43 and WDR5 in HeLa cells shows that WDR5 forms NP under hyperosmotic conditions, and those punctate structures recruited TDP-43. Images are representative examples from three independent experiments. **(c)** The intensity profile of a punctate structure shows the distribution of WDR5 and FUS in the liquid-like condensates. **(d)** The intensity profile of a punctate structure shows the distribution of WDR5 and TDP-43 in the liquid-like condensates. In a and b, the horizontal scale bar is 10 µm.

17. In vitro sequestration of RNA in WDR5 droplets.

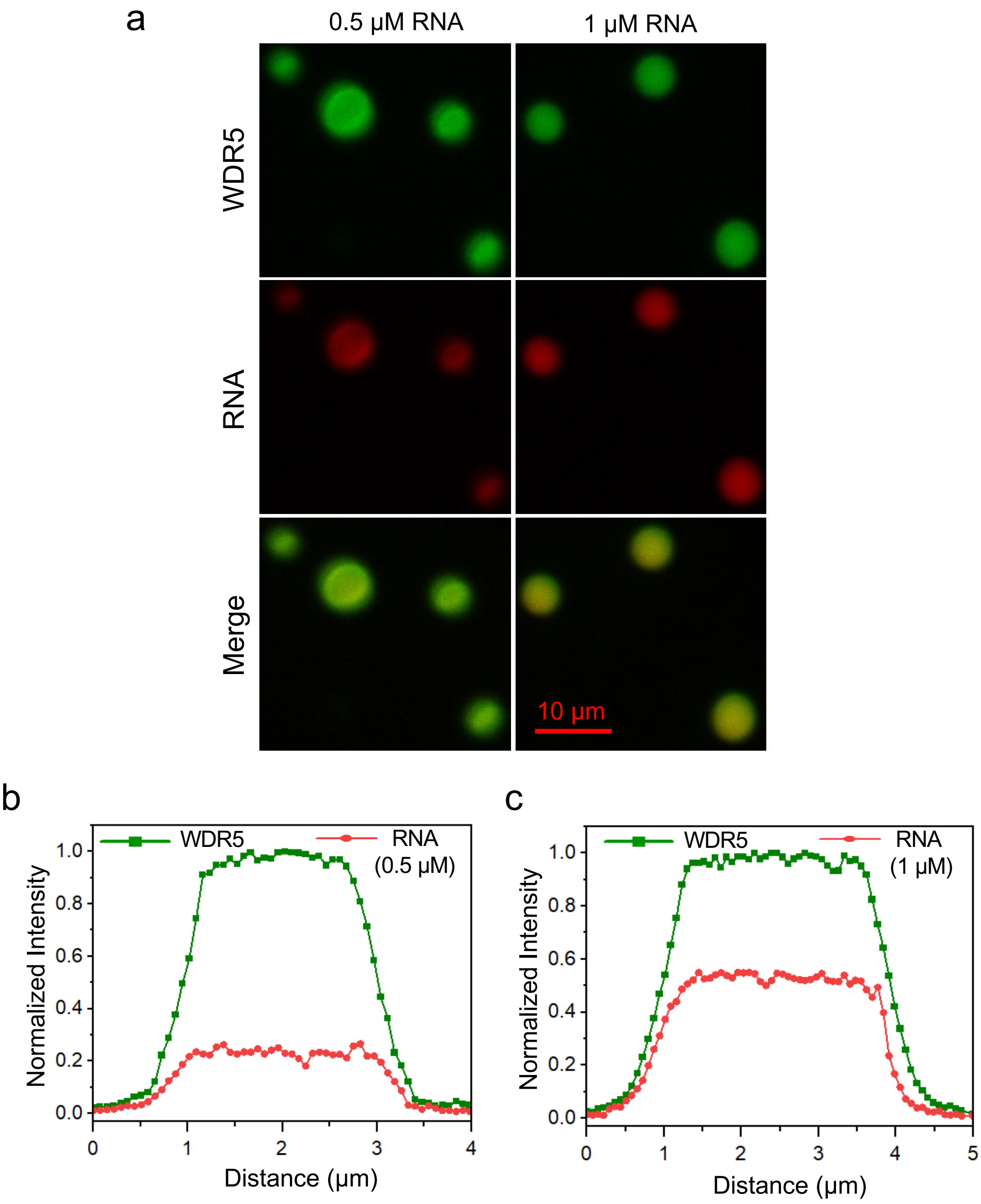

<u>Supplementary Figure S30.</u> *In vitro* sequestration of RNA in WDR5 droplets. (a) Images of 10 μM phase-separated WDR5 and Alexa Fluor 568 (AF568)-labeled RNA (0.5 and 1 μM). Images show that RNA was accumulated into WDR5 condensates. The horizontal scale bar is 10 μm. **(b)** The intensity profile of a confocal slice through the droplet's center was plotted for WDR5 + 0.5 μM RNA. **(c)** The same as in b but plotted for WDR5 + 1 μM RNA.

Supplementary Table S10. Partition coefficient (*Π*) of RNA in wild-type and mutant WDR5 droplets. The phase separation buffer used in these experiments was 20 mM Tris-HCl, 150 mM KCl, 1 mM TCEP, and pH 7.5 with 10% (w/v) PEG-8k. Droplets were selected from three independent measurements to calculate the reported means. *n* is the number of droplets. Data indicate mean ± s.d.

Sample	*Π*	*n*
RNA in WDR5 droplets	16.5 ± 4.6	19
RNA in F266A-WDR5 droplets	4.0 ± 1.1	21

Supplementary Table S11. Partition coefficient (*Π*) of WDR5 and F266A-WDR5 in the presence and absence of RNA. The phase separation buffer used in these experiments was 20 mM Tris-HCl, 150 mM KCl, 1 mM TCEP, and pH 7.5, 10% (w/v) PEG-8k with and without RNA. Droplets were selected from three independent measurements to calculate the reported means. *n* is the number of droplets. Data indicate mean ± s.d.

Sample	RNA	*Π*	*N*
WDR5	+	58.2 ± 7.1	14
WDR5	-	26.6 ± 3.1	22
F266A-WDR5	+	28.3 ± 3.0	17
F266A-WDR5	-	24.2 ± 2.8	15

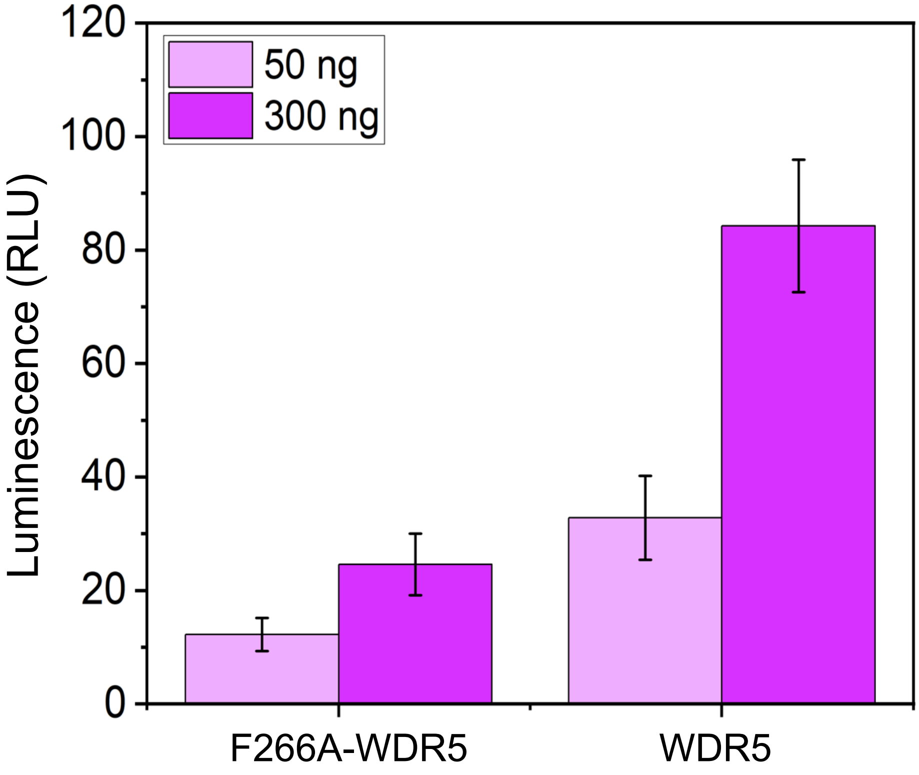

Supplementary Figure S31. Activation of the target gene by WDR5. Wild-type WDR5 generated a high amount of the luciferase gene product, whereas the non-RNA binding mutant (F266A-WDR5) is defective in activating luciferase expression, as shown by the significantly reduced luminescence.

18. List of primers utilized in this study.

Supplementary Table S12. List of primers utilized in this study. F and R denote the forward and reverse primers, respectively.

Gene name	Sequences of primers
**wdr5-mvenus _F**	TCCGCTAGCATGGCGACGGAGGAGAAGAAGCCCGAG
**wdr5-mvenus _R**	TCGAAGCTTTTACTTGTACAGCTCGTCCATGCCG
**Δn-wdr5-mvenus _F**	TCCGCTAGCATGCAGAGCAAGCCTACACCTGTGAAG
**Δn-wdr5-mvenus _R**	TCGAAGCTTTTACTTGTACAGCTCGTCCATGCCG
**myc-mscarlet-i_F**	TCCGCTAGCATGCCCCTCAACGTTAGCTTCACCAACAGG
**myc-mscarlet-i_R**	TCGAAGCTTTTACTTGTACAGCTCGTCCATGCCG
**mvenus_F**	TCCGCTAGCATGGTGAGCAAGGGCGAGGAGC
**mvenus_R**	TCGAAGCTTTTACTTGTACAGCTCGTCCATGCCGAG
**wdr5_F**	TCCGCTAGCATGGCGACGGAGGAGAAGAAGCCCGAGACC
**wdr5_R**	GCGACCGGTCCGGAGCCGCCGCAGTCACTCTTCCACAGTTTAATT
**Δn-wdr5_F**	TCCGCTAGCATGCAGAGCAAGCCTACACCTGTGAAG
**Δn-wdr5_R**	GCGACCGGTCCGGAGCCGCCGCAGTCACTCTTCCACAGTTTAATT
**myc_F**	TCCGCTAGCATGTCCTACCAGGCTGCGCGCAAAGAC
**myc_R**	GCGACCGGTCCGGAGCCGCCGCTGATCTGTCTCAGGACTCTG
**r14n-wdr5_F**	GAGGCCGCCAACGCACAGCCAA
**r14n-wdr5_R**	GGTCTCGGGCTTCTTCTCCTCCGTCGC
**f266a-wdr5_F**	ATTTGCCAATGCCTCTGTTACTG
**f266a-wdr5_R**	ATGCAGTATTTCTCATTCTTGTGGC

19. Supplemental references.

(1) Abramson, J.; Adler, J.; Dunger, J.; Evans, R.; Green, T.; Pritzel, A.; Ronneberger, O.; Willmore, L.; Ballard, A. J.; Bambrick, J.; et al. Accurate structure prediction of biomolecular interactions with AlphaFold 3. *Nature* **2024**, *630* (8016), 493-500. DOI: 10.1038/s41586-024-07487-w From NLM.

(2) Thomas, L. R.; Wang, Q.; Grieb, B. C.; Phan, J.; Foshage, A. M.; Sun, Q.; Olejniczak, E. T.; Clark, T.; Dey, S.; Lorey, S.; et al. Interaction with WDR5 promotes target gene recognition and tumorigenesis by MYC. *Mol. Cell. Biochem.* **2015**, *58* (3), 440-452. DOI: 10.1016/j.molcel.2015.02.028 From NLM.

(3) Altis, A.; Nguyen, P. H.; Hegger, R.; Stock, G. Dihedral angle principal component analysis of molecular dynamics simulations. *J. Chem. Phys.* **2007**, *126* (24). DOI: 10.1063/1.2746330.

(4) Honorato, R. V.; Trellet, M. E.; Jiménez-García, B.; Schaarschmidt, J. J.; Giulini, M.; Reys, V.; Koukos, P. I.; Rodrigues, J.; Karaca, E.; van Zundert, G. C. P.; et al. The HADDOCK2.4 web server for integrative modeling of biomolecular complexes. *Nat. Protoc.* **2024**, *19* (11), 3219-3241. DOI: 10.1038/s41596-024-01011-0 From NLM.

(5) Patel, A.; Vought, V. E.; Dharmarajan, V.; Cosgrove, M. S. A conserved arginine-containing motif crucial for the assembly and enzymatic activity of the mixed lineage leukemia protein-1 core complex. *J. Biol. Chem.* **2008**, *283* (47), 32162-32175. DOI: 10.1074/jbc.M806317200 From NLM.

(6) Patel, A.; Dharmarajan, V.; Cosgrove, M. S. Structure of WDR5 bound to mixed lineage leukemia protein-1 peptide. *J. Biol. Chem.* **2008**, *283* (47), 32158-32161. DOI: 10.1074/jbc.C800164200 From NLM.

(7) Dharmarajan, V.; Lee, J. H.; Patel, A.; Skalnik, D. G.; Cosgrove, M. S. Structural basis for WDR5 interaction (Win) motif recognition in human SET1 family histone methyltransferases. *J. Biol. Chem.* **2012**, *287* (33), 27275-27289. DOI: 10.1074/jbc.M112.364125 From NLM.

(8) Zhang, P.; Lee, H.; Brunzelle, J. S.; Couture, J. F. The plasticity of WDR5 peptide-binding cleft enables the binding of the SET1 family of histone methyltransferases. *Nucleic Acids Res.* **2012**, *40* (9), 4237-4246. DOI: 10.1093/nar/gkr1235 From NLM.

(9) Imran, A.; Moyer, B. S.; Canning, A. J.; Kalina, D.; Duncan, T. M.; Moody, K. J.; Wolfe, A. J.; Cosgrove, M. S.; Movileanu, L. Kinetics of the multitasking high-affinity Win binding site of WDR5 in restricted and unrestricted conditions. *Biochem. J.* **2021**, *478* (11), 2145-2161. DOI: 10.1042/bcj20210253 From NLM.

(10) Imran, A.; Moyer, B. S.; Wolfe, A. J.; Cosgrove, M. S.; Makarov, D. E.; Movileanu, L. Interplay of Affinity and Surface Tethering in Protein Recognition. *J. Phys. Chem. Lett.* **2022**, *13* (18), 4021-4028. DOI: 10.1021/acs.jpclett.2c00621 From NLM.

(11) Imran, A.; Moyer, B. S.; Kalina, D.; Duncan, T. M.; Moody, K. J.; Wolfe, A. J.; Cosgrove, M. S.; Movileanu, L. Convergent Alterations of a Protein Hub Produce Divergent Effects Within a Binding Site. *ACS Chem. Biol.* **2022**, *17* (6), 1586-1597.

(12) Jachimska, B.; Wasilewska, M.; Adamczyk, Z. Characterization of globular protein solutions by dynamic light scattering, electrophoretic mobility, and viscosity measurements. *Langmuir* **2008**, *24* (13), 6866-6872. DOI: 10.1021/la800548p From NLM.

(13) Jumper, J.; Evans, R.; Pritzel, A.; Green, T.; Figurnov, M.; Ronneberger, O.; Tunyasuvunakool, K.; Bates, R.; Žídek, A.; Potapenko, A.; et al. Highly accurate protein structure prediction with AlphaFold. *Nature* **2021**, *596* (7873), 583-589. DOI: 10.1038/s41586-021-03819-2 From NLM.

(14) Tunyasuvunakool, K.; Adler, J.; Wu, Z.; Green, T.; Zielinski, M.; Žídek, A.; Bridgland, A.; Cowie, A.; Meyer, C.; Laydon, A.; et al. Highly accurate protein structure prediction for the human proteome. *Nature* **2021**, *596* (7873), 590-596. DOI: 10.1038/s41586-021-03828-1 From NLM.

(15) AlQuraishi, M. Machine learning in protein structure prediction. *Curr. Opin. Chem. Biol.* **2021**, *65*, 1-8. DOI: 10.1016/j.cbpa.2021.04.005 From NLM.

(16) Ahmad, M.; Imran, A.; Movileanu, L. Overlapping characteristics of weak interactions of two transcriptional regulators with WDR5. *Int. J. Biol. Macromol.* **2024**, *258* (Pt 2), 128969. DOI: 10.1016/j.ijbiomac.2023.128969 From NLM.
